# Effectiveness of interventions to increase vaccine uptake: component network meta-analysis

**DOI:** 10.1136/bmj-2025-087578

**Published:** 2026-04-15

**Authors:** Sarah R Davies, Annabel L Davies, Julian P T Higgins, Deborah M Caldwell, Zak A Thornton, Elisabeth Aiton, Ifra Ali, Sarah Dawson, Carmel McGrath, Thomas Parkhouse, Lucy Yardley, Julie Yates, Louise Letley, Sharif A Ismail, Hannah Christensen, Clare E French

**Affiliations:** 1 Population Health Sciences, Bristol Medical School, University of Bristol, Bristol, UK; 2 NIHR Health Protection Research Unit in Evaluation and Behavioural Science at University of Bristol, Bristol, UK; 3 NIHR Applied Research Collaboration West (ARC West) at University Hospitals Bristol and Weston NHS Foundation Trust, Bristol, UK; 4 MRC Integrative Epidemiology Unit, University of Bristol, Bristol, UK; 5 NIHR Health Protection Research Unit in Vaccines and Immunisation, Department of Global Health and Development, Faculty of Public Health and Policy, London School of Hygiene and Tropical Medicine, London, UK; 6 Faculty of Health and Applied Sciences, School of Health and Social Wellbeing, University of West England, Bristol, UK; 7 School of Psychology, University of Southampton, Southampton, UK; 8 UK Health Security Agency, London, UK; 9 Department of Global Health and Development, Faculty of Public Health and Policy, London School of Hygiene and Tropical Medicine, London, UK

## Abstract

**Objectives:**

To identify the effective components of interventions to increase vaccine uptake and to explore variations in effectiveness by population group and in relation to the covid-19 pandemic.

**Design:**

Component network meta-analysis.

**Setting:**

Systematic review of randomised controlled trials in high and upper middle income countries.

**Participants:**

237 studies with 570 intervention arms and 4 361 717 participants.

**Interventions:**

Any intervention targeting vaccine recipients or their caregivers aiming to increase demand for, or access to, vaccinations on the UK immunisation schedule. Key content and delivery features of interventions were identified using a bespoke coding framework co-developed with stakeholders.

**Main outcome measures:**

The outcome of interest was vaccine uptake. Bayesian component level meta-regression estimated relative effects of intervention components as ratios of odds ratios with 95% credible intervals (CrIs).

**Results:**

Of the included studies, 110 were at low risk of bias, 96 had some concerns, and 31 were at high risk. 40% (n=1 744 686) of the participants were male. For children, there was evidence of beneficial effects for payments to cover costs (ratio of odds ratios 3.01, 95% CrI 1.49 to 6.06) and decision aids (2.73, 1.14 to 7.06), and some evidence for extended opportunities (1.37, 0.98 to 1.95) and social factors (1.27, 0.99 to 1.65). For adolescents and young adults, there were beneficial effects for personal delivery formats (2.13, 1.09 to 4.40), delivery by community members alongside healthcare professionals (6.42, 1.94 to 25.62), and social factors (2.62, 1.45 to 5.04), and negative effects for decision aids (0.43, 0.18 to 0.98) and human versus non-human interaction (0.47, 0.21 to 1.02). For adults, beneficial effects were shown for human interaction (1.86, 1.42 to 2.45), extended opportunities (1.63, 1.35 to 2.00), help with appointment scheduling (1.38, 1.06 to 1.78), payments to cover costs (1.47, 1.03 to 2.16), and motivational interviewing (1.79, 1.21 to 2.64), and there was some evidence for financial incentives (1.15, 0.99 to 1.35) and information on vaccine safety and/or efficacy (1.15, 0.99 to 1.32). For adults, evidence also showed a negative effect of non-human interaction versus no interaction (0.72, 0.57 to 0.92). Subgroup analyses showed variation for underserved populations and in relation to the covid-19 pandemic (before 2020 and 2020 onwards).

**Conclusion:**

Overall, extended opportunities, appointment scheduling help, financial incentives, payments to cover costs, and motivational interviewing were effective content components of interventions to increase vaccine uptake. Effective delivery components overall were human interaction and delivery by community members alongside healthcare professionals. However, effective components varied by age group, for underserved populations, and in analyses investigating the impact of the covid-19 pandemic. These findings have important implications for designing, optimising, and implementing targeted interventions, highlighting which components are effective across different populations and contexts. Consideration of the economic data on interventions should further support resource informed decision making.

## Introduction

The global decline in vaccine uptake along with the increase in outbreaks of preventable infectious diseases, underscores the critical need to identify and address gaps in vaccination coverage.^1^ A wide range of strategies aimed at vaccine recipients or their caregivers have been implemented aiming to increase vaccine uptake by addressing barriers such as lack of awareness about vaccine recommendations, efficacy, side effects, accessibility, distrust, misinformation, and time and cost related obstacles.^2^ ^3^

Interventions to improve vaccine uptake are typically multifaceted, involving a range of strategies (content components) that can be delivered in various formats (delivery components). Existing reviews tend to group these interventions into broad categories and assess their effect compared with control groups.^4^ ^5^ Although these analyses offer a broad perspective of the overall effectiveness of strategies, in complex interventions the individual intervention components could work independently or in combination to influence the effect of the intervention.^6^ Understanding the effectiveness of the different components and how that might vary across different population groups and contexts, is vital to inform decision making around which interventions to implement given limited resources. Such nuanced insights can also inform the development and optimisation of new interventions.

Component network meta-analysis is one approach for examining the constituent parts of complex interventions.^7^ It is an enhanced form of network meta-analysis that helps to unravel the effects of various components or combinations of components within complex interventions, and it has been used in several areas, such as mental health,^8^ smoking cessation,^9^ and falls prevention.^10^ As yet, component network meta-analysis has not been applied to vaccine uptake interventions. In component network meta-analysis, integral components of interventions are systematically coded using a coding framework, and then all direct and indirect evidence for these components are compared in a single analysis.

We aimed to address the current gap in understanding of the effectiveness of different components of recipient focused vaccine uptake interventions. We developed and applied a bespoke intervention coding framework, using this to code components of interventions included in a set of trials from a systematic review of interventions to increase vaccine uptake. We then conducted a component network meta-analysis.

## Methods

**Fig 1:**
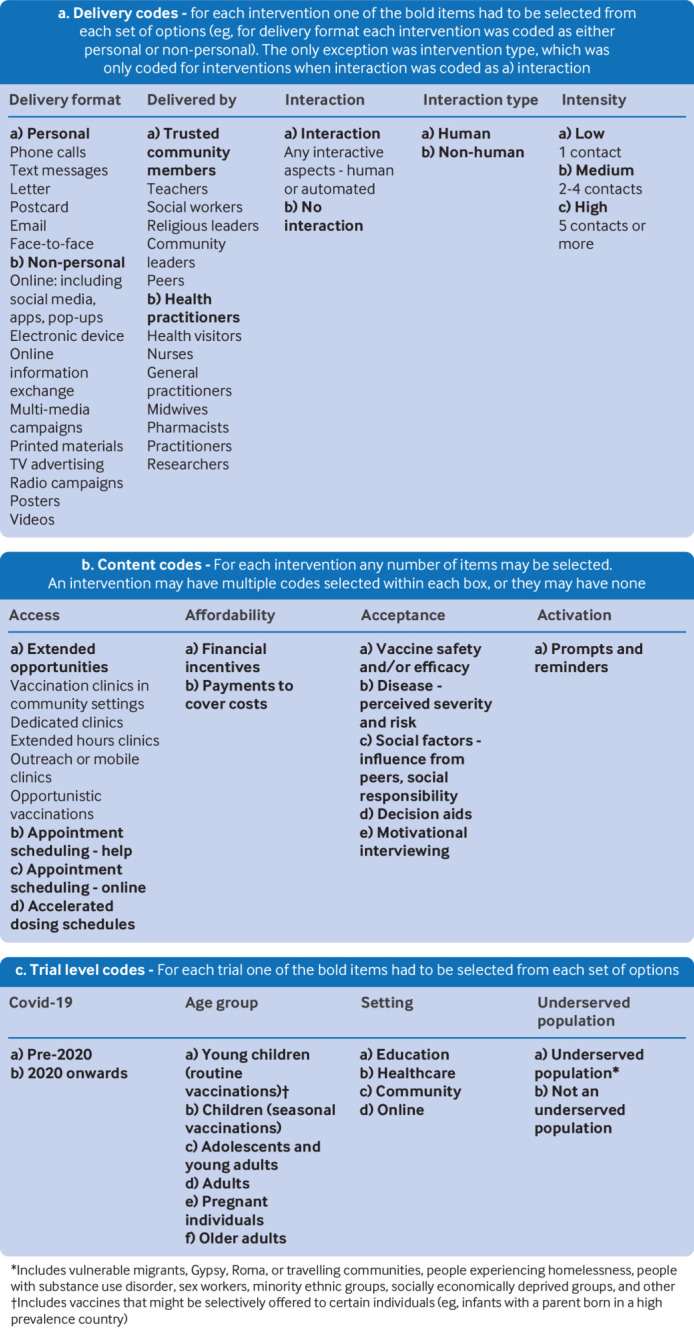
Coding framework used to systematically identify delivery and content components of interventions to increase vaccine uptake

Our component network meta-analysis was embedded in a comprehensive systematic review of 268 randomised controlled trials of interventions to increase vaccine uptake.^11^ The review was registered with PROSPERO (CRD42022369139) and the National Institute for Health and Care Research.^12^ Box 1 shows the eligibility criteria for the review, which are published in full elsewhere.^11^ The review was conducted in accordance with Cochrane methods,^13^ with risk of bias assessed at the outcome level for each study using the Cochrane risk of bias 2 tool.^14^ The 237 randomised controlled trials eligible for inclusion in the component network meta-analysis were those for which we had usable outcome data, and were able to code the intervention components using our bespoke coding framework (fig 1).

Box 1 **Eligibility criteria for systematic review**

**Study design**

Randomised controlled trials
Trials that randomised at least 100 participants*
Cluster randomised controlled trials with at least three clusters in each arm
Publication between January 2000 and April 2024
Conducted in high and upper middle income countries, as defined by the World Bank in July 2022†

**Participants**

Participants from all population groups living in the community and eligible for vaccination (or caregivers of those eligible for vaccination), including parents of young children, adolescents, and adults
Exclusions: studies targeting hospital inpatients, prisoners, and residents of care or nursing homes; studies focused on healthcare workers; and studies focused on specific clinical risk groups

**Vaccinations‡**

All routine and selective or targeted vaccinations on the UK immunisation schedule, including seasonal vaccinations

**Interventions**

Eligible interventions targeted the vaccine recipients or their caregivers aiming to increase demand for, or access to, vaccination
Exclusions: interventions aimed at the provider or healthcare system (eg, provider training or provider incentives); and those aimed at both the intended vaccine recipients and the providers or healthcare systems, unless effectiveness data were available for the component targeting the intended recipients of vaccines alone

**Comparators**

Eligible studies used comparator groups of no intervention, usual care, waitlist, attention placebo, or an alternative eligible intervention.

**Outcome**

Studies reporting data on vaccine uptake. Uptake could be reported as single vaccinations or completion of a full vaccination course, or both, documented in medical records or through self-report

*Restricted to large randomised controlled trials—the most robust evidence on intervention effectiveness—because of the substantial number of large primary studies conducted on this topic area.†Lower income countries were excluded as vaccine programmes differ between higher and lower income countries on several dimensions, including infrastructure, funding, accessibility, disease focus, and public trust.‡A large overlap exists between international (eg, WHO) and UK immunisation recommendations. Immunisations on the WHO schedule but not the UK schedule are those for diseases typically occurring in low income settings (eg, cholera). The only other exception was the varicella vaccine, which was not included on the UK immunisation schedule at the time of this review. Varicella was, however, included in this review as several high income countries routinely offer this vaccination to children.WHO=World Health Organization.

### Intervention level coding

To identify components for analysis, we developed a bespoke coding framework, enabling important intervention characteristics (including what is done and how it is done) to be systematically recorded for each intervention. Working with public contributors and professional stakeholders, we followed an iterative approach. Firstly, we identified two existing vaccination taxonomies from the literature and used them to pilot code several different broad intervention types, assessing whether the taxonomies could adequately capture key aspects of the interventions.^15^ ^16^ The 5As taxonomy (access, affordability, awareness, acceptance, and activation) for determinants of vaccine uptake was the most suitable, as it was able to capture important aspects of a wide range of interventions. This taxonomy was used as the foundation of our coding framework.^16^

We then extended and adapted the 5As through a multi-pronged approach. As we coded interventions using the taxonomy, we found several codes that were not applicable or were not possible to extract from the studies—for example, individual characteristics such as attitude to vaccines. We also highlighted emerging themes that were not captured by the 5As. These themes were documented and discussed with stakeholders, including behavioural scientists and immunisation specialists, before deciding to adapt the framework (see supplemental material A for an abridged version of the fieldwork diary we kept as a record of these changes). The resulting coding framework comprised codes for both content and delivery (see figure 1 for a summary of codes that were entered into the analysis model, and supplemental material B for our full coding framework with definitions).

Pairs of researchers (SRD, ZAT, EA, IA, or CEF) independently coded all constituent content and delivery components for each intervention arm, iteratively adapting the coding framework—especially in the early stages. Discrepancies were resolved through discussion, including with the wider project team as needed. Coding was conducted using all available information from the publications, with inquiries to the trialists when needed.

### Trial level codes

Trial features such as participant characteristics and setting could impact the effectiveness of the intervention (see initial logic model in supplemental material C). Each trial was coded for the trial level characteristics shown in the coding framework (see figure 1). We also extracted information on ethnicity, education, religion, and socioeconomic status. However, this information was incomplete and lacked consistency, so we could not meaningfully include it in our analyses.

### Statistical analyses

Our prespecified analysis plan is available.^17^ We used a bespoke component level meta-regression model based on a previously described model.^18^ The content, delivery, and trial level codes reported in figure 1 were incorporated in the model as indicator variables and covariates defined on two levels: trial and intervention. Each categorical variable was coded by a set of dummy binary variables, treating one category as the reference group. We also included one continuous variable describing the proportion of male participants in the trial. The model assumes additive effects of all indicators and includes an intercept to capture the effect, relative to control, of an intervention, the indicators of which are all equal to zero.

We assumed a binomial likelihood for the number of events and modelled relative intervention effects as log odds ratios. Based on the specification of the model described in supplemental material D (and elsewhere^18^), we defined all regression coefficients with reference to a control arm.

In our primary model we included random effects to capture variation in intervention effects between trials. We assumed a single between trial heterogeneity variance across the whole analysis, which corresponds to the amount of heterogeneity among trials with an identical set of intervention components in both arms, irrespective of what those components are. Correlations due to multi-arm trials were modelled by specifying a multivariate normal distribution for the relative treatment effects (both within and between trials). Supplemental material D provides further mathematical details of our model.

We implemented the models in a bayesian framework using the Markov chain Monte Carlo sampler JAGS,^19^ with non-informative prior distributions on all parameters. For all regression coefficients we specified a normal distribution centred on zero with a standard deviation of 100. For the heterogeneity standard deviation, we used a half normal distribution with a standard deviation of 2.5. To aid convergence, we centred all indicators about their mean.

#### Subgroup analyses

To explore how the effects of different intervention components vary, we conducted subgroup analyses informed by discussions with our stakeholders. These analyses investigated the effect of age group, social inequalities, and the covid-19 pandemic. For age group, we split the data into three categories: adults, adolescents (adolescents and young adults), and children. We explored the use of more granular categories (children categorised as young children - routine vaccinations and children - seasonal vaccination; adults categorised as adults, older adults, and pregnant individuals; and adolescents categorised according to whether the intervention targeted them or their parents or caregivers), but data were insufficient for the models to converge. To explore the impact of the covid-19 pandemic, we grouped trials into those conducted before 2020 and those conducted from 2020 onwards. Finally, to explore which components work best for the most vulnerable populations, we restricted the data to trials that specially targeted underserved populations. In each subgroup, we inspected the counts associated with each indicator and excluded from the model any indicator variable with no associated observations (ie, all interventions coded as zero or all coded as one).

#### Sensitivity analyses

To check the robustness of our results, we conducted a series of planned sensitivity analyses: a fixed effects analysis; excluding studies judged to be at high risk of bias; excluding studies with strongly outlying results; and investigating the effect of adjusting for clustering (see supplemental material E). We also conducted a post hoc sensitivity analysis to explore the robustness of our results excluding studies conducted in the US.

### Patient and public involvement

We engaged closely with a diverse group of 12 public contributors from underserved communities throughout this work, particularly in developing the coding framework. We held eight meetings during which we facilitated small group activities that had several tangible effects on the framework. For example, we asked participants working in three small groups to divide the list of all delivery formats extracted from studies into binary categories that could be used in our regression model. All three groups created almost identical categories, and we adopted these in our framework. Full details of our public involvement work, including the focus of the meetings, have been published elsewhere.^20^

## Results

We analysed data from 237 studies with a total of 4 361 717 participants. Table 1 provides a summary of the study characteristics (see supplemental material F for full details). Studies included between 100 and 964 870 participants.

**Table 1:** Characteristics of studies included in component network meta-analysis

Characteristics	No (%) of studies (n=237)
**Study type**	
Individually randomised controlled trials	181 (76.4)
Cluster randomised controlled trials	56 (23.6)
Country income level*	
High income:	222 (93.7)
Australia	10 (4.2)
Canada	3 (1.3)
European countries	38 (16.0)
Hong Kong Special Administrative Region, China	7 (3.0)
Israel	1 (0.4)
Japan	4 (1.7)
New Zealand	1 (0.4)
Singapore	2 (0.8)
Taiwan, China	1 (0.4)
USA	155 (65.4)
Upper middle income:	15 (6.3)
Brazil	1 (0.4)
China	12 (5.0)
Georgia	1 (0.4)
Guatemala	1 (0.4)
**Age group**	
Young children - routine vaccinations	38 (16.0)
Children - seasonal vaccinations	25 (10.5)
Adolescents and young adults	57 (24.0)
Adults	71 (30.0)
Pregnant individuals	11 (4.6)
Older adults	35 (14.8)
**Setting**	
Healthcare	156 (65.8)
Community or other	33 (13.9)
Education	25 (10.5)
Online	23 (9.7)
**Outcome**	
Series completion	62 (26.2)
Up-to-date vaccination	8 (3.4)
First dose	13 (5.4)
Any dose	154 (65.0)
**Time period†**	
Pre-2020	175 (73.8)
2020 onwards	62 (26.1)
**Underserved populations (n=63)**	
Socially economically deprived populations	18 (7.6)
Minority ethnic groups	12 (5.1)
Socially economically deprived, minority ethnic groups	14 (5.9)
People with substance use disorders	11 (4.6)
Vulnerable migrants	1 (0.4)
Minority ethnic groups and vulnerable migrants	1 (0.4)
People experiencing homelessness	1 (0.4)
Sex workers	1 (0.4)
Other underserved populations	4 (1.7)

* As defined by the World Bank, July 2022.

† In relation to the covid-19 pandemic.

Thirty eight studies were conducted on routine vaccinations aimed at young children, 25 on seasonal vaccinations for children (most, n=24, were on influenza vaccination), 57 at adolescents and young adults (n=47 on human papillomavirus vaccination), 71 at adults (n=24 on influenza vaccination, n=20 on covid-19), 11 at pregnant individuals (n=10 on influenza), and 35 at older adults (n=28 on influenza). Supplemental material H provides data on socioeconomic group, education, and religion.

The 237 studies included 570 arms, 362 of which were active intervention arms. The most common content components in active intervention arms were activation (prompts and reminders, 223 arms), vaccine safety and/or efficacy (155 arms), perceived disease risk (140 arms), social factors (80 arms), and extended opportunities (37 arms). Supplemental material section G provides full intervention coding by study.

Using the Cochrane risk of bias 2 tool, we judged 110 studies to be at low risk of bias, 96 to have some concerns, and 31 to be at high risk of bias, mainly due to issues with randomisation and measurement of the outcome (eg, self-reported vaccination status). See supplemental material I for summary plots of risk of bias judgements.

### Main analysis

**Fig 2:**
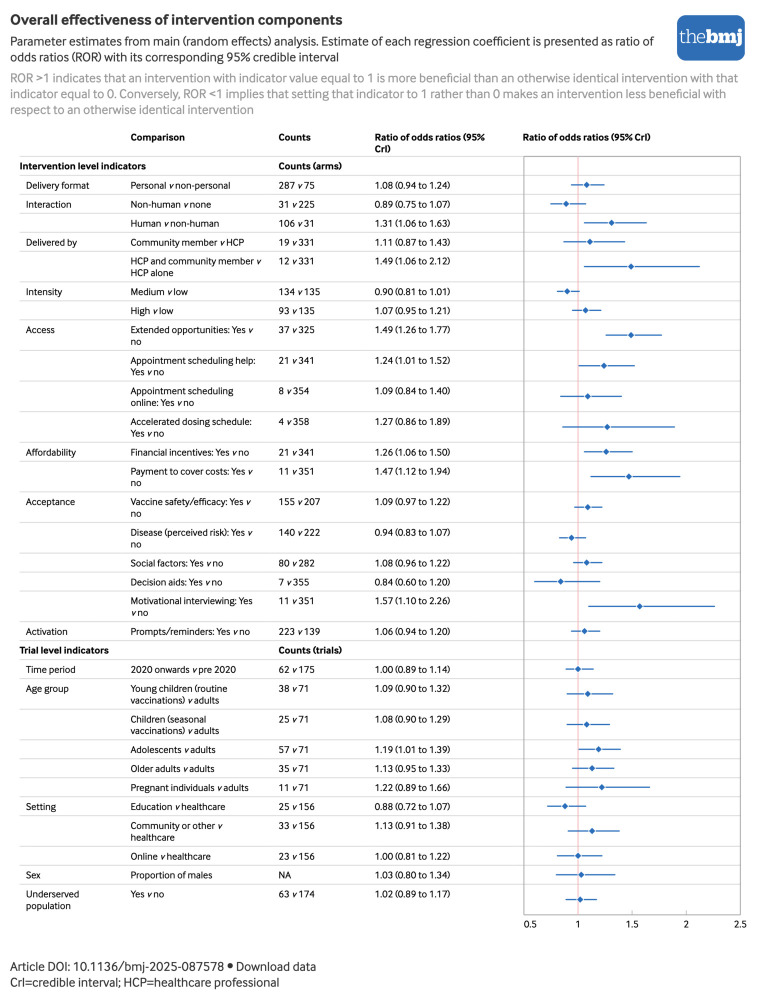
Parameter estimates from main (random effects) analysis. Estimates of each regression coefficient are presented as ratios of odds ratios with corresponding 95% CrI. An interactive version of this graphic is available at https://public.flourish.studio/visualisation/28205005/

**Fig 3:**
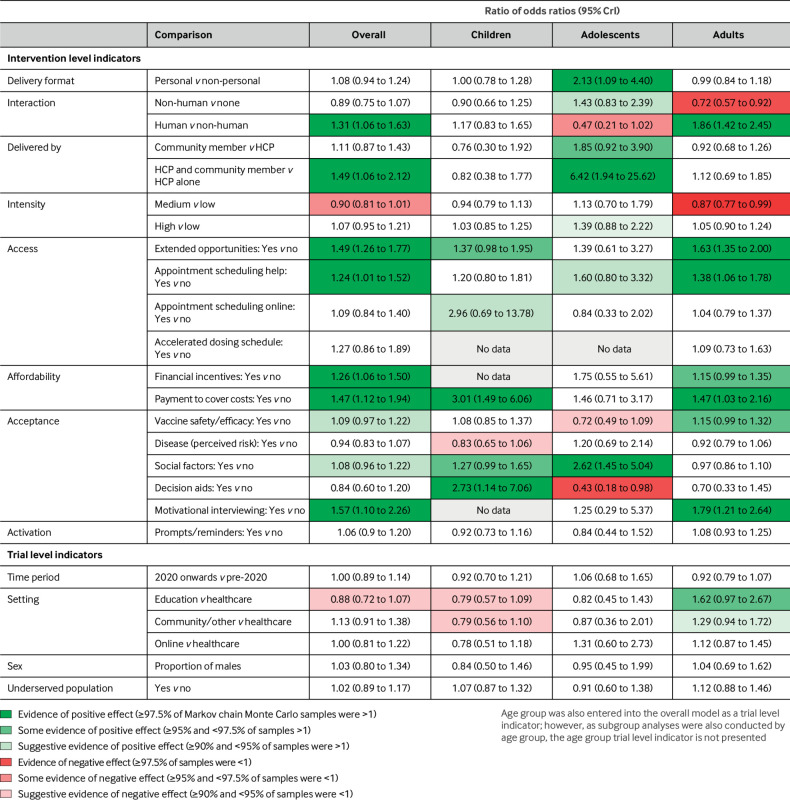
Results of main analysis and subgroup analyses by age group, expressed as ratios of odds ratios with 95% CrIs. Colour coding reflects the proportion of samples falling either side of 1 during Markov chain Monte Carlo sampling. CrI=credible interval; HCP=healthcare professional

**Fig 4:**
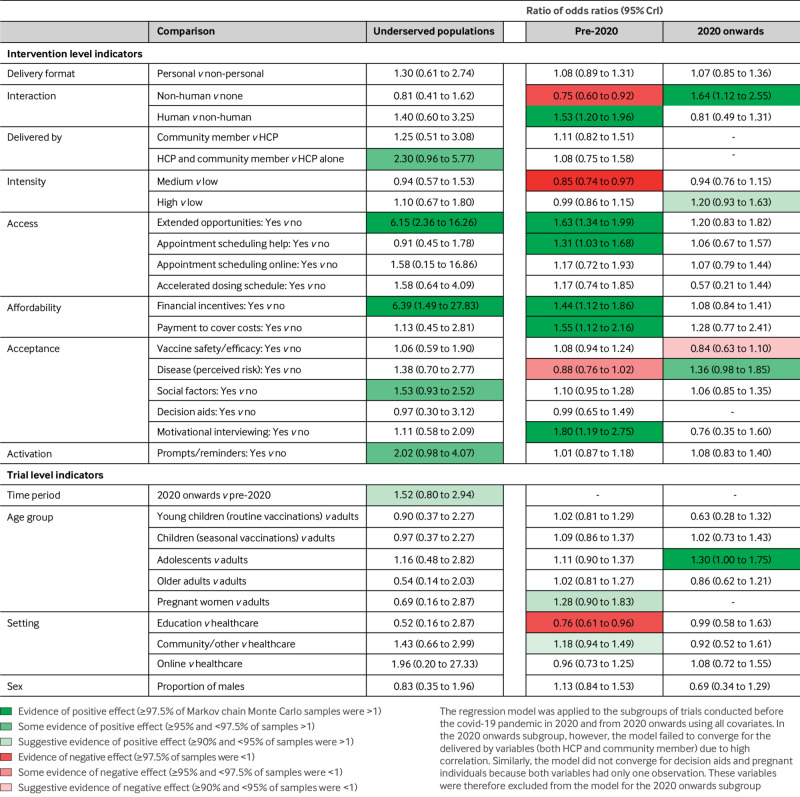
Results of subgroup analyses on covid-19 and underserved populations, expressed as ROR with 95% CrI. Colour coding reflects the proportion of samples falling either side of 1 during Markov chain Monte Carlo sampling. CrI=credible interval; HCP=healthcare professional; ROR=ratio of odds ratios

Figure 2 shows the forest plot for our primary analysis (random effects model). We present the estimate of each model coefficient as a ratio of odds ratios with the corresponding 95% credible interval (CrI). For a particular indicator, the ratio of odds ratios represents the additional effect (expressed as a ratio) of an intervention with indicator value 1 compared with an indicator value 0, conditional on the two interventions sharing the same values of all other indicators. Therefore, a ratio of odds ratios >1 indicates that an intervention with indicator value equal to 1 is more beneficial than an otherwise identical intervention with that indicator equal to 0. For example, in figure 2, appointment scheduling help has a ratio of odds ratios of 1.24 indicating that interventions that include appointment scheduling are more effective than interventions that do not but are otherwise identical. Conversely, a ratio of odds ratios <1 implies that setting that indicator to 1 rather than 0 makes the intervention less beneficial with respect to an otherwise identical intervention. Figure 3 and figure 4 present colour coded results where the colouring reflects the proportion of samples falling either side of 1 during the Markov chain Monte Carlo process. When ≥97.5% of Markov chain Monte Carlo samples were >1 we interpret this as evidence of a positive effect, ≥95% and <97.5% of samples >1 as some evidence, and ≥90% and <95% of samples >1 as suggestive evidence.

Figure 3 shows overall evidence of beneficial effects for the content components: extended opportunities (ratio of odds ratios 1.49, 95% Crl 1.26 to 1.77), appointment scheduling help (1.24, 1.01 to 1.52), financial incentives (1.26, 1.06 to 1.50), payments to cover costs (1.47, 1.12 to 1.94), and motivational interviewing (1.57, 1.10 to 2.26). For delivery components we observed evidence of beneficial effects for human interaction (1.31, 1.06 to 1.63), and delivery by community members alongside healthcare professionals (1.49, 1.06 to 2.12). There was also suggestive evidence of beneficial effects of providing information or education on vaccine safety and/or efficacy (1.09, 0.97 to 1.22), and social factors (1.08, 0.96 to 1.22). There was some evidence of a negative effect of medium intensity compared with low intensity interventions (0.90, 0.81 to 1.01). For trial level indicators, there was suggestive evidence that interventions delivered in educational settings were less effective that those delivered in healthcare (0.88, 0.72 to 1.07). Supplemental materials C provides a refined logic model incorporating our findings.

To aid interpretation of the component effects, we provide two examples to illustrate their impact on vaccine uptake. In a previous analysis of this dataset, we found that a typical education intervention was associated with an odds ratio of 1.33,^11^ which would increase an uptake rate of 80% to 84% (or from 20% to 25% in a low uptake population). Introducing extended opportunities to the intervention (ratio of odds ratios=1.49, fig 2) would alter this impact such that the intervention would increase an uptake rate of 80% to 89% (or 20% to 33% in a low uptake population). Alternatively, including instead a financial incentive as part of the intervention (ratio of odds ratios=1.26, fig 2) would alter the intervention’s impact such that it would increase an uptake rate of 80% to 87% (or from 20% to 30% in a low uptake population).

### Subgroup analyses

#### Age groups

See figure 3 for age group analyses and supplemental material J for corresponding forest plots. For parents or caregivers of children, no clear effects were seen for any of the delivery components. For adolescents, personal delivery formats showed evidence of beneficial effects. There was some evidence that delivery by community members alone and in combination with a healthcare professional are both more effective than delivery by a healthcare professional alone (although only two arms were coded as delivered by a community member alongside a healthcare professional). There was also suggestive evidence of a positive effect of high intensity interventions and those with a non-human interactive component compared with no interaction, and some evidence of a negative effect of human interaction (ie, human interactions are less beneficial than non-human interactions). For adults, the interaction components showed effects contradictory to those in adolescents. Interventions involving a non-human interactive element were less beneficial than identical interventions involving no interaction and human interaction was more beneficial than non-human interaction. Lastly, for adults, we found evidence of a negative effect of medium versus low intensity interventions.

For content components, we observed beneficial effects of extended opportunities in adults and some evidence of a positive effect in children, but not in adolescents. Appointment scheduling help had a beneficial effect in adults and there was suggestive evidence of an effect in adolescents. Financial incentives may be beneficial in adults but not in adolescents, and there were no data for children. Payments to cover costs were effective in children and in adults. Social factors had a beneficial effect in adolescents, and there was some evidence of an effect in children but not in adults. For decision aids the effects were contradictory, with beneficial effects shown in children but negative effects observed in adolescents. We found some evidence of a beneficial effect of information and/or education on vaccine safety and/or efficacy in adults, but suggestive evidence of a negative effect in adolescents. There was suggestive evidence that information on disease risk had a negative effect in children and no clear effect in adolescents and adults. Motivational interviewing showed evidence of a beneficial effect in adults but not in adolescents, and there were no data for children. Activation (prompts and reminders) had no clear effect in any of the age subgroups.

#### Underserved populations

For underserved populations we observed evidence of beneficial effects of extended opportunities and financial incentives (fig 4). We also found some evidence of a beneficial effect of delivery by both a community member and a healthcare professional, information and/or education about social factors, and activation (prompts and reminders).

#### Covid-19

Results for the pre-2020 period, before the covid-19 pandemic, were similar to the overall results, with evidence of beneficial effects for the components human interaction, extended opportunities, appointment scheduling help, financial incentives, payments to cover costs, and motivational interviewing. Non-human interaction and medium intensity interventions showed evidence of a negative effect, and providing information about the risk of disease showed some indication of a negative effect. Fewer trials contributed to the 2020 onwards period, with fewer effects observed. For two components we observed an opposite effect to that before 2020—both non-human interaction and perceived risk of disease showed beneficial effects from 2020 onwards compared with negative effects before 2020. There was also suggestive evidence of a negative effect of information or education on vaccine safety and/or efficacy (fig 4).

### Sensitivity analyses

For forest plots of sensitivity analyses, see supplemental material K. The results of the fixed effects model exhibited a much poorer fit to the data (a deviance information criterion of 2062 compared with 843 for the random effects model), providing evidence for heterogeneity between components. When we excluded studies at high risk of bias, the results were similar to those in the main analysis, with all components displaying the same direction of effects. After the removal of 13 studies that were strong outliers, a similar pattern of results was seen for most components, although the effects were mostly smaller, and more CrIs crossed the line of no effect. Findings from sensitivity analyses exploring assumed values for intraclass correlation coefficients showed a similar pattern of effects both without cluster adjustment and with large intraclass correlation coefficients. Findings from our post hoc sensitivity analysis to examine country context by excluding the 155 US based studies, indicated that some effects were no longer observed and some were more uncertain. This is partly due to the reduced number of studies (82 trials with 185 arms compared with 237 studies with 570 arms) and the resulting wider CrIs. However, positive effects of extended opportunities and payments to cover costs remained.

## Discussion

This component network meta-analysis investigated the comparative effectiveness of different content and delivery components of vaccine uptake interventions targeting recipients or their caregivers. Our analyses by age group explored which components work best for each group, allowing for targeted optimisation of age specific vaccination programmes.

For childhood vaccinations, interventions that target the barrier of affordability by providing payments to cover costs seemed to work, as did decision aids. There was some evidence that approaches to increase accessibility by extending opportunities for vaccination were effective. These strategies are likely to be particularly useful for busy parents, especially those with several children, for whom scheduling and attending appointments might be challenging even when they have no particular concerns about vaccination. Ensuring accurate real time recording of children’s immunisation status is also fundamental to effectively targeting interventions.^21^ Our findings support a recent UK Royal College of Paediatrics and Child Health commissioned report that recommends expanding service capacity, implementing flexible booking systems, and improving outreach initiatives as necessary solutions.^22^ We also found some evidence that providing information or education about social factors such as social responsibility might increase uptake. While the components vaccine safety and/or efficacy and risk of disease did not seem to be as important as other components in our analyses, wider literature highlights the prevalence of misinformation around vaccines and the importance of challenging it.^23^ ^24^ However, simple messaging about risks and benefits of vaccination may be insufficient.^25^

Among adolescents and young adults (where interventions could be aimed at the young person, their caregiver, or both) the negative effect of human interaction compared with non-human interaction supports some previous findings that dialogue based interventions are not as effective for adolescents as other groups.^26^ This highlights the need to co-develop effective methods of communication for this group (eg, using new interactive technologies). Building trust and long term confidence in immunisations among adolescents and young adults is important for creating positive attitudes towards immunisation that they can carry forward when making decisions for their own children. For content components, providing information about social factors such as social responsibility and peer support appeared particularly effective for adolescents, adding to the evidence base on the important role of social norms in vaccine uptake in adolescents.^27^ ^28^ The suggestive evidence of a negative effect of information about vaccine safety and/or efficacy was counterintuitive—a key focus of vaccination programmes for adolescents includes targeted education on vaccines in school settings.^29^ This finding should be interpreted cautiously and warrants further investigation.

For adults, targeting access barriers by providing extended opportunities for vaccination, help with scheduling appointments, and payments to cover vaccination costs had positive effects. These findings complement and extend those provided in two recent reviews that both reported that interventions to increase access and affordability were effective.^30^ ^31^ Providing information or education on vaccine safety and/or efficacy was effective as was motivational interviewing. Notably a recent review found comparable efficacy of motivational approaches and educational interventions, although there were issues with study quality and more research is needed.^32^ Adults also seemed to benefit from having human interaction as part of the interventions. These results align with a previous review of factors influencing vaccine uptake that found interactive interventions directed towards patients, including provider led educational initiatives, incentives, affordability efforts, or expansion of vaccination sites generally showed positive associations with vaccine uptake in older adults.^33^

In underserved communities, we observed a beneficial effect of making vaccines more accessible by extending vaccination opportunities and providing financial incentives. These components likely mitigate multiple barriers to vaccine uptake that are especially salient in these populations, including obstacles related to healthcare service delivery and out-of-pocket expenses.^34^ Further multidisciplinary research is, however, needed to better understand the moral and ethical implications of providing financial incentives, including careful consideration of potential harms and use within local contexts. The public contributors proposed that incentives could be a short term solution that ultimately might lead to more distrust in the long term, and they highlighted that the focus should be on longer term solutions that build trust and confidence in vaccination. Providing information on social factors may also be beneficial, supporting previous reports that social norms are particularly important in vaccine uptake among some migrant populations.^35^ In our analyses, the underserved populations was the only subgroup where some evidence of a beneficial effect of activation (prompts and reminders) was observed. This is surprising given that in reviews of broad categories of interventions, reminders have been shown to be effective at increasing vaccine uptake.^36^ Our findings suggest that within interventions categorised broadly as reminders, the reported beneficial effects could potentially have been attributable to other components of the intervention (eg, educational), which we were uniquely able to account for here. The evidence of some beneficial effect of the involvement of community members alongside healthcare professionals in delivering interventions for these groups shows the importance of working in partnership with local community organisations to deliver interventions that are tailored to the diverse needs of local communities.^37^ The public contributors emphasised that it is important for both community members and healthcare professionals to support people to get vaccinated.

The effectiveness of certain components may have been influenced by the covid-19 pandemic. Although financial incentives showed positive effects before 2020, we did not observe similar effects from 2020 onwards, indicating a potential change in public response during the pandemic. Interestingly, although we found no clear effect of providing information or education on perceived disease risk in our overall analysis, we found some evidence of a negative effect before 2020, with vaccine uptake being lower when information on disease risk was provided, but some evidence of a beneficial effect in our 2020 onwards subgroup. This pattern potentially aligns with predictions of protection motivation theory, which suggests that health behaviours are influenced by an individual’s perceived level of threat. During the covid-19 pandemic, heightened perceptions of threat could have extended to other vaccines and health protective behaviours amplifying the impact of information on disease risk.^38^

### Strengths and limitations of this study

Strengths of our analysis include the large dataset consisting of 237 studies, allowing us to conduct a component level analysis. Studies covered a wide range of intervention and vaccine types making our findings widely generalisable. We used complex analyses, novel to this topic, to assess the effectiveness of specific delivery and content components of interventions. Our bespoke coding framework incorporated input from important stakeholders, including members of underserved communities and immunisation specialists, ensuring we coded components of interventions that were important to those involved in the development and delivery of vaccine programmes as well as recipients of vaccine programmes.

Our findings should also be considered in the context of several limitations. Because of the large amount of evidence in this area, we included only randomised controlled trials with ≥100 participants because these studies represent the best available evidence; however, it is possible that some small randomised controlled trials would have been eligible for inclusion. Similarly, restricting our review to randomised controlled trials may have led to some evidence—particularly for some types of interventions (eg, attempts to challenge misinformation) or population groups—being excluded. In studies aimed at general populations, there were important shortcomings in terms of quality and completeness of reporting of population characteristics that are known to be associated with inequalities in vaccination uptake, including ethnicity and markers of socioeconomic status.^39^ We had planned to conduct subgroup analyses on these; however, only a small percentage (12%, n=28/237) of primary study reports provided subgroup analyses (10% (n=23/237) by ethnicity or race, 3% (n=8/237) by socioeconomic information, and 1.7% (n=4/237) by education) so it was not possible. However, we did assess the effectiveness of interventions aimed specifically at underserved populations. Despite the large dataset overall, the evidence base is smaller, and therefore more uncertain, when focusing on specific intervention components and population groups. We had hoped to conduct subgroup analyses for separate underserved populations, although it was not possible to disaggregate further owing to insufficient data on distinct underserved groups. We also planned subgroup analyses that further subdivided the children and adult groups; but again, this was not possible. Likewise, some of the components were removed from some models because of no evidence. Although it is important to adjust for trial level indicators in the models, the trial level effects themselves should be interpreted with some caution. For example, the finding of no evidence of a difference between healthcare and educational settings among adolescents should not be taken to mean that delivering interventions for adolescents in educational settings is not beneficial. The delivery of vaccinations in educational settings can play an important role in maximising vaccine uptake and reducing inequalities compared with healthcare settings in this age group.^40^ Meanwhile, although delivery in an educational setting showed some beneficial effect in adults, this finding was based on a small number of studies (eg, those targeting both staff and students in university settings), so should not be considered more widely generalisable.

Our coding framework was developed specifically to capture important delivery and content components; however, the coding process itself is subjective and depends upon the extent of information provided in reports of studies. Components may have varied between studies in terms of their content and the extent of implementation, and aspects of interventions may have been missed owing to a lack of in-depth reporting of procedures. Furthermore, some contextual factors (eg, at the country level) could not be fully accounted for in the analyses. For example, different countries can have noticeably different financial protection models for healthcare access. Two thirds of studies (155 out of 237) were conducted in the US. To assess whether the observed effects could have been driven by interventions applied in that specific country context, we conducted a post hoc sensitivity analysis including only studies conducted outside of the US. Effect estimates overall were more uncertain due to the smaller sample; however, extended opportunities and payments to cover costs remained effective. We could not include a small proportion of studies from the systematic review in our component network meta-analysis due to lack of outcome data or the component coding being identical across arms. Although we cannot be sure what impact these studies would have had on the component analysis, it is not likely to have been substantial, given the small proportion of studies.

No validated tool exists with which to assess certainty in the evidence for component network meta-analysis. Using the CINeMA (Confidence in Network Meta-Analysis) framework, however, we judged certainty to be moderate for network meta-analysis comparisons of broad intervention groupings versus control.^11^

### Conclusions

Our findings have important implications for the development, optimisation, and implementation of interventions to increase vaccine uptake, providing nuanced insights into which components work best in different population groups and contexts. The findings should be integrated with local level insights to guide the implementation of combinations of intervention components that are effective for local populations. Consideration of the available economic data on intervention costs and cost effectiveness will further inform decision making around which interventions to implement given limited resources.

**What is already known on this topic**

A range of broad intervention types (eg, improving access and affordability) have proven effective in increasing vaccine uptake; however, they are often complex and involve multiple components
Identifying which intervention components are effective is key to inform the development, optimisation, and implementation of interventions to increase vaccine uptake
No existing reviews assess the effectiveness of individual components using a component network meta-analysis approach across different populations and contexts

**What this study adds**

In this component network meta-analysis, effective intervention content components for increasing vaccine uptake overall were extended opportunities, appointment scheduling help, financial incentives, payments to cover costs, and motivational interviewing
Effective intervention delivery components overall were human interaction and delivery by community members alongside healthcare professionals
Effective components varied by age group, for underserved populations, and in analyses investigating the impact of the covid-19 pandemic

## Data Availability

All data and codes for analysis are available on the GitHub repository (https://github.com/AnnieDavies/VaccineReview ).

## Web extra

Extra material supplied by authors

## Notes

Contributors: CEF, JPTH, DMC, LY, LL, and JY conceived and designed the study. SD developed and ran all searches. SRD, ZAT, IA, EA, and CEF selected the articles and extracted data. SRD, TP, ZAT, EA, and CEF completed risk of bias assessments. CM led on public involvement. ZAT created and maintained the Access database. SRD, CEF, LY, LL, JY, JPTH, DMC, and CM developed the coding framework together with the public contributors. ALD conducted the statistical analyses with input from JPTH, DMC, CEF, and SRD. CEF, SRD, ALD, JPTH, DMC, LL, LY, JY, SI, and HC interpreted the results. SRD wrote the first draft of the manuscript. All authors provided comments and critical revisions on drafts of the manuscript. All authors had full access to all the data in the study and had final responsibility for the decision to submit for publication. SRD is the guarantor. The corresponding author attests that all listed authors meet authorship criteria and that no others meeting the criteria have been omitted.

Transparency: The lead author (SRD) affirms that this manuscript is an honest, accurate, and transparent account of the study being reported; that no important aspects of the study have been omitted; and that any discrepancies from the study as originally planned have been explained.

Dissemination to participants and related patient and public communities: The findings of this study have been shared with members of the patient and public involvement group, who continue to support the dissemination of the results to local community groups. The study findings have been shared, and will continue to be shared, through presentations at meetings and conferences. The results will also be communicated through media channels such as the NIHR Health Protection Unit in Evaluation and Behavioural Science website and social media channels.

Provenance and peer review: Not commissioned; externally peer reviewed.
