# Supplementary material for: Effectiveness of interventions to increase vaccine uptake: component network meta-analysis
Source: BMJ. 2026 Apr 15;393:e087578. doi: 10.1136/bmj-2025-087578 (PMC13081225; doi:10.1136/bmj-2025-087578)
Supplement: Supplementary file 1 — Supplementary information: Supplemental material A-M [file davs087578.ww.pdf]

Supplemental material: Effectiveness of interventions to increase vaccine uptake: component network meta-analysis

Contents

A: Abridged fieldwork diary showing adaptations to coding framework 2
B: Coding framework 3
C: Initial and refined logic models 5
D. Mathematical details 6
E: Cluster adjustments 6
F: Study characteristics 8
G: Intervention coding for all studies 19
H: Socio-economic data from studies 40
I: Risk of bias summary plots 63
Risk of bias summary plots for cluster RCTs. 72
J: Subgroup analyses 73
Age group: Adults 73
Age group: Adolescents/young adults 73
Age group: Children 74
Underserved populations 74
Time period: Pre-2020 75
Time period: 2020 onwards 75
K: Sensitivity analyses 76
Sensitivity analysis: Fixed effects 76
Sensitivity analysis: Removal of studies at high risk of bias 76
Sensitivity analysis: Removal of outliers 77
Sensitivity analysis: Cluster adjustments 78
Post-hoc sensitivity analysis: Removal of studies conducted in the United States 79
L: Deviations from analysis plan 79
M: References to included studies 79

A: Abridged fieldwork diary showing adaptations to coding framework

**Code**	**Issues to address**	**Suggested changes/elaborations to the code or definition.**
Access	Quite a lot of studies include appointments being made for the individual which could potentially improve access to vaccination. Additionally, some studies included online scheduling of appointments which likewise may improve access by making scheduling easier.<br><br>Discussion with stakeholders at UK Health Security Agency (UKHSA) suggested these would be interesting components to look at.	Add as a subcodes of access:<br><br>**- Appointment scheduling – help**<br>**- Appointment scheduling - online**
Affordability	In discussions with the public contributor group they highlighted that the affordability code includes both financial incentives and payment to cover costs. They suggested that these should be two separate codes as they might see incentives differently to payments to cover costs.	Create two subcodes for affordability:<br><br>- **Financial incentives**<br>**- Payments to cover costs**
Interaction	We need some way to capture human dialogue within the interventions. Some interventions are one-way, others have automated interaction and another group have two-way human interaction. The public contributor group highlighted the importance of human interaction within vaccination.	Create three codes:<br><br>**- Interaction: None**<br>**- Interaction: Any**<br>**- Interaction: Human**
Frequency/intensity of interventions	The interventions vary quite dramatically in frequency/intensity of features. The initial coding framework did not capture this. For example, for reminder letters the frequency can be quite different (e.g. 1 versus 7). Meanwhile, for education interventions there could be for example a one-off 30 min session vs. multiple sessions over several weeks or months. We discussed how to capture this within the project team including immunisation experts from UKHSA, and the public contributor group.	Create the code:<br><br>**- Contact (we count the number of 'contacts' the recipient has regarding vaccination, this could be no. of reminders, no. of leaflets and reminders etc.)**

B: Coding framework

Delivery codes		
**Component**	**Component options**	**Definition of component options**
Delivery format	Personal	Any delivery format that is directed to a person, for example: phone calls, text messages, letters, postcards, emails, or face-to-face contact.
	Non-personal	Any delivery format that is not directed personally to an individual, for example: multi-media campaigns, printed materials, TV advertising, radio campaigns, posters, videos.
Interaction	Interaction	Any interaction within the intervention. This could be automated without human interaction, for example, automated text messages sent in response to a certain selection made by the participant: 'select 1 if you want more information on side effects, select 2 if you want more information on….' and the reply is standard/scripted for everyone such as a link to a webpage on vaccine side effects. Human dialogue refers to any unscripted dialogue with a human (e.g. face to face, telephone, or via social media).
	None	The intervention includes no interactive elements.
Human interaction	Human interaction	Any human dialogue (e.g. face to face, via telephone, or social media).
	Non-human interaction	Automated interactive elements that do not include direct human involvement. This could be interactive texts, for example.
Delivered by	Healthcare professional	A healthcare professional was involved in at least some part of delivering the intervention.
	Community member	A community member was involved in at least some part of delivering the intervention.
Intensity	High	'Number of contacts' was used as a marker of intervention intensity. A contact might be for example, a letter, a phone call, an email, an education session, or a reminder. We counted the maximum possible number of contacts (e.g. where 'up to 3 reminders were sent' we coded as 3; where reminder, followed by phone call (if needed), followed by home visit (if needed) we counted 3 contacts). The counts were then grouped into high, medium and low with high intensity referring to five or more contacts.
	Medium	Two to four contacts.
	Low	One contact.
Intervention content codes		
Access	Extended opportunities	Extending access can refer to vaccines being provided at different locations, different times (e.g. weekends), extended times (e.g. early mornings and evenings), opportunistic vaccinations, and walk-in vaccinations.
	Appointment scheduling help	Help in scheduling an appointment, either face to face, or via the telephone.
	Appointment scheduling online	The ability to book an appointment online, for example, through an appointment link sent to participants, or via an app.
	Accelerated dosing schedule	Where vaccination dosing schedules are accelerated (e.g. doses at 1,2,3 months rather than 1,3,6 months).

Affordability	Financial incentives	Incentives offered in exchange for getting vaccinated.
	Payment to cover costs	Any payment to cover costs of attending vaccination for example vouchers for taxi costs, bus tickets etc.
Awareness*	Awareness*	Providing information to intervention recipients on vaccine schedules and recommendations.
Acceptance	Vaccine safety and/or efficacy	Providing information on vaccine safety/side effects and efficacy of vaccines.
	Disease perceived risk	Providing information about the disease that the vaccine protects against. What are the perceived risks? What is the severity of the disease?
	Social factors	Information regarding social influence. Any information regarding peer support/ input/ recommendations. Information about social responsibility.
	Decision aids	Any intervention that incorporates a decision aid. Decision aids provide participants with evidence-based information on the options available, along with likely outcomes, benefits, harms and uncertainties.
	Motivational interviewing	Any intervention that includes motivational interviewing. Motivational interviewing focuses on exploring and resolving ambivalence and centres on motivational processes within the individual that facilitate change.
	Alternative provisions*	Alternative forms of vaccinations (e.g. injections, formulations)<br>• Alternative settings<br>• Alternative vaccine providers (e.g. doctor administering vaccine instead of nurse
Activation	Prompts and reminders	Reminders include both an initial invitation to be vaccinated when a routine vaccination becomes due as well as any additional reminders/recall before the vaccine is due or once it becomes overdue.
	Mandatory policies*	Workplace policies and practices.<br>Entry to childcare settings/ schools blocked in the absence of proof of vaccination status.

Footnote: Codes marked with an * were included in our framework, but were not entered into our analyses. This was due to too little evidence (alternative provisions, mandatory policies) or because of the large proportion of studies assigned the code and it being highly correlated with acceptance codes meaning that it could not be meaningfully interpreted (awareness).

C: Initial and refined logic models

Initial logic model: We developed a logic model prior to conducting our review to identify key variables that may impact on intervention effectiveness such as contextual factors and intervention features. This model was used to guide the main and subgroup analyses.

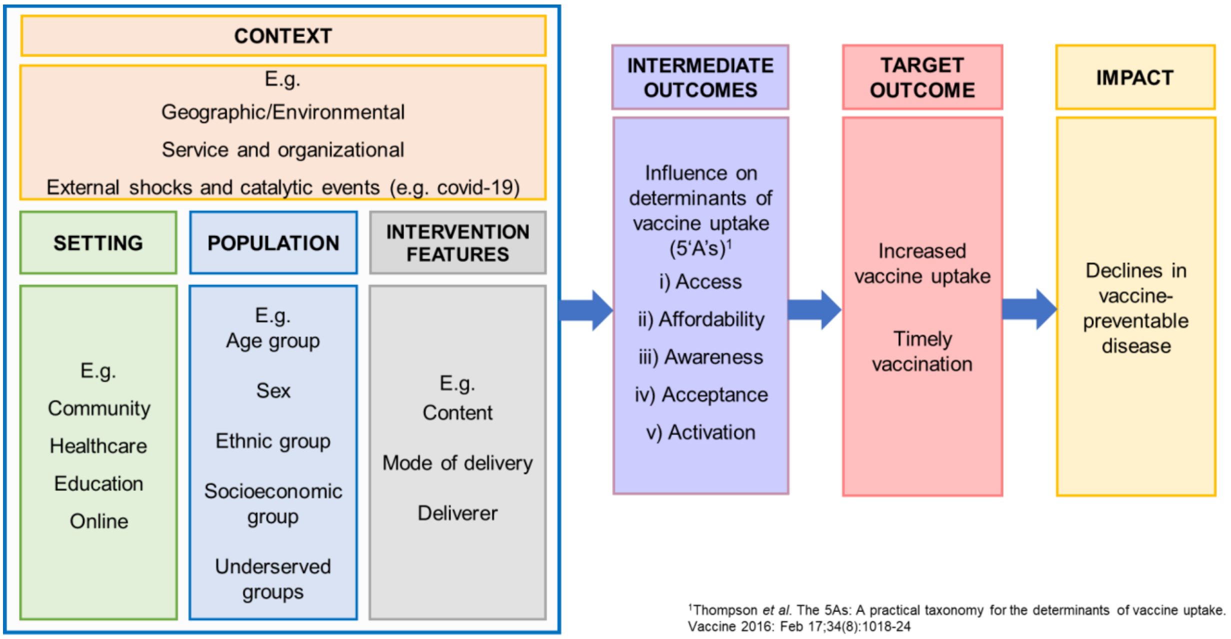

Refined logic model: We refined the model incorporating evidence from the overall analysis presented in this review.

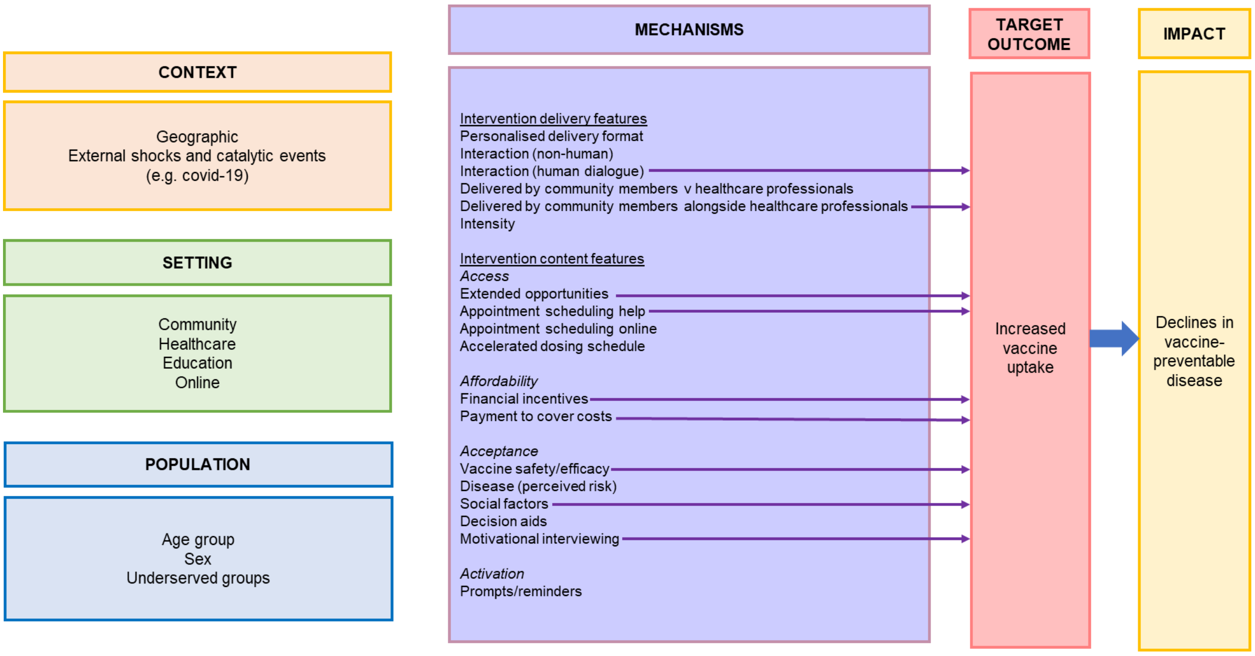

Footnotes: Refined model shows concepts that could be coded and incorporated into the analyses based on information provided in primary study reports. Other concepts are likely to be important but could not be coded (e.g. ethnicity). Purple arrows show intervention features found to be associated with vaccine uptake overall but effects differ by context/population group (see Figures 3 and 4 in the main manuscript).

D. Mathematical details

We consider a model of N trials, $i = 1, \ldots, N$. Each trial i is associated with A_i arms labelled $k = 1, \ldots, A_i$. The intervention in arm k of trial i is labelled $t_{i,k}$. We write $n_{i,k}$ for the number of participants (sample size) in this arm and $r_{i,k}$ for the number of events. We model the number of events as following a binomial distribution,

$$r_{i,k} \sim \mathrm{Bin}(n_{i,k}, p_{i,k}).$$

We define $\delta_{i,1k}$ as the trial specific relative treatment effect between $t_{i,1}$ and $t_{i,k}$ in trial i measured as a log odds ratio (LOR),

$$\mathrm{logit}(p_{i,k}) = \mu_i + \delta_{i,1k}$$

$$\mu_i = \mathrm{logit}(p_{i,1}).$$

The reference arm $t_{i,1}$ is either a control arm (C) or another active intervention (A_i). For the random effects model, we assume the trial specific effects follow a multivariate normal distribution

$$\boldsymbol{\delta_i} \sim N(\boldsymbol{\theta_i}, \boldsymbol{\Sigma_i})$$

centred on the mean effects $\boldsymbol{\theta_i}$, with between trial covariance matrix $\boldsymbol{\Sigma_i}$. The covariance matrix depends on heterogeneity variance, τ^2, which we assume captures the between-trial variance for all arms. The diagonal elements of $\boldsymbol{\Sigma_i}$ are equal to τ^2 and the off diagonal elements are $\tau^2/2$. For the fixed effect model we set $\tau^2 = 0$.

Each intervention in each trial is associated with a set of n intervention covariates. Control arms, defined as `no active intervention', are not coded according to the analytic framework and therefore, have no associated intervention covariates. We label the intervention covariates as $x_{i,jk}$, $j = 1, \ldots, n$ where $k = 1, \ldots, A_{i-1}$ when the reference arm is a control and $k = 1, \ldots, A_i$ otherwise. As well as factors associated with each individual intervention, we also include p study-level covariates, $z_{i,j}$, $j = 1, \ldots, p$. In a given trial, the study-level covariates are common to all arms and therefore do not depend on the intervention k.

We define a meta-regression model on the mean relative effects in each trial, $\theta_{i,1k}$. The model takes a different form depending on whether the reference arm is a control arm (control comparison) or another active intervention (active comparison). Assuming transitivity between relative effects, the model for active comparison trials is constructed by taking the difference between two control comparison models in different intervention arms of the same trial. Terms that do not depend on the intervention cancel out in this subtraction, meaning the active comparison model does not include an intercept or trial-level indicators. The model is summarized as follows,

$$\theta_{i,1k} = \begin{cases} \alpha + \sum_{j=1}^{n} \beta_j x_{i,jk} + \sum_{j=1}^{p} \gamma_j z_{i,j} & \text{for } t_{i,1} = C \\ \sum_{j=1}^{n} \beta_j (x_{i,jk} - x_{i,j1}) & \text{for } t_{i,1} = A_i \end{cases}$$

where the intercept α is the effect, relative to control, of an intervention whose covariates are all set to 0. The parameters $\beta = (\beta_1, \ldots, \beta_n)^\top$ and $\gamma = (\gamma_1, \ldots, \gamma_p)^\top$ are the regression coefficients for the intervention and study-level indicators respectively. Based on this construction, all regression coefficients are defined with reference to a control arm.

E: Cluster adjustments

As described in Davies et al, we accounted for clustering in CRCTs by adjusting the number of events and participants per arm by the design effect. Where possible, we specified the design effect in terms of the intraclass correlation coefficients (ICCs) reported in each trial. For studies that did not report an ICC we used an external estimate based on other CRCTs in this review (0.7 for trials clustered at the household level and 0.05 otherwise). Based on available ICCs in our dataset, we performed sensitivity analyses without cluster adjustments (ICC=0) and with larger ‘conservative’ ICC values (1 for households and 0.3 otherwise). For details of our cluster adjustment methods we refer to Davies et al and the appendices therein.

Davies S, Davies, AL., Higgins, JPT, Caldwell, DM., Thornton, ZA., Aiton, E, Ali, I, Dawson, S, McGrath, C, Parkhouse, T, Yardley, L, Yates, J, Letley, L, Ismail, S, French, CE. Recipient-focused interventions to increase vaccine uptake in high and upper-middle income countries: a systematic review and network meta-analysis *eClinicalMedicine* 2025; **90**.

F: Study characteristics

	Study	Design	N	N clusters	Outcome	Age	Vaccine Type	Covid-19	Setting	% male	% female	% white	Country	RoB
**1**	**Abroms 2023**	RCT	719	NA	Any	Adults	Covid-19	Post-	Online	0.26	0.74	0.80	United States	Some concerns
**2**	**Alonge 2023**	RCT	4296	NA	Any	Adolescents	Covid-19	Post-	Healthcare	0.51	0.49	0.78	United States	Low
**3**	**Anraad 2023**	RCT	1236	NA	Any	Pregnant individuals	Childhood Vaccines	Post-	Online	0.00	1.0	NA	Netherlands	Some concerns
**4**	**Arnold 2022**	RCT	506	NA	Any	Adults	BCG Tuberculosis, Childhood Vaccines, Hepatitis A, Hepatitis B, Influenza	Pre-	Community/Other	0.93	0.07	NA	Germany	Some concerns
**5**	**Arthur 2002**	Cluster RCT	2052	NA	Any	Older adults	Influenza	Pre-	Community/Other	0.39	0.61	NA	United Kingdom	Some concerns
**6**	**Bartos 2022**	RCT	2101	NA	Any	Adults	Covid-19	Post-	Online	0.50	0.50	1.00	Czech Republic	Some concerns
**7**	**Bartu 2006**	RCT	152	NA	Any	Young children	Childhood Vaccines	Pre-	Community/Other	0.00	1.0	0.89	Australia	High
**8**	**Baskin 2018**	RCT	30748	NA	Any	Adults	Influenza	Pre-	Education	0.50	0.50	NA	United States	Low
**9**	**Bastani 2022**	RCT	238	NA	Series	Adolescents	HPV	Pre-	Healthcare	0.07	0.93	0.02	United States	High
**10**	**Bennett 2015**	RCT	661	NA	Series	Adults	HPV	Pre-	Education	0.00	1.0	0.67	United States	Some concerns
**11**	**Berg 2008**	Cluster RCT	NA	134791	Any	Older adults	Influenza	Pre-	Healthcare	0.50	0.50	NA	United States	High
**12**	**Berg 2004**	Cluster RCT	181590	181590	Any	Adults	Influenza	Pre-	Healthcare	0.46	0.54	NA	United States	High
**13**	**Berkhout 2018**	Cluster RCT	10597	75	Any	Older adults	Influenza	Pre-	Healthcare	0.42	0.58	NA	France	High
**14**	**Bernard-Genest 2021**	RCT	130	NA	Any	Adults	HPV	Post-	Healthcare	0.00	1.0	NA	Canada	Low
**15**	**Berset 2022**	RCT	945	NA	Any	Children	Covid-19	Post-	Healthcare	0.48	0.52	0.24	United States	Low
**16**	**Berset 2023**	RCT	1312	NA	Any	Young children	Childhood Vaccines	Post-	Healthcare	0.54	0.48	0.15	United States	Low
**17**	**Bethke 2024**	Cluster RCT	6512	25	Any	Young children	Childhood Vaccines	Pre-	Education	0.54	0.46	NA	Germany	Low
**18**	**Bian 2023**	Cluster RCT	375	202	Any	Older adults	Covid-19	Post-	Education	0.47	0.53	NA	China*	Some concerns

	Study	Design	N	N clusters	Outcome	Age	Vaccine Type	Covid-19	Setting	% male	% female	% white	Country	RoB
19	**Borg 2018**	Cluster RCT	6619	5534	Any	Children	Influenza	Pre-	Healthcare	0.51	0.49	NA	Australia	Low
20	**Bourgeois 2008**	Cluster RCT	144	8	Any	Adults	Influenza	Pre-	Community/Other	0.51	0.49	NA	United States	Some concerns
21	**Bowman, 2014**	RCT	460	NA	Series	Adults	Hepatitis B	Pre-	Community/Other	0.50	0.50	NA	United States	Some concerns
22	**Brigham 2012**	RCT	424	NA	Any	Adolescents	Childhood Vaccines, MenACWY	Pre-	Healthcare	0.45	0.55	0.13	United States	Low
23	**Bronchetti 2015**	RCT	9358	NA	Any	Adults	Influenza	Pre-	Education	0.43	0.57	NA	United States	Some concerns
24	**Burkhardt 2023**	RCT	1235	NA	Any	Adolescents	Covid-19	Post-	Healthcare	0.52	0.48	0.16	United States	Low
25	**Buttenheim 2022**	RCT	11188	NA	Any	Adults	Influenza	Post-	Healthcare	0.45	0.55	0.69	United States	Low
26	**Campos-Mercade 2021**	RCT	9560	NA	Any	Adults	Covid-19	Post-	Online	0.50	0.50	NA	Sweden	Low
27	**Cataldi 2024**	Cluster RCT	49403	8	Series	Adolescents	HPV	Post-	Community/Other	0.51	0.49	0.67	United States	Low
28	**CentersforDisease 2012**	RCT	878	NA	Series	Young children	Childhood Vaccines	Pre-	Healthcare	0.53	0.47	NA	United States	Low
29	**Chai 2013**	RCT	1992	NA	Any	Adults	Influenza	Pre-	Healthcare	0.48	0.52	NA	China*	Some concerns
30	**Chang 2023**	RCT	57893	NA	Any	Adults	Covid-19	Post-	Healthcare	0.42	0.58	0.21	United States	Low
31	**Chao 2015**	RCT	12255	NA	Series	Adolescents	HPV	Pre-	Healthcare	0.00	1.0	0.24	United States	Low
32	**Chodick 2021**	RCT	21592	NA	Any	Adolescents	HPV	Pre-	Online	0.00	1.0	NA	Israel	Low
33	**Clayton 2021a**	Cluster RCT	678	678	Series	Young children	Childhood Vaccines	Pre-	Online	0.14	0.86	0.97	United States	Some concerns
33	**Clayton 2021b**	Cluster RCT	815	808	Series	Young children	Childhood Vaccines	Pre-	Healthcare	0.50	0.50	NA	United States	Some concerns
34	**Coley 2018**	RCT	303965	NA	Series	Adolescents	HPV	Pre-	Healthcare	0.50	0.50	NA	United States	Low
35	**Conner 2017**	RCT	13806	NA	Any	Older adults	Influenza	Pre-	Healthcare	0.44	0.56	0.97	United Kingdom	Low
36	**Cox 2012**	RCT	1175	NA	First	Adults	Hepatitis B	Pre-	Healthcare	0.61	0.39	0.12	United States	Low
37	**Cutrona 2018**	RCT	30000	NA	Any	Adults	Influenza	Pre-	Healthcare	0.41	0.59	0.78	United States	Low
38	**Dai 2021a**	RCT	93354	NA	Any	Adults	Covid-19	Post-	Healthcare	0.43	0.57	NA	United States	Low
38	**Dai 2021b**	RCT	67092	NA	Any	Adults	Covid-19	Post-	Healthcare	0.44	0.56	NA	United States	Low
39	**Dalby 2000**	RCT	142	NA	Any	Older adults	Influenza	Pre-	Community/Other	0.33	0.67	NA	Canada	Low
40	**Daley 2014**	Cluster RCT	NA	16	Any	Adolescents	Childhood Vaccines	Pre-	Education	0.50	0.50	NA	United States	High
41	**Daley 2002**	RCT	1234	NA	Any	Young children	Childhood Vaccines	Pre-	Healthcare	0.53	0.47	NA	United States	High

	Study	Design	N	N clusters	Outcome	Age	Vaccine Type	Covid-19	Setting	% male	% female	% white	Country	RoB
42	**Daniels 2007**	RCT	186	NA	Any	Older adults	Influenza	Pre-	Community/Other	0.25	0.75	0.08	United States	High
43	**Dapp 2011**	RCT	2580	NA	Any	Older adults	Influenza	Pre-	Healthcare	0.37	0.63	1.00	Germany	Some concerns
44	**Davies 2017**	Cluster RCT	6967	40	Series	Adolescents	HPV	Pre-	Education	0.55	0.45	NA	Australia	Low
45	**DeCamp 2020**	RCT	157	NA	Any	Children	Influenza	Pre-	Healthcare	0.00	1.0	NA	United States	Low
46	**Dempsey 2019**	RCT	1294	NA	Series	Adolescents	HPV	Pre-	Healthcare	0.34	0.66	0.09	United States	Some concerns
47	**DiClemente 2015**	RCT	216	NA	Series	Adolescents	HPV	Pre-	Healthcare	0.50	0.50	NA	United States	Low
48	**Dini 2000**	Cluster RCT	1227	NA	Timely	Young children	Childhood Vaccines	Pre-	Healthcare	0.50	0.50	NA	United States	Some concerns
49	**Dombkowski 2014**	RCT	10175	NA	Series	Young children	Childhood Vaccines	Pre-	Healthcare	0.50	0.50	NA	United States	Some concerns
50	**Dombkowski 2017**	RCT	2348	NA	Any	Adolescents	Influenza	Pre-	Healthcare	0.49	0.51	NA	United States	High
50	**Dombkowski 2017**	Cluster RCT	2048	NA	Any	Adolescents	Influenza	Pre-	Healthcare	0.49	0.51	NA	United States	High
51	**Domek 2019**	RCT	720	NA	First	Young children	Childhood Vaccines	Pre-	Healthcare	0.00	1.0	NA	Guatemala*	High
52	**Doyle 2015**	RCT	233	NA	Series	Young children	Childhood Vaccines	Pre-	Community/Other	0.00	1.0	0.99	Ireland	Some concerns
53	**El-Mohandes, 2003**	RCT	286	NA	Series	Young children	Childhood Vaccines	Pre-	Community/Other	0.00	1.0	NA	United States	High
54	**Esposito 2018**	Cluster RCT	917	NA	Any	Adolescents	HPV	Pre-	Education	0.42	0.58	NA	Italy	Low
55	**Fernandez 2022**	RCT	1554	NA	Any	Adolescents	HPV	Pre-	Community/Other	0.06	0.94	0.08	United States	High
56	**Ferreira 2022**	Cluster RCT	238	6	Any	Adolescents	HPV	Post-	Education	0.00	1.0	NA	Brazil*	Some concerns
57	**Fiks 2013**	RCT	11434	NA	Series	Adolescents	HPV	Pre-	Healthcare	0.00	1.0	0.56	United States	Low
58	**Fitzpatrick 2018**	RCT	556	NA	Any	Adults	Hepatitis B	Pre-	Online	1.00	0.0	NA	China*	Some concerns
59	**Frew 2016**	RCT	106	NA	Any	Pregnant individuals	Influenza	Pre-	Healthcare	0.50	0.50	NA	United States	Some concerns
60	**Gerend 2021**	RCT	150	NA	First	Adults	HPV	Pre-	Healthcare	1.00	0.0	NA	United States	Some concerns
61	**Glanz 2017**	RCT	1093	NA	Timely	Young children	Childhood Vaccines	Pre-	Online	0.00	1.0	0.87	United States	Some concerns
62	**Glanz 2020**	RCT	824	NA	Timely	Young children	Childhood Vaccines	Pre-	Online	0.00	1.0	0.85	United States	Some concerns
63	**Goodman 2015**	RCT	105	NA	Any	Pregnant individuals	Influenza	Pre-	Healthcare	0.00	1.0	0.01	United States	Low
64	**Goodyear-Smith 2012**	Cluster RCT	5256	63	First	Young children	Childhood Vaccines	Pre-	Healthcare	0.50	0.5	NA	New Zealand	Low

	Study	Design	N	N clusters	Outcome	Age	Vaccine Type	Covid-19	Setting	% male	% female	% white	Country	RoB
**65**	**Grandahl 2016**	Cluster RCT	751	18	Any	Adolescents	HPV	Pre-	Education	0.48	0.52	NA	Sweden	High
**66**	**Gurfinkel 2021**	RCT	37003	NA	Series	Adolescents	HPV	Pre-	Healthcare	0.52	0.48	NA	United States	Some concerns
**67**	**Haff 2023**	RCT	3671	NA	Any	Adults	Covid-19	Post-	Healthcare	0.41	0.59	0.60	United States	Some concerns
**68**	**Hambidge 2009**	RCT	811	NA	Timely	Young children	Childhood Vaccines	Pre-	Healthcare	0.00	1.0	NA	United States	Low
**69**	**Hanley 2023**	RCT	7408	NA	Series	Adolescents	HPV	Post-	Healthcare	0.45	0.55	0.24	United States	Low
**70**	**Hannan 2013**	RCT	139	NA	Any	Young children	Childhood Vaccines	Pre-	Healthcare	0.00	1.0	NA	United States	Some concerns
**71**	**Harari 2008**	Cluster RCT	2503	2050	Any	Older adults	Influenza	Pre-	Healthcare	0.45	0.55	NA	United Kingdom	Some concerns
**72**	**Henrikson 2018**	RCT	1805	NA	Any	Adolescents	HPV	Pre-	Healthcare	0.52	0.48	0.58	United States	Some concerns
**73**	**Hess 2013**	Cluster RCT	11982	16	Any	Older adults	Shingles	Pre-	Healthcare	0.50	0.50	NA	United States	Some concerns
**74**	**Higginbotham 2012**	RCT	101	NA	Any	Adults	Influenza	Pre-	Healthcare	0.42	0.58	NA	United States	High
**75**	**Ho 2019**	Cluster RCT	8837	22	Any	Older adults	Influenza	Pre-	Healthcare	0.45	0.55	NA	Singapore	Low
**76**	**Hofstetter 2015**	RCT	2054	NA	Any	Young children	Childhood Vaccines	Pre-	Healthcare	0.51	0.49	NA	United States	Low
**77**	**Hofstetter 2015a**	RCT	5462	NA	Any	Children	Influenza	Pre-	Healthcare	0.51	0.49	NA	United States	Low
**78**	**Hopfer 2012**	RCT	404	NA	First	Adults	HPV	Pre-	Education	0.00	1.0	0.72	United States	Some concerns
**79**	**Howell-Jones 2023**	Cluster RCT	21786	257	Any	Children	Influenza	Pre-	Healthcare	0.51	0.49	NA	United Kingdom	Some concerns
**79**	**Howell-Jones 2023**	Cluster RCT	NA	2994	First	Children	Influenza	Pre-	Education	0.50	0.50	0.85	United Kingdom	Some concerns
**81**	**Hu 2017**	RCT	1252	NA	Series	Young children	Childhood Vaccines	Pre-	Healthcare	0.00	1.0	NA	China*	Some concerns
**82**	**Hu 2018**	RCT	204	NA	Any	Young children	Childhood Vaccines	Pre-	Healthcare	0.00	1.0	NA	China*	Low
**80**	**Hu 2021**	RCT	320	NA	Any	Older adults	Influenza	Post-	Healthcare	0.51	0.49	NA	Singapore	Low
**83**	**Huf 2024**	RCT	69805	NA	Any	Adults	Covid-19	Post-	Healthcare	0.49	0.51	0.45	United Kingdom	Low
**84**	**Hull 2002**	Cluster RCT	1318	1206	Any	Older adults	Influenza	Pre-	Healthcare	0.45	0.55	NA	United Kingdom	Some concerns
**85**	**Humiston 2014**	Cluster RCT	12490	31	Any	Children	Influenza	Pre-	Education	0.50	0.50	0.43	United States	Low
**85**	**Humiston 2014**	Cluster RCT	12876	32	Any	Children	Influenza	Pre-	Education	0.50	0.50	0.42	United States	Low
**86**	**Hurley 2018**	RCT	25039	NA	Any	Adults	Influenza	Pre-	Healthcare	0.29	0.71	NA	United States	Low

	Study	Design	N	N clusters	Outcome	Age	Vaccine Type	Covid-19	Setting	% male	% female	% white	Country	RoB
**87**	**Hurley 2018**	RCT	5332	NA	Any	Older adults	Influenza	Pre-	Healthcare	0.38	0.62	NA	United Kingdom	Low
**88**	**Hurley 2019**	RCT	616	NA	Any	Older adults	Childhood Vaccines, Influenza, Pneumo	Pre-	Healthcare	0.36	0.64	NA	United States	Low
**89**	**Hurtaud 2023**	Cluster RCT	1975	19	Any	Adults	Childhood Vaccines	Post-	Healthcare	0.50	0.50	NA	France	Some concerns
**90**	**Hwang 2010**	RCT	630	NA	Series	Adults	Hepatitis B	Pre-	Community/Other	0.75	0.25	0.09	United States	High
**90**	**Hwang 2010**	RCT	630	NA	Series	Adults	Hepatitis B	Pre-	Community/Other	0.79	0.21	0.12	United States	High
**91**	**Irigoyen 2006**	RCT	1662	NA	Any	Young children	Childhood Vaccines	Pre-	Healthcare	0.50	0.50	NA	United States	Low
**92**	**Isrctn, 2021**	Cluster RCT	41548	10032	Any	Adults	Covid-19	Post-	Healthcare	0.49	0.51	NA	Germany	Low
**93**	**Jackson 2011**	Cluster RCT	142	12	Any	Young children	Childhood Vaccines	Pre-	Healthcare	0.06	0.94	NA	United Kingdom	Some concerns
**94**	**Janitz 2023**	RCT	312	NA	Any	Adults	Covid-19	Post-	Community/Other	0.21	0.79	0.76	United States	High
**95**	**Jiang 2022**	RCT	350	NA	Any	Older adults	Influenza	Post-	Healthcare	0.45	0.55	NA	China*	Some concerns
**96**	**Johansen 2023**	Cluster RCT	964870	691820	Any	Older adults	Influenza	Post-	Healthcare	0.49	0.61	NA	Denmark	Low
**97**	**Johnson 2003**	RCT	32698	NA	Any	Older adults	Pneumo	Pre-	Community/Other	0.50	0.50	NA	United States	High
**98**	**Jordan 2015**	RCT	18186	NA	Any	Pregnant individuals	Influenza	Pre-	Healthcare	0.00	1.0	NA	United States	High
**99**	**Joseph 2016**	RCT	200	NA	First	Adolescents	HPV	Pre-	Healthcare	0.00	1.0	NA	United States	High
**100**	**Ju, 2024**	RCT	220	NA	Any	Adults	Covid-19	Post-	Online	0.34	0.66	NA	China*	High
**101**	**Juon 2016**	RCT	232	NA	Series	Adults	Hepatitis B	Pre-	Community/Other	0.43	0.57	NA	United States	Some concerns
**102**	**Juraskova 2011**	RCT	157	NA	Any	Adolescents	HPV	Pre-	Education	0.00	1.0	NA	Australia	Some concerns
**103**	**Kasting 2019**	RCT	1751	NA	Series	Adults	Hepatitis B	Pre-	Healthcare	0.60	0.40	NA	United States	Low
**106**	**Kempe 2001**	RCT	603	NA	Series	Young children	Childhood Vaccines	Post-	Healthcare	0.66	0.36	0.15	United States	High
**105**	**Kempe 2005**	RCT	5193	NA	Any	Children	Influenza	Pre-	Healthcare	0.50	0.50	NA	United States	Low
**104**	**Kempe 2012**	RCT	264	NA	Any	Adolescents	Childhood Vaccines, HPV, MenACWY	Pre-	Education	1.00	0.0	NA	United States	Some concerns
**107**	**Kempe 2016**	Cluster RCT	929	7	Series	Adolescents	HPV	Pre-	Healthcare	0.65	0.35	NA	United States	Some concerns
**108**	**Kempe 2020**	Cluster RCT	120130	188	Any	Children	Influenza	Pre-	Healthcare	0.50	0.50	NA	United States	Low

	Study	Design	N	N clusters	Outcome	Age	Vaccine Type	Covid-19	Setting	% male	% female	% white	Country	RoB
**109**	**Kerpelman 2000**	RCT	4150	2500	Series	Young children	Childhood Vaccines	Pre-	Community/Other	0.50	0.50	0.14	United States	Some concerns
**110**	**Khan 2023**	RCT	7411	NA	Any	Adolescents	HPV	Post-	Healthcare	0.52	0.48	0.25	United States	Low
**111**	**Kim 2020**	RCT	104	NA	Any	Adults	HPV	Pre-	Online	0.00	1.0	NA	United States	Some concerns
**112**	**Krieger 2000**	RCT	1246	NA	Any	Older adults	Influenza	Pre-	Community/Other	0.55	0.45	NA	United States	High
**113**	**Kulle, 2024**	Cluster RCT	20414	20	Any	Adults	Covid-19	Post-	Community/Other	0.50	0.50	NA	Switzerland	Some concerns
**114**	**Lau 2012**	RCT	742	NA	Any	Adults	Influenza	Pre-	Education	0.43	0.57	NA	Australia	Some concerns
**115**	**LeBaron 2004**	RCT	3050	NA	Series	Young children	Childhood Vaccines	Pre-	Community/Other	0.49	0.51	0.07	United States	Some concerns
**116**	**Lee 2020**	RCT	50286	NA	Any	Adults	Influenza	Pre-	Online	0.38	0.62	NA	United States	Low
**117**	**Lerner 2021**	RCT	22046	NA	Any	Children	Influenza	Post-	Healthcare	0.51	0.49	0.41	United States	Some concerns
**117**	**Lerner 2021**	RCT	22046	NA	Any	Children	Influenza	Post-	Healthcare	0.51	0.49	0.41	United States	Some concerns
**117**	**Lerner 2021**	RCT	689	NA	Series	Children	Influenza	Post-	Healthcare	0.51	0.49	0.41	United States	Some concerns
**118**	**Leung 2017**	RCT	529	NA	Any	Older adults	Influenza	Pre-	Healthcare	0.47	0.53	NA	Hong Kong SAR, China	Low
**119**	**Liao 2020**	RCT	365	NA	Any	Children	Influenza	Pre-	Online	0.00	1.0	NA	Hong Kong SAR, China	Some concerns
**120**	**Lieu 2022**	RCT	8287	NA	Any	Older adults	Covid-19	Post-	Healthcare	0.44	0.66	NA	United States	Low
**121**	**Lin 2020**	RCT	282	NA	Any	Young children	Childhood Vaccines	Pre-	Healthcare	0.23	0.77	NA	Taiwan, China	High
**122**	**Ma 2018**	Cluster RCT	1834	32	Series	Adults	Hepatitis B	Pre-	Community/Other	0.41	0.59	NA	United States	High
**123**	**Ma 2021**	RCT	180	NA	Series	Adolescents	HPV	Post-	Healthcare	0.36	0.64	NA	United States	Some concerns
**124**	**Mantzari 2015**	RCT	1000	NA	Series	Adolescents	HPV	Pre-	Healthcare	0.00	1.0	NA	United Kingdom	Low
**125**	**Mason 2000**	RCT	511	NA	Any	Young children	Childhood Vaccines	Pre-	Healthcare	0.50	0.50	NA	United Kingdom	Low
**126**	**Masson 2013**	RCT	489	NA	Series	Adults	Hepatitis B	Pre-	Healthcare	0.68	0.32	0.36	United States	Low
**127**	**McCaul 2002**	Cluster RCT	23733	49	First	Older adults	Influenza	Pre-	Healthcare	0.50	0.50	NA	United States	Some concerns
**128**	**Meharry 2014**	RCT	135	NA	Any	Pregnant individuals	Influenza	Pre-	Healthcare	0.50	0.50	0.31	United States	Some concerns
**129**	**Mehta 2022**	RCT	16045	NA	Series	Adults	Covid-19	Post-	Healthcare	0.41	0.59	0.52	United States	Some concerns
**130**	**Menzies 2020**	RCT	1594	NA	Timely	Young children	Childhood Vaccines	Pre-	Healthcare	0.50	0.50	NA	Australia	Low

	Study	Design	N	N clusters	Outcome	Age	Vaccine Type	Covid-19	Setting	% male	% female	% white	Country	RoB
**131**	**Moniz 2013**	RCT	216	NA	First	Pregnant individuals	Influenza	Pre-	Healthcare	0.50	0.50	0.26	United States	Low
**132**	**Munoz-Miralles 2022**	Cluster RCT	210	57	Series	Older adults	Influenza	Pre-	Healthcare	0.50	0.50	NA	Spain	Some concerns
**133**	**NCT05012163 2024**	RCT	57581	NA	Any	Adults	Influenza	Post-	Healthcare	0.42	0.58	0.94	United States	Low
**134**	**NCT05536674 (Daly, 2023)**	RCT	55176	NA	Any	Adolescents	HPV	Post-	Healthcare	0.00	1.0	NA	Georgia*	Low
**135**	**NCT05248399, 2022**	RCT	199	NA	Any	Adults	Covid-19	Post-	Community/Other	0.99	0.01	NA	United States	Low
**136**	**NCT05534061 2022**	RCT	349	NA	Any	Adults	Covid-19	Post-	Community/Other	0.63	0.37	0.64	United States	Low
**137**	**NCT05537441 2022**	RCT	124793	NA	Any	Adults	Influenza	Post-	Healthcare	0.53	0.47	NA	United States	Some concerns
**138**	**Nehme 2019**	RCT	25649	NA	Any	Adults	Influenza	Pre-	Healthcare	0.49	0.51	NA	United States	Low
**139**	**Nowalk 2010**	Cluster RCT	12222	54	Any	Adults	Influenza	Pre-	Community/Other	0.57	0.43	NA	United States	Some concerns
**140**	**Nyamathi 2009**	Cluster RCT	865	NA	Series	Adults	Hepatitis A, Hepatitis B	Pre-	Community/Other	0.77	0.23	0.15	United States	Some concerns
**141**	**Nyamathi 2010**	RCT	256	NA	Series	Adults	Hepatitis A, Hepatitis B	Pre-	Healthcare	0.55	0.45	0.13	United States	Some concerns
**142**	**O'Grady 2022**	RCT	196	NA	Series	Young children	Childhood Vaccines	Pre-	Healthcare	0.00	1.0	NA	Australia	Low
**143**	**O'Leary 2015**	RCT	4587	NA	Any	Adolescents	Childhood Vaccines, HPV, MenACWY	Pre-	Healthcare	0.54	0.46	NA	United States	Low
**144**	**O'Leary 2019**	RCT	1093	NA	Any	Pregnant individuals	Influenza	Pre-	Online	0.00	1.0	0.88	United States	Some concerns
**145**	**Omer 2022**	RCT	1045	NA	First	Pregnant individuals	Influenza	Pre-	Healthcare	0.00	1.00	0.55	United States	Low
**146**	**Osborne, 2023**	RCT	702	NA	Any	Adults	Influenza	Pre-	Online	0.28	0.72	0.84	United States	Some concerns
**147**	**Otsuka 2013**	RCT	674	NA	Any	Older adults	Shingles	Pre-	Healthcare	0.46	0.54	0.87	United States	Low
**147**	**Otsuka 2013**	RCT	1915	NA	Any	Older adults	Shingles	Pre-	Healthcare	0.44	0.56	0.72	United States	Low
**148**	**Otsuka-Ono 2019**	RCT	175	NA	Any	Young children	Hepatitis B	Pre-	Healthcare	0.50	0.50	NA	Japan	Some concerns
**149**	**Patel 2014**	Cluster RCT	365	10	Series	Adults	HPV	Pre-	Healthcare	0.00	1.00	0.57	United States	Some concerns
**150**	**Patel 2022**	RCT	74811	NA	Any	Adults	Influenza	Post-	Healthcare	0.44	0.56	0.71	United States	Low
**151**	**Porter-Jones 2009**	RCT	974	NA	First	Young children	Childhood Vaccines	Pre-	Online	0.53	0.47	NA	United Kingdom	Some concerns

	Study	Design	N	N clusters	Outcome	Age	Vaccine Type	Covid-19	Setting	% male	% female	% white	Country	RoB
**152**	**Pot 2017**	RCT	9124	NA	Any	Adolescents	HPV	Pre-	Online	0.50	0.50	NA	Netherlands	Some concerns
**153**	**Qin 2023**	RCT	100	NA	Series	Adolescents	HPV	Post-	Healthcare	0.16	0.84	NA	China*	Some concerns
**154**	**Quinlivan 2003**	RCT	139	NA	Series	Young children	Childhood Vaccines	Pre-	Community/Other	0.00	1.00	NA	Australia	Low
**155**	**Rand 2015**	RCT	3812	NA	Series	Adolescents	HPV	Pre-	Healthcare	0.55	0.45	NA	United States	Low
**156**	**Rand 2017**	RCT	749	NA	Series	Adolescents	HPV	Pre-	Healthcare	0.66	0.34	0.17	United States	Some concerns
**158**	**Reiter 2018**	RCT	150	NA	Series	Adolescents	HPV	Pre-	Online	1.00	0.00	0.55	United States	Some concerns
**157**	**Reiter 2023**	RCT	1227	NA	Series	Adolescents	HPV	Post-	Online	1.00	0.00	0.47	United States	Some concerns
**160**	**Richman 2014**	RCT	256	NA	Series	Adolescents	HPV	Pre-	Healthcare	0.12	0.88	0.09	United States	Some concerns
**159**	**Richman 2016**	RCT	264	NA	Series	Adolescents	HPV	Pre-	Healthcare	0.38	0.62	0.53	United States	Some concerns
**161**	**Roca 2012**	RCT	2402	NA	Any	Older adults	Influenza	Pre-	Healthcare	0.44	0.56	0.99	Spain	Low
**162**	**Rodriguez 2022**	Cluster RCT	541	7	Any	Adults	Covid-19	Post-	Healthcare	0.59	0.41	0.37	United States	High
**163**	**Rodriguez 2024**	Cluster RCT	767	6	Any	Adults	Influenza	Post-	Healthcare	0.53	0.47	0.37	United States	High
**164**	**Ronzani 2022**	RCT	2277	NA	Any	Adults	Covid-19	Post-	Online	0.50	0.50	NA	Italy	Some concerns
**165**	**Saaksvuori 2022**	Cluster RCT	47595	34690	Any	Older adults	Influenza	Pre-	Healthcare	0.44	0.56	NA	Finland	Low
**166**	**Saccardo 2024**	RCT	386615	NA	Any	Adults	Covid-19	Post-	Healthcare	0.42	0.58	NA	United States	Low
**167**	**Saitoh 2017**	Cluster RCT	188	9	Timely	Young children	Childhood Vaccines	Pre-	Healthcare	0.00	1.00	NA	Japan	Some concerns
**168**	**Santa Maria 2021**	RCT	519	NA	Any	Adolescents	HPV	Pre-	Education	0.10	0.90	0.03	United States	Low
**169**	**Scarinci 2020**	Cluster RCT	278	40	Series	Adolescents	HPV	Pre-	Community/Other	0.00	1.00	NA	United States	Some concerns
**170**	**Scott, 2019**	RCT	402	NA	Any	Children	Influenza	Pre-	Healthcare	0.05	0.95	NA	United States	Low
**171**	**Shegog 2022**	Cluster RCT	512	51	First	Adolescents	HPV	Pre-	Healthcare	0.05	0.95	0.56	United States	Some concerns
**172**	**Shen, 2024**	RCT	720	NA	Any	Adults	Influenza	Post-	Healthcare	0.46	0.54	NA	China*	Low
**173**	**Shourie 2013**	Cluster RCT	220	50	First	Young children	Childhood Vaccines	Post-	Online	0.50	0.50	0.91	United Kingdom	Low
**174**	**Si 2022**	Cluster RCT	3968	NA	Any	Adolescents	HPV	Post-	Education	0.00	1.00	NA	China*	Some concerns
**175**	**Sitler 2018**	RCT	129	NA	Any	Children	Influenza	Pre-	Healthcare	0.02	0.98	NA	United States	Some concerns

	Study	Design	N	N clusters	Outcome	Age	Vaccine Type	Covid-19	Setting	% male	% female	% white	Country	RoB
**177**	**Stockwell 2012**	RCT	361	NA	Any	Adolescents	Childhood Vaccines, MenACWY	Pre-	Healthcare	0.42	0.58	0.03	United States	Low
**178**	**Stockwell 2012**	RCT	174	NA	Any	Young children	Childhood Vaccines	Pre-	Healthcare	0.50	0.50	0.02	United States	Some concerns
**178**	**Stockwell 2012**	RCT	9213	NA	Any	Children	Influenza	Pre-	Healthcare	0.49	0.51	0.01	United States	Low
**180**	**Stockwell 2014**	RCT	1187	NA	Any	Pregnant individuals	Influenza	Pre-	Healthcare	0.00	1.00	NA	United States	Low
**176**	**Stockwell 2015**	RCT	662	NA	Any	Children	Influenza	Pre-	Healthcare	0.50	0.50	0.00	United States	Low
**179**	**Stockwell 2022**	RCT	2086	NA	Series	Children	Influenza	Pre-	Healthcare	0.11	0.89	0.43	United States	Low
**181**	**Stolpe 2019**	RCT	22301	NA	Any	Adults	Pneumo	Pre-	Healthcare	0.43	0.57	NA	United States	Low
**182**	**Strathdee 2023**	RCT	150	NA	Any	Adults	Covid-19	Post-	Community/Other	0.63	0.37	NA	United States	High
**183**	**Stuck 2015**	RCT	2284	NA	Any	Older adults	Influenza	Pre-	Healthcare	0.43	0.57	NA	Switzerland	Low
**184**	**Suh 2012**	RCT	1600	NA	Any	Adolescents	Childhood Vaccines, HPV	Pre-	Healthcare	0.39	0.61	NA	United States	Low
**185**	**Suzuki, 2022**	RCT	2175	NA	Any	Adolescents	HPV	Post-	Online	0.58	0.42	NA	Japan	Some concerns
**186**	**Sweeney 2014**	RCT	82	NA	Any	Adolescents	HPV	Pre-	Education	0.00	1.00	0.56	United States	Some concerns
**193**	**Szilagyi 2006**	RCT	3006	NA	Any	Adolescents	Childhood Vaccines	Pre-	Healthcare	0.51	0.49	NA	United States	Low
**192**	**Szilagyi 2011**	RCT	7546	NA	Series	Adolescents	HPV	Pre-	Healthcare	0.50	0.50	0.12	United States	Low
**189**	**Szilagyi 2013**	Cluster RCT	7404	5559	Series	Adolescents	HPV	Pre-	Healthcare	0.50	0.50	NA	United States	Low
**194**	**Szilagyi 2018**	Cluster RCT	18921	42	Any	Children	Influenza	Pre-	Education	0.50	0.50	NA	United States	Low
**195**	**Szilagyi 2019**	Cluster RCT	15768	NA	Any	Children	Influenza	Pre-	Education	0.50	0.50	NA	United States	Some concerns
**188**	**Szilagyi 2020**	RCT	164205	NA	Any	Adults	Influenza	Pre-	Healthcare	0.42	0.58	0.57	United States	Low
**190**	**Szilagyi 2020**	RCT	85776	NA	Any	Children	Influenza	Pre-	Healthcare	0.50	0.50	NA	United States	Low
**187**	**Szilagyi 2020**	RCT	62118	NA	Series	Adolescents	HPV	Pre-	Healthcare	0.53	0.47	NA	United States	Low
**191**	**Szilagyi 2024**	RCT	24681	NA	Any	Children	Influenza	Post-	Healthcare	0.50	0.50	NA	United States	Low
**191**	**Szilagyi, 2024**	RCT	79955	NA	Any	Adults	Influenza	Post-	Healthcare	0.50	0.50	NA	United States	Low
**191**	**Szilagyi, 2024**	RCT	31044	NA	Any	Older adults	Influenza	Post-	Healthcare	0.50	0.50	NA	United States	Low
**196**	**Tentori 2022**	RCT	1957	NA	Any	Adults	Covid-19	Post-	Healthcare	0.53	0.47	NA	Italy	Low
**197**	**Terrell-Perica 2001**	RCT	6528	NA	Any	Adults	Influenza, Pneumo	Pre-	Healthcare	0.44	054	0.25	United States	Some concerns

	Study	Design	N	N clusters	Outcome	Age	Vaccine Type	Covid-19	Setting	% male	% female	% white	Country	RoB
**198**	**Thilly, 2024**	Cluster RCT	14822	91	Any	Adolescents	HPV	Post-	Education	0.50	0.50	NA	France	Low
**199**	**Tiro 2015**	RCT	875	NA	Series	Adolescents	HPV	Pre-	Healthcare	0.50	0.50	NA	United States	Low
**200**	**Topp 2013**	RCT	201	NA	Series	Adults	Hepatitis B	Pre-	Healthcare	0.77	0.27	NA	Australia	Low
**201**	**Tubiana 2021**	Cluster RCT	1475	18	Any	Older adults	Influenza, Pneumo	Pre-	Healthcare	0.50	0.50	NA	France;Monaco	Some concerns
**202**	**Tull 2019**	RCT	4386	NA	Any	Adolescents	HPV	Pre-	Education	0.50	0.50	NA	Australia	Low
**203**	**Ueberroth 2022**	RCT	16728	NA	Any	Adults	Influenza	Post-	Healthcare	0.48	0.52	NA	United States	High
**204**	**Usami 2009**	Cluster RCT	1863	84	Any	Older adults	Influenza	Pre-	Healthcare	0.32	0.68	NA	Japan	Some concerns
**205**	**Vanderpool 2013**	RCT	344	NA	Series	Adolescents	HPV	Pre-	Community/Other	0.00	1.00	0.94	United States	Low
**206**	**Viver 2000**	RCT	264	NA	Series	Young children	Childhood Vaccines	Pre-	Healthcare	0.50	0.50	NA	United States	Some concerns
**207**	**Wang 2021**	RCT	624	NA	Series	Adults	HPV	Pre-	Community/Other	1.00	0.00	NA	Hong Kong SAR, China	Some concerns
**208**	**Wang 2023**	RCT	396	NA	Any	Older adults	Influenza	Post-	Online	0.37	0.63	NA	Hong Kong SAR, China	Some concerns
**209**	**Weaver 2014**	Cluster RCT	210	12	Series	Adults	Hepatitis B	Pre-	Healthcare	0.80	0.20	0.75	United Kingdom	Low
**210**	**Wijesundara 2020a**	RCT	39011	NA	Series	Adults	Influenza	Pre-	Healthcare	0.37	0..63	0.73	United States	Low
**210**	**Wijesundara 2020b**	RCT	58596	NA	Series	Adults	Influenza	Pre-	Healthcare	0.51	0.49	0.64	United States	Low
**211**	**Winston 2007**	RCT	2395	NA	Any	Older adults	Pneumo	Pre-	Healthcare	0.40	0.60	NA	United States	Low
**212**	**Wiseman 2016**	RCT	136	NA	Any	Children	Influenza	Pre-	Healthcare	0.06	0.94	0.02	United States	Low
**213**	**Wong 2016**	RCT	321	NA	Any	Pregnant individuals	Influenza	Pre-	Healthcare	0.00	1.00	NA	Hong Kong SAR, China	Some concerns
**214**	**Wouters, 2007**	RCT	615	NA	Series	Adults	Hepatitis B	Post-	Healthcare	0.07	0.93	NA	Belgium	High
**215**	**Wright 2012**	Cluster RCT	3979	11	Any	Adults	Influenza	Pre-	Healthcare	0.40	0.60	0.87	United States	Some concerns
**216**	**Wynn 2021**	RCT	956	NA	Series	Adolescents	HPV	Pre-	Healthcare	0.50	0.50	NA	United States	Low
**217**	**Xu 2022**	RCT	246	NA	Timely	Young children	Childhood Vaccines	Post-	Community/Other	0.15	0.85	NA	China*	Some concerns
**218**	**Yeung 2018**	RCT	833	NA	Any	Children	Influenza	Pre-	Healthcare	0.00	1.00	NA	Hong Kong SAR, China	Some concerns
**219**	**Yokum 2018**	RCT	227955	NA	Any	Older adults	Influenza	Pre-	Healthcare	0.45	0.55	NA	United States	Some concerns
**220**	**Yudin 2016**	RCT	317	NA	Any	Pregnant individuals	Influenza	Pre-	Healthcare	0.00	1.00	0.50	Canada	Some concerns

	Study	Design	N	N clusters	Outcome	Age	Vaccine Type	Covid-19	Setting	% male	% female	% white	Country	RoB
**221**	**Zhang 2018a**	RCT	312	NA	Any	Older adults	Influenza	Pre-	Community/Other	0.17	0.83	NA	Hong Kong SAR, China	Some concerns
**222**	**Zhang 2018b**	RCT	451	NA	Series	Adults	Hepatitis A, Hepatitis B	Pre-	Community/Other	0.94	0.06	0.35	United States	Low
**223**	**Zhang 2022**	RCT	946	NA	Any	Adolescents	HPV	Post-	Education	0.00	1.00	NA	China*	Some concerns
**224**	**Zuniga de Nuncio 2003**	RCT	348	NA	Timely	Young children	Childhood Vaccines	Pre-	Healthcare	0.00	1.00	NA	United States	Low

Footnotes

RCT: randomised controlled trial; Any: any vaccination dose; First: first vaccination dose; Timely: up-to-date vaccination; Series: completion of vaccination series; HPV: Human Papilloma Virus, Pneumo: Pneumococcal; RoB: Risk of Bias

*Upper-middle income countries

G: Intervention coding for all studies

	Study	Arm	PD	In	Hum	HC	Com	Int	EO	ASH	ASO	AD	Inc	CC	VS	DR	SF	DA	MI	Act
**1**	**Abroms 2023**	Control	NA	NA	NA	NA	NA	NA	NA	NA	NA	NA	NA	NA	NA	NA	NA	NA	NA	NA
**1**	**Abroms 2023**	Education	0	1	1	1	0	High	0	0	0	0	0	0	1	1	1	0	0	0
**2**	**Alonge 2023**	Control	NA	NA	NA	NA	NA	NA	NA	NA	NA	NA	NA	NA	NA	NA	NA	NA	NA	NA
**2**	**Alonge 2023**	Reminder	1	0	0	1	0	Low	0	0	0	0	0	0	0	0	0	0	0	1
**3**	**Anraad 2023**	Control	NA	NA	NA	NA	NA	NA	NA	NA	NA	NA	NA	NA	NA	NA	NA	NA	NA	NA
**3**	**Anraad 2023**	Education	0	1	1	1	0	Low	0	0	1	0	0	0	1	1	1	1	0	0
**4**	**Arnold 2022**	Control	NA	NA	NA	NA	NA	NA	NA	NA	NA	NA	NA	NA	NA	NA	NA	NA	NA	NA
**4**	**Arnold 2022**	Reminder	1	0	0	1	0	Medium	0	0	0	0	0	0	0	0	0	0	0	1
**5**	**Arthur 2002**	Access	1	1	1	1	0	Medium	1	0	0	0	0	0	1	0	0	0	0	1
**5**	**Arthur 2002**	Education and Reminder	1	0	0	1	0	Low	0	0	0	0	0	0	1	0	0	0	0	1
**6**	**Bartos 2022**	Control	NA	NA	NA	NA	NA	NA	NA	NA	NA	NA	NA	NA	NA	NA	NA	NA	NA	NA
**6**	**Bartos 2022**	Education	0	0	0	1	0	Low	0	0	0	0	0	0	1	0	1	0	0	0
**7**	**Bartu 2006**	Control	NA	NA	NA	NA	NA	NA	NA	NA	NA	NA	NA	NA	NA	NA	NA	NA	NA	NA
**7**	**Bartu 2006**	Education	1	1	1	1	0	Low	0	0	0	0	0	0	0	0	0	0	0	0
**8**	**Baskin 2018**	Reminder	1	0	0	0	1	Low	0	0	0	0	0	0	0	0	0	0	0	1
**8**	**Baskin 2018**	Affordability	1	0	0	0	1	Low	0	0	0	0	1	0	0	0	0	0	0	1
**9**	**Bastani 2022**	Multicomponent	1	1	1	1	0	Medium	0	0	0	0	0	1	1	1	0	0	0	1
**9**	**Bastani 2022**	Education	0	0	0	1	0	Low	0	0	0	0	0	0	1	1	0	0	0	0
**10**	**Bennett 2015**	Control	NA	NA	NA	NA	NA	NA	NA	NA	NA	NA	NA	NA	NA	NA	NA	NA	NA	NA
**10**	**Bennett 2015**	Education	0	1	0	1	0	Low	0	0	0	0	0	0	1	1	1	0	0	0
**12**	**Berg 2004**	Control	NA	NA	NA	NA	NA	NA	NA	NA	NA	NA	NA	NA	NA	NA	NA	NA	NA	NA
**12**	**Berg 2004**	Education	1	0	0	1	0	Medium	0	0	0	0	0	0	1	1	1	0	0	0
**11**	**Berg 2008**	Control	NA	NA	NA	NA	NA	NA	NA	NA	NA	NA	NA	NA	NA	NA	NA	NA	NA	NA
**11**	**Berg 2008**	Education	1	0	0	1	0	Low	0	0	0	0	0	0	1	1	0	0	0	0
**11**	**Berg 2008**	Education	1	0	0	1	0	Low	0	0	0	0	0	0	0	1	0	0	0	0
**13**	**Berkhout 2018**	Control	NA	NA	NA	NA	NA	NA	NA	NA	NA	NA	NA	NA	NA	NA	NA	NA	NA	NA
**13**	**Berkhout 2018**	Education	0	0	0	1	0	Low	0	0	0	0	0	0	1	1	1	0	0	0

	Study	Arm	PD	In	Hum	HC	Com	Int	EO	ASH	ASO	AD	Inc	CC	VS	DR	SF	DA	MI	Act
14	**Bernard-Genest 2021**	Control	NA	NA	NA	NA	NA	NA	NA	NA	NA	NA	NA	NA	NA	NA	NA	NA	NA	NA
14	**Bernard-Genest 2021**	Education and Reminder	1	1	1	1	0	Medium	0	0	0	0	0	0	1	1	0	0	0	1
15	**Berset 2022**	Control	NA	NA	NA	NA	NA	NA	NA	NA	NA	NA	NA	NA	NA	NA	NA	NA	NA	NA
15	**Berset 2022**	Reminder	1	0	0	1	0	Medium	0	0	0	0	0	0	0	0	0	0	0	1
15	**Berset 2022**	Reminder	1	0	0	1	0	Medium	0	0	0	0	0	0	0	0	0	0	0	1
16	**Berset 2023**	Reminder	1	0	0	1	0	Low	0	0	0	0	0	0	0	0	0	0	0	1
16	**Berset 2023**	Reminder	1	0	0	1	0	Medium	0	0	0	0	0	0	0	0	0	0	0	1
16	**Berset 2023**	Reminder	1	1	1	1	0	Medium	0	1	0	0	0	0	0	0	0	0	0	1
16	**Berset 2023**	Reminder	1	1	1	1	0	Medium	0	1	0	0	0	0	0	0	0	0	0	1
17	**Bethke 2024**	Education	0	1	1	1	0	Low	1	0	0	0	0	0	1	1	1	0	0	0
17	**Bethke 2024**	Access	1	1	1	1	0	Low	1	0	0	0	0	0	0	0	0	0	0	0
18	**Bian 2023**	Control	NA	NA	NA	NA	NA	NA	NA	NA	NA	NA	NA	NA	NA	NA	NA	NA	NA	NA
18	**Bian 2023**	Education and Reminder	1	1	0	0	1	High	0	0	0	0	0	0	1	1	0	0	0	1
19	**Borg 2018**	Control	NA	NA	NA	NA	NA	NA	NA	NA	NA	NA	NA	NA	NA	NA	NA	NA	NA	NA
19	**Borg 2018**	Education and Reminder	1	0	0	1	0	Low	0	0	0	0	0	0	1	1	0	0	0	1
19	**Borg 2018**	Education and Reminder	0	0	0	1	0	Low	0	0	0	0	0	0	1	1	0	0	0	1
20	**Bourgeois 2008**	Control	NA	NA	NA	NA	NA	NA	NA	NA	NA	NA	NA	NA	NA	NA	NA	NA	NA	NA
20	**Bourgeois 2008**	Education and Reminder	1	0	0	1	0	High	0	0	0	0	0	0	1	1	0	0	0	1
21	**Bowman, 2014**	Control	NA	NA	NA	NA	NA	NA	NA	NA	NA	NA	NA	NA	NA	NA	NA	NA	NA	NA
21	**Bowman, 2014**	Access	1	0	0	1	0	Medium	0	0	0	1	0	0	0	0	0	0	0	0
22	**Brigham 2012**	Control	NA	NA	NA	NA	NA	NA	NA	NA	NA	NA	NA	NA	NA	NA	NA	NA	NA	NA
22	**Brigham 2012**	Education and Reminder	1	1	1	1	0	Medium	0	1	0	0	0	0	1	1	0	0	0	1
22	**Brigham 2012**	Education and Reminder	1	1	1	1	0	Medium	0	0	0	0	0	0	1	1	0	0	0	1
23	**Bronchetti 2015**	Multicomponent	1	0	0	1	0	Medium	1	0	0	0	0	0	0	1	1	0	0	1
23	**Bronchetti 2015**	Affordability	1	0	0	1	0	Medium	1	0	0	0	1	0	1	0	1	0	0	1
23	**Bronchetti 2015**	Multicomponent	1	0	0	0	1	Medium	1	0	0	0	0	0	0	1	1	0	0	1

	Study	Arm	PD	In	Hum	HC	Com	Int	EO	ASH	ASO	AD	Inc	CC	VS	DR	SF	DA	MI	Act
**23**	**Bronchetti 2015**	Education and Reminder	1	0	0	1	0	Medium	0	0	0	0	0	0	1	0	1	0	0	1
**24**	**Burkhardt 2023**	Control	NA	NA	NA	NA	NA	NA	NA	NA	NA	NA	NA	NA	NA	NA	NA	NA	NA	NA
**24**	**Burkhardt 2023**	Reminder	1	0	0	1	0	Medium	0	0	0	0	0	0	0	0	0	0	0	1
**25**	**Buttenheim 2022**	Control	NA	NA	NA	NA	NA	NA	NA	NA	NA	NA	NA	NA	NA	NA	NA	NA	NA	NA
**25**	**Buttenheim 2022**	Reminder	1	0	0	1	0	Medium	0	0	0	0	0	0	0	0	0	0	0	1
**25**	**Buttenheim 2022**	Reminder	1	0	0	1	0	Medium	0	0	0	0	0	0	0	0	0	0	0	1
**26**	**Campos-Mercade 2021**	Control	NA	NA	NA	NA	NA	NA	NA	NA	NA	NA	NA	NA	NA	NA	NA	NA	NA	NA
**26**	**Campos-Mercade 2021**	Affordability	0	0	0	1	0	Medium	0	0	0	0	1	0	0	0	0	0	0	1
**26**	**Campos-Mercade 2021**	Reminder	0	0	0	1	0	Medium	0	0	0	0	0	0	0	0	1	0	0	1
**26**	**Campos-Mercade 2021**	Reminder	0	0	0	1	0	Medium	0	0	0	0	0	0	0	0	1	0	0	1
**26**	**Campos-Mercade 2021**	Education and Reminder	0	1	0	1	0	Medium	0	0	0	0	0	0	1	0	0	0	0	1
**26**	**Campos-Mercade 2021**	Reminder	0	0	0	1	0	Medium	0	0	0	0	0	0	0	0	0	0	0	1
**27**	**Cataldi 2024**	Control	NA	NA	NA	NA	NA	NA	NA	NA	NA	NA	NA	NA	NA	NA	NA	NA	NA	NA
**27**	**Cataldi 2024**	Education	0	0	0	0	1	Low	0	0	0	0	0	0	1	1	0	0	0	0
**28**	**CentersforDisease 2012**	Control	NA	NA	NA	NA	NA	NA	NA	NA	NA	NA	NA	NA	NA	NA	NA	NA	NA	NA
**28**	**CentersforDisease 2012**	Reminder	1	0	0	1	0	Low	0	0	0	0	0	0	0	0	0	0	0	1
**29**	**Chai 2013**	Control	NA	NA	NA	NA	NA	NA	NA	NA	NA	NA	NA	NA	NA	NA	NA	NA	NA	NA
**29**	**Chai 2013**	Education and Reminder	1	0	0	1	0	High	0	0	0	0	0	0	1	1	0	0	0	1
**30**	**Chang 2023**	Control	NA	NA	NA	NA	NA	NA	NA	NA	NA	NA	NA	NA	NA	NA	NA	NA	NA	NA
**30**	**Chang 2023**	Reminder	1	0	0	1	0	Low	0	0	0	0	0	0	0	0	0	0	0	1
**30**	**Chang 2023**	Multicomponent	1	0	0	1	0	Low	0	0	0	0	1	0	0	0	0	0	0	1
**31**	**Chao 2015**	Control	NA	NA	NA	NA	NA	NA	NA	NA	NA	NA	NA	NA	NA	NA	NA	NA	NA	NA
**31**	**Chao 2015**	Reminder	1	0	0	1	0	Low	0	0	0	0	0	0	0	0	0	0	0	1
**32**	**Chodick 2021**	Control	NA	NA	NA	NA	NA	NA	NA	NA	NA	NA	NA	NA	NA	NA	NA	NA	NA	NA
**32**	**Chodick 2021**	Education	0	0	0	1	0	High	0	0	0	0	0	0	1	1	0	0	0	0
**33**	**Clayton 2021**	Control	NA	NA	NA	NA	NA	NA	NA	NA	NA	NA	NA	NA	NA	NA	NA	NA	NA	NA
**33**	**Clayton 2021**	Education and Reminder	1	0	0	1	0	Low	0	0	0	0	0	0	1	0	0	0	0	1

	Study	Arm	PD	In	Hum	HC	Com	Int	EO	ASH	ASO	AD	Inc	CC	VS	DR	SF	DA	MI	Act
**33**	**Clayton 2021**	Education and Reminder	1	0	0	1	0	Low	0	0	0	0	0	0	1	1	1	0	0	1
**33**	**Clayton 2021**	Education and Reminder	1	0	0	1	0	Low	0	0	0	0	0	0	1	1	0	0	0	1
**33**	**Clayton 2021**	Education and Reminder	1	0	0	1	0	Low	0	0	0	0	0	0	1	0	0	0	0	1
**33**	**Clayton 2021**	Education and Reminder	1	0	0	1	0	Low	0	0	0	0	0	0	1	1	1	0	0	1
**33**	**Clayton 2021**	Education and Reminder	1	0	0	1	0	Low	0	0	0	0	0	0	1	1	0	0	0	1
**34**	**Coley 2018**	Control	NA	NA	NA	NA	NA	NA	NA	NA	NA	NA	NA	NA	NA	NA	NA	NA	NA	NA
**34**	**Coley 2018**	Education and Reminder	1	0	0	1	0	Low	0	0	0	0	0	0	1	1	0	0	0	1
**35**	**Conner 2017**	Control	NA	NA	NA	NA	NA	NA	NA	NA	NA	NA	NA	NA	NA	NA	NA	NA	NA	NA
**35**	**Conner 2017**	Reminder	0	0	0	1	0	Low	0	0	0	0	0	0	0	0	0	0	0	1
**36**	**Cox 2012**	Education and Reminder	1	1	1	1	0	High	0	1	0	0	0	0	1	1	0	0	0	1
**36**	**Cox 2012**	Education and Reminder	1	1	1	1	0	High	0	1	0	0	0	0	1	1	0	0	0	1
**37**	**Cutrona 2018**	Control	NA	NA	NA	NA	NA	NA	NA	NA	NA	NA	NA	NA	NA	NA	NA	NA	NA	NA
**37**	**Cutrona 2018**	Reminder	1	1	0	1	0	Low	1	0	0	0	0	0	1	1	0	0	0	1
**37**	**Cutrona 2018**	Education and Reminder	1	1	0	1	0	Medium	1	0	0	0	0	0	1	1	0	0	0	1
**37**	**Cutrona 2018**	Education	1	1	0	1	0	Low	1	0	0	0	0	0	1	1	0	0	0	0
**38**	**Dai 2021**	Control	NA	NA	NA	NA	NA	NA	NA	NA	NA	NA	NA	NA	NA	NA	NA	NA	NA	NA
**38**	**Dai 2021**	Reminder	1	0	0	1	0	Low	0	0	1	0	0	0	0	0	0	0	0	1
**38**	**Dai 2021**	Education and Reminder	1	0	0	1	0	Medium	0	0	0	0	0	0	1	1	0	0	0	1
**38**	**Dai 2021**	Control	NA	NA	NA	NA	NA	NA	NA	NA	NA	NA	NA	NA	NA	NA	NA	NA	NA	NA
**38**	**Dai 2021**	Reminder	1	0	0	1	0	Low	0	0	0	0	0	0	0	0	1	0	0	1
**38**	**Dai 2021**	Reminder	1	0	0	1	0	Low	0	0	0	0	0	0	0	0	1	0	0	1
**39**	**Dalby 2000**	Control	NA	NA	NA	NA	NA	NA	NA	NA	NA	NA	NA	NA	NA	NA	NA	NA	NA	NA
**39**	**Dalby 2000**	Access	1	1	1	1	0	High	1	0	0	0	0	0	0	0	0	0	0	1
**41**	**Daley 2002**	Control	NA	NA	NA	NA	NA	NA	NA	NA	NA	NA	NA	NA	NA	NA	NA	NA	NA	NA
**41**	**Daley 2002**	Education and Reminder	1	1	1	1	0	Medium	0	0	0	0	0	0	1	0	0	0	0	1
**40**	**Daley 2014**	Control	NA	NA	NA	NA	NA	NA	NA	NA	NA	NA	NA	NA	NA	NA	NA	NA	NA	NA

	Study	Arm	PD	In	Hum	HC	Com	Int	EO	ASH	ASO	AD	Inc	CC	VS	DR	SF	DA	MI	Act
**40**	**Daley 2014**	Access	0	1	1	1	0	Medium	1	0	0	0	0	1	0	0	0	0	0	0
**42**	**Daniels 2007**	Multicomponent	1	1	1	1	1	Low	1	0	0	0	0	0	0	0	0	0	0	0
**42**	**Daniels 2007**	Education and Reminder	1	0	0	1	1	Medium	0	0	0	0	0	0	1	0	0	0	0	1
**43**	**Dapp 2011**	Control	NA	NA	NA	NA	NA	NA	NA	NA	NA	NA	NA	NA	NA	NA	NA	NA	NA	NA
**43**	**Dapp 2011**	Education	1	1	1	1	0	High	0	0	0	0	0	0	0	0	1	0	0	0
**44**	**Davies 2017**	Control	NA	NA	NA	NA	NA	NA	NA	NA	NA	NA	NA	NA	NA	NA	NA	NA	NA	NA
**44**	**Davies 2017**	Education	1	1	1	1	1	Low	0	0	0	0	0	0	1	1	0	1	0	0
**45**	**DeCamp 2020**	Control	NA	NA	NA	NA	NA	NA	NA	NA	NA	NA	NA	NA	NA	NA	NA	NA	NA	NA
**45**	**DeCamp 2020**	Education and Reminder	1	1	0	1	0	High	0	0	0	0	0	0	0	0	1	0	0	1
**46**	**Dempsey 2019**	Control	NA	NA	NA	NA	NA	NA	NA	NA	NA	NA	NA	NA	NA	NA	NA	NA	NA	NA
**46**	**Dempsey 2019**	Education	0	1	0	1	0	Low	0	0	0	0	0	0	1	1	1	1	0	0
**46**	**Dempsey 2019**	Education	0	1	0	1	0	Low	0	0	0	0	0	0	1	1	0	0	0	0
**47**	**DiClemente 2015**	Control	NA	NA	NA	NA	NA	NA	NA	NA	NA	NA	NA	NA	NA	NA	NA	NA	NA	NA
**47**	**DiClemente 2015**	Education and Reminder	0	0	0	1	0	Medium	0	0	0	0	0	0	1	1	1	0	0	1
**48**	**Dini 2000**	Control	NA	NA	NA	NA	NA	NA	NA	NA	NA	NA	NA	NA	NA	NA	NA	NA	NA	NA
**48**	**Dini 2000**	Reminder	1	0	0	1	0	High	0	0	0	0	0	0	0	0	0	0	0	1
**48**	**Dini 2000**	Reminder	1	0	0	1	0	High	0	0	0	0	0	0	0	0	0	0	0	1
**48**	**Dini 2000**	Reminder	1	0	0	1	0	Medium	0	0	0	0	0	0	0	0	0	0	0	1
**49**	**Dombkowski 2014**	Control	NA	NA	NA	NA	NA	NA	NA	NA	NA	NA	NA	NA	NA	NA	NA	NA	NA	NA
**49**	**Dombkowski 2014**	Reminder	1	0	0	1	0	Low	0	0	0	0	0	0	0	0	0	0	0	1
**50**	**Dombkowski 2017**	Control	NA	NA	NA	NA	NA	NA	NA	NA	NA	NA	NA	NA	NA	NA	NA	NA	NA	NA
**50**	**Dombkowski 2017**	Reminder	1	0	0	1	0	Medium	0	0	0	0	0	0	0	0	0	0	0	1
**50**	**Dombkowski 2017**	Control	NA	NA	NA	NA	NA	NA	NA	NA	NA	NA	NA	NA	NA	NA	NA	NA	NA	NA
**50**	**Dombkowski 2017**	Reminder	1	0	0	1	0	Medium	0	0	0	0	0	0	0	0	0	0	0	1
**51**	**Domek 2019**	Control	NA	NA	NA	NA	NA	NA	NA	NA	NA	NA	NA	NA	NA	NA	NA	NA	NA	NA
**51**	**Domek 2019**	Reminder	1	0	0	1	0	High	0	0	0	0	0	0	0	0	0	0	0	1
**52**	**Doyle 2015**	Control	NA	NA	NA	NA	NA	NA	NA	NA	NA	NA	NA	NA	NA	NA	NA	NA	NA	NA
**52**	**Doyle 2015**	Education	1	1	1	0	1	Low	0	0	0	0	0	0	0	0	1	0	0	0

	Study	Arm	PD	In	Hum	HC	Com	Int	EO	ASH	ASO	AD	Inc	CC	VS	DR	SF	DA	MI	Act
53	**El-Mohandes, 2003**	Control	NA	NA	NA	NA	NA	NA	NA	NA	NA	NA	NA	NA	NA	NA	NA	NA	NA	NA
53	**El-Mohandes, 2003**	Education	1	1	1	1	0	High	0	0	0	0	0	0	0	0	1	0	0	0
54	**Esposito 2018**	Control	NA	NA	NA	NA	NA	NA	NA	NA	NA	NA	NA	NA	NA	NA	NA	NA	NA	NA
54	**Esposito 2018**	Education	0	0	0	1	0	Medium	0	0	0	0	0	0	0	1	0	0	0	0
54	**Esposito 2018**	Education	1	1	1	1	0	Medium	0	0	0	0	0	0	0	1	0	0	0	0
55	**Fernandez 2022**	Control	NA	NA	NA	NA	NA	NA	NA	NA	NA	NA	NA	NA	NA	NA	NA	NA	NA	NA
55	**Fernandez 2022**	Education	1	1	1	1	0	Low	0	1	0	0	0	0	0	0	0	0	0	0
56	**Ferreira 2022**	Control	NA	NA	NA	NA	NA	NA	NA	NA	NA	NA	NA	NA	NA	NA	NA	NA	NA	NA
56	**Ferreira 2022**	Education	1	0	0	0	1	High	0	0	0	0	0	0	0	0	0	0	0	0
57	**Fiks 2013**	Control	NA	NA	NA	NA	NA	NA	NA	NA	NA	NA	NA	NA	NA	NA	NA	NA	NA	NA
57	**Fiks 2013**	Education and Reminder	1	0	0	1	0	Medium	0	0	0	0	0	0	1	0	0	0	0	1
58	**Fitzpatrick 2018**	Control	NA	NA	NA	NA	NA	NA	NA	NA	NA	NA	NA	NA	NA	NA	NA	NA	NA	NA
58	**Fitzpatrick 2018**	Education	0	1	0	1	0	Medium	0	0	0	0	0	0	0	1	0	0	0	0
59	**Frew 2016**	Control	NA	NA	NA	NA	NA	NA	NA	NA	NA	NA	NA	NA	NA	NA	NA	NA	NA	NA
59	**Frew 2016**	Education	0	0	0	1	1	Low	0	0	0	0	0	0	0	0	1	0	0	0
59	**Frew 2016**	Education	0	1	0	1	0	Low	0	0	0	0	0	0	1	1	0	1	0	0
60	**Gerend 2021**	Control	NA	NA	NA	NA	NA	NA	NA	NA	NA	NA	NA	NA	NA	NA	NA	NA	NA	NA
60	**Gerend 2021**	Education and Reminder	1	0	0	1	0	High	0	0	0	0	0	0	1	1	1	0	0	1
61	**Glanz 2017**	Control	NA	NA	NA	NA	NA	NA	NA	NA	NA	NA	NA	NA	NA	NA	NA	NA	NA	NA
61	**Glanz 2017**	Education	0	1	1	1	0	High	0	0	0	0	0	0	1	1	0	0	0	0
61	**Glanz 2017**	Education	0	0	0	1	0	Low	0	0	0	0	0	0	1	1	0	0	0	0
62	**Glanz 2020**	Control	NA	NA	NA	NA	NA	NA	NA	NA	NA	NA	NA	NA	NA	NA	NA	NA	NA	NA
62	**Glanz 2020**	Education	0	1	0	1	0	High	0	0	0	0	0	0	1	1	0	0	0	0
62	**Glanz 2020**	Education	0	0	0	1	0	High	0	0	0	0	0	0	1	1	0	0	0	0
63	**Goodman 2015**	Control	NA	NA	NA	NA	NA	NA	NA	NA	NA	NA	NA	NA	NA	NA	NA	NA	NA	NA
63	**Goodman 2015**	Education	0	0	0	1	0	Low	0	0	0	0	0	0	1	1	0	0	0	0
64	**Goodyear-Smith 2012**	Control	NA	NA	NA	NA	NA	NA	NA	NA	NA	NA	NA	NA	NA	NA	NA	NA	NA	NA
64	**Goodyear-Smith 2012**	Education and Reminder	1	1	1	1	0	Medium	0	0	0	0	0	0	1	1	0	0	0	1

	Study	Arm	PD	In	Hum	HC	Com	Int	EO	ASH	ASO	AD	Inc	CC	VS	DR	SF	DA	MI	Act
65	**Grandahl 2016**	Control	NA	NA	NA	NA	NA	NA	NA	NA	NA	NA	NA	NA	NA	NA	NA	NA	NA	NA
65	**Grandahl 2016**	Education	1	1	1	1	0	Medium	0	0	0	0	0	0	1	1	0	0	0	0
66	**Gurfinkel 2021**	Control	NA	NA	NA	NA	NA	NA	NA	NA	NA	NA	NA	NA	NA	NA	NA	NA	NA	NA
66	**Gurfinkel 2021**	Reminder	1	0	0	1	0	Medium	0	0	0	0	0	0	0	0	0	0	0	1
66	**Gurfinkel 2021**	Reminder	1	0	0	1	0	Medium	0	0	0	0	0	0	0	0	0	0	0	1
67	**Haff 2023**	Control	NA	NA	NA	NA	NA	NA	NA	NA	NA	NA	NA	NA	NA	NA	NA	NA	NA	NA
67	**Haff 2023**	Access	1	0	0	1	0	Low	1	0	0	0	0	0	0	0	0	0	0	1
67	**Haff 2023**	Access	1	0	0	1	0	Low	1	0	0	0	0	0	0	0	1	0	0	0
68	**Hambidge 2009**	Control	NA	NA	NA	NA	NA	NA	NA	NA	NA	NA	NA	NA	NA	NA	NA	NA	NA	NA
68	**Hambidge 2009**	Education and Reminder	1	1	1	1	0	High	0	0	0	0	0	0	0	0	0	0	0	1
69	**Hanley 2023**	Control	NA	NA	NA	NA	NA	NA	NA	NA	NA	NA	NA	NA	NA	NA	NA	NA	NA	NA
69	**Hanley 2023**	Education	1	0	0	1	0	Medium	0	0	1	0	0	0	1	0	0	0	0	0
70	**Hannan 2013**	Control	NA	NA	NA	NA	NA	NA	NA	NA	NA	NA	NA	NA	NA	NA	NA	NA	NA	NA
70	**Hannan 2013**	Education	1	1	1	1	0	High	0	0	0	0	0	0	0	0	0	0	0	0
71	**Harari 2008**	Control	NA	NA	NA	NA	NA	NA	NA	NA	NA	NA	NA	NA	NA	NA	NA	NA	NA	NA
71	**Harari 2008**	Education	1	1	1	1	0	Medium	0	0	0	0	0	0	0	0	0	0	0	1
72	**Henrikson 2018**	Control	NA	NA	NA	NA	NA	NA	NA	NA	NA	NA	NA	NA	NA	NA	NA	NA	NA	NA
72	**Henrikson 2018**	Education and Reminder	1	1	0	1	0	Medium	0	0	0	0	0	0	1	0	0	0	0	1
73	**Hess 2013**	Control	NA	NA	NA	NA	NA	NA	NA	NA	NA	NA	NA	NA	NA	NA	NA	NA	NA	NA
73	**Hess 2013**	Reminder	1	0	0	1	0	Medium	0	0	0	0	0	0	0	1	0	0	0	1
74	**Higginbotham 2012**	Control	NA	NA	NA	NA	NA	NA	NA	NA	NA	NA	NA	NA	NA	NA	NA	NA	NA	NA
74	**Higginbotham 2012**	Access	1	1	1	1	0	Low	1	0	0	0	0	0	0	0	0	0	0	0
74	**Higginbotham 2012**	Reminder	1	1	1	1	0	Low	0	0	0	0	0	0	0	0	0	0	0	1
75	**Ho 2019**	Control	NA	NA	NA	NA	NA	NA	NA	NA	NA	NA	NA	NA	NA	NA	NA	NA	NA	NA
75	**Ho 2019**	Education	0	0	0	1	0	Medium	0	0	0	0	0	0	0	1	1	0	0	0
76	**Hofstetter 2015**	Control	NA	NA	NA	NA	NA	NA	NA	NA	NA	NA	NA	NA	NA	NA	NA	NA	NA	NA
76	**Hofstetter 2015**	Reminder	1	0	0	1	0	Medium	0	0	0	0	0	0	0	0	0	0	0	1
76	**Hofstetter 2015**	Reminder	1	0	0	1	0	Low	0	0	0	0	0	0	0	0	0	0	0	1

	Study	Arm	PD	In	Hum	HC	Com	Int	EO	ASH	ASO	AD	Inc	CC	VS	DR	SF	DA	MI	Act
**77**	**Hofstetter 2015a**	Control	NA	NA	NA	NA	NA	NA	NA	NA	NA	NA	NA	NA	NA	NA	NA	NA	NA	NA
**77**	**Hofstetter 2015a**	Education and Reminder	1	1	0	1	0	High	0	0	0	0	0	0	1	1	0	0	0	1
**77**	**Hofstetter 2015a**	Education and Reminder	1	0	0	1	0	High	0	0	0	0	0	0	0	1	0	0	0	1
**78**	**Kopfer 2012**	Control	NA	NA	NA	NA	NA	NA	NA	NA	NA	NA	NA	NA	NA	NA	NA	NA	NA	NA
**78**	**Kopfer 2012**	Education	0	0	0	0	1	Low	0	0	0	0	0	0	1	0	1	0	0	0
**78**	**Kopfer 2012**	Education	0	0	0	1	1	Low	0	0	0	0	0	0	1	1	1	0	0	0
**78**	**Kopfer 2012**	Education	0	0	0	1	0	Low	0	0	0	0	0	0	1	1	1	0	0	0
**79**	**Howell-Jones 2023**	Control	NA	NA	NA	NA	NA	NA	NA	NA	NA	NA	NA	NA	NA	NA	NA	NA	NA	NA
**79**	**Howell-Jones 2023**	Education	1	0	0	1	0	Low	0	0	0	0	0	0	1	1	1	0	0	1
**79**	**Howell-Jones 2023**	Control	NA	NA	NA	NA	NA	NA	NA	NA	NA	NA	NA	NA	NA	NA	NA	NA	NA	NA
**79**	**Howell-Jones 2023**	Education	1	0	0	1	0	Low	0	0	0	0	0	0	1	1	1	0	0	0
**79**	**Howell-Jones 2023**	Reminder	1	0	0	1	0	Low	0	0	0	0	0	0	0	0	0	0	0	1
**79**	**Howell-Jones 2023**	Education and Reminder	1	0	0	1	0	Low	0	0	0	0	0	0	1	1	1	0	0	1
**81**	**Hu 2017**	Control	NA	NA	NA	NA	NA	NA	NA	NA	NA	NA	NA	NA	NA	NA	NA	NA	NA	NA
**81**	**Hu 2017**	Education	1	1	1	1	0	Low	0	0	0	0	0	0	1	0	0	0	0	0
**87**	**Hu 2018**	Control	NA	NA	NA	NA	NA	NA	NA	NA	NA	NA	NA	NA	NA	NA	NA	NA	NA	NA
**87**	**Hu 2018**	Education	0	0	0	1	0	Low	0	0	0	0	0	0	1	1	0	0	0	0
**87**	**Hu 2018**	Education	0	0	0	1	0	Low	0	0	0	0	0	0	1	1	0	0	0	0
**80**	**Hu 2021**	Education	1	1	1	1	0	Medium	0	0	0	0	0	0	1	1	0	0	0	0
**80**	**Hu 2021**	Education	0	0	0	1	0	Low	0	0	0	0	0	0	1	1	0	0	0	0
**83**	**Huf 2024**	Control	NA	NA	NA	NA	NA	NA	NA	NA	NA	NA	NA	NA	NA	NA	NA	NA	NA	NA
**83**	**Huf 2024**	Reminder	1	0	0	1	0	Low	0	0	0	0	0	0	0	0	0	0	0	1
**83**	**Huf 2024**	Education and Reminder	1	0	0	1	0	Low	0	0	0	0	0	0	0	0	0	0	0	1
**84**	**Hull 2002**	Control	NA	NA	NA	NA	NA	NA	NA	NA	NA	NA	NA	NA	NA	NA	NA	NA	NA	NA
**84**	**Hull 2002**	Reminder	1	1	1	1	0	Medium	0	1	0	0	0	0	0	0	0	0	0	1
**85**	**Humiston 2014**	Control	NA	NA	NA	NA	NA	NA	NA	NA	NA	NA	NA	NA	NA	NA	NA	NA	NA	NA
**85**	**Humiston 2014**	Access	1	0	0	1	0	Medium	1	0	0	0	0	0	0	0	0	0	0	0
**85**	**Humiston 2014**	Control	NA	NA	NA	NA	NA	NA	NA	NA	NA	NA	NA	NA	NA	NA	NA	NA	NA	NA

	Study	Arm	PD	In	Hum	HC	Com	Int	EO	ASH	ASO	AD	Inc	CC	VS	DR	SF	DA	MI	Act
**85**	**Humiston 2014**	Access	1	0	0	1	0	Medium	1	0	0	0	0	0	0	0	0	0	0	0
**86**	**Hurley 2018**	Control	NA	NA	NA	NA	NA	NA	NA	NA	NA	NA	NA	NA	NA	NA	NA	NA	NA	NA
**86**	**Hurley 2018**	Reminder	1	0	0	1	0	Medium	0	0	0	0	0	0	0	0	0	0	0	1
**87**	**Hurley 2018**	Control	NA	NA	NA	NA	NA	NA	NA	NA	NA	NA	NA	NA	NA	NA	NA	NA	NA	NA
**87**	**Hurley 2018**	Reminder	1	0	0	1	0	Medium	0	0	0	0	0	0	0	0	0	0	0	1
**88**	**Hurley 2019**	Control	NA	NA	NA	NA	NA	NA	NA	NA	NA	NA	NA	NA	NA	NA	NA	NA	NA	NA
**88**	**Hurley 2019**	Reminder	1	0	0	1	0	Medium	0	0	0	0	0	0	0	0	0	0	0	1
**89**	**Hurtaud 2023**	Control	NA	NA	NA	NA	NA	NA	NA	NA	NA	NA	NA	NA	NA	NA	NA	NA	NA	NA
**89**	**Hurtaud 2023**	Education	1	0	0	1	0	Low	0	0	0	0	0	0	1	1	1	0	0	0
**90**	**Hwang 2010**	Control	NA	NA	NA	NA	NA	NA	NA	NA	NA	NA	NA	NA	NA	NA	NA	NA	NA	NA
**90**	**Hwang 2010**	Access	1	1	1	1	0	Medium	0	0	0	1	0	0	0	0	0	0	0	0
**90**	**Hwang 2010**	Education	1	1	1	0	1	Medium	0	0	0	0	0	0	1	1	1	0	0	0
**90**	**Hwang 2010**	Multicomponent	1	1	1	0	1	Medium	0	0	0	1	0	0	1	1	1	0	0	0
**91**	**Irigoyen 2006**	Control	NA	NA	NA	NA	NA	NA	NA	NA	NA	NA	NA	NA	NA	NA	NA	NA	NA	NA
**91**	**Irigoyen 2006**	Reminder	1	0	0	1	0	High	0	0	0	0	0	0	0	0	0	0	0	1
**91**	**Irigoyen 2006**	Reminder	1	0	0	1	0	Medium	0	0	0	0	0	0	0	0	0	0	0	1
**92**	**Isrctn, 2021**	Control	NA	NA	NA	NA	NA	NA	NA	NA	NA	NA	NA	NA	NA	NA	NA	NA	NA	NA
**92**	**Isrctn, 2021**	Affordability	1	0	0	1	0	Low	0	0	0	0	1	0	0	0	0	0	0	0
**93**	**Jackson 2011**	Control	NA	NA	NA	NA	NA	NA	NA	NA	NA	NA	NA	NA	NA	NA	NA	NA	NA	NA
**93**	**Jackson 2011**	Education	1	1	1	1	1	Low	0	0	0	0	0	0	1	1	1	0	0	0
**94**	**Janitz 2023**	Control	NA	NA	NA	NA	NA	NA	NA	NA	NA	NA	NA	NA	NA	NA	NA	NA	NA	NA
**94**	**Janitz 2023**	Education	0	0	0	1	0	Low	0	0	0	0	0	0	0	0	0	0	0	0
**94**	**Janitz 2023**	Education	0	1	0	1	0	Low	0	0	0	0	0	0	0	0	0	0	1	0
**94**	**Janitz 2023**	Education	0	1	0	1	0	Medium	0	0	0	0	0	0	0	0	0	0	1	0
**94**	**Janitz 2023**	Education and Reminder	0	1	0	1	0	Medium	0	0	0	0	0	0	0	0	0	0	1	1
**94**	**Janitz 2023**	Education and Reminder	1	1	0	1	0	Medium	0	0	0	0	0	0	0	0	0	0	1	1
**94**	**Janitz 2023**	Reminder	1	0	0	1	0	Low	0	0	0	0	0	0	0	0	0	0	0	1
**94**	**Janitz 2023**	Education and Reminder	1	0	0	1	0	Medium	0	0	0	0	0	0	0	0	0	0	0	1

	Study	Arm	PD	In	Hum	HC	Com	Int	EO	ASH	ASO	AD	Inc	CC	VS	DR	SF	DA	MI	Act
**95**	**Jiang 2022**	Control	NA	NA	NA	NA	NA	NA	NA	NA	NA	NA	NA	NA	NA	NA	NA	NA	NA	NA
**95**	**Jiang 2022**	Education	1	1	1	1	0	Low	0	0	0	0	0	0	1	1	0	0	0	0
**96**	**Johansen 2023**	Control	NA	NA	NA	NA	NA	NA	NA	NA	NA	NA	NA	NA	NA	NA	NA	NA	NA	NA
**96**	**Johansen 2023**	Education and Reminder	1	0	0	1	0	Medium	0	0	0	0	0	0	0	1	1	0	0	1
**97**	**Johnson 2003**	Education and Reminder	1	0	0	1	0	High	0	0	0	0	0	0	0	0	0	0	0	1
**97**	**Johnson 2003**	Reminder	1	0	0	1	0	Low	0	0	0	0	0	0	0	0	0	0	0	1
**98**	**Jordan 2015**	Education and Reminder	1	1	0	1	0	Medium	0	0	0	0	0	0	1	0	1	0	0	1
**98**	**Jordan 2015**	Reminder	1	0	0	1	0	Low	0	0	0	0	0	0	0	1	0	0	0	1
**99**	**Joseph 2016**	Control	NA	NA	NA	NA	NA	NA	NA	NA	NA	NA	NA	NA	NA	NA	NA	NA	NA	NA
**99**	**Joseph 2016**	Education	1	1	1	1	0	Medium	0	0	0	0	0	0	1	1	0	0	1	0
**100**	**Ju, 2024**	Control	NA	NA	NA	NA	NA	NA	NA	NA	NA	NA	NA	NA	NA	NA	NA	NA	NA	NA
**100**	**Ju, 2024**	Education	1	0	0	1	0	High	0	0	0	0	0	0	1	0	1	0	0	0
**101**	**Juon 2016**	Control	NA	NA	NA	NA	NA	NA	NA	NA	NA	NA	NA	NA	NA	NA	NA	NA	NA	NA
**101**	**Juon 2016**	Reminder	1	1	1	0	1	Medium	0	0	0	0	0	0	0	0	0	0	0	1
**102**	**Juraskova 2011**	Education	0	0	0	1	0	Low	0	0	0	0	0	0	1	1	0	0	0	0
**102**	**Juraskova 2011**	Education	0	0	0	1	0	Low	0	0	0	0	0	0	0	1	0	0	0	0
**103**	**Kasting 2019**	Education	1	1	1	1	0	Medium	1	0	0	0	0	0	1	1	0	0	0	0
**103**	**Kasting 2019**	Education	1	1	1	1	0	Medium	1	0	0	0	0	0	0	1	0	0	0	0
**103**	**Kasting 2019**	Education	1	1	1	1	0	Medium	1	0	0	0	0	0	0	1	0	0	0	0
**106**	**Kempe 2001**	Control	NA	NA	NA	NA	NA	NA	NA	NA	NA	NA	NA	NA	NA	NA	NA	NA	NA	NA
**106**	**Kempe 2001**	Reminder	1	1	1	1	0	High	0	0	0	0	0	0	0	0	0	0	0	1
**105**	**Kempe 2005**	Control	NA	NA	NA	NA	NA	NA	NA	NA	NA	NA	NA	NA	NA	NA	NA	NA	NA	NA
**105**	**Kempe 2005**	Reminder	1	0	0	1	0	Medium	0	0	0	0	0	0	0	0	0	0	0	1
**104**	**Kempe 2012**	Control	NA	NA	NA	NA	NA	NA	NA	NA	NA	NA	NA	NA	NA	NA	NA	NA	NA	NA
**104**	**Kempe 2012**	Reminder	1	0	0	1	0	Medium	0	0	0	0	0	0	0	0	0	0	0	1
**107**	**Kempe 2016**	Control	NA	NA	NA	NA	NA	NA	NA	NA	NA	NA	NA	NA	NA	NA	NA	NA	NA	NA
**107**	**Kempe 2016**	Reminder	1	1	0	1	0	High	0	0	0	0	0	0	0	0	0	0	0	1
**108**	**Kempe 2020**	Control	NA	NA	NA	NA	NA	NA	NA	NA	NA	NA	NA	NA	NA	NA	NA	NA	NA	NA

	Study	Arm	PD	In	Hum	HC	Com	Int	EO	ASH	ASO	AD	Inc	CC	VS	DR	SF	DA	MI	Act
**108**	**Kempe 2020**	Reminder	1	0	0	1	0	Low	0	0	0	0	0	0	0	0	0	0	0	1
**108**	**Kempe 2020**	Reminder	1	0	0	1	0	Medium	0	0	0	0	0	0	0	0	0	0	0	1
**108**	**Kempe 2020**	Reminder	1	0	0	1	0	Medium	0	0	0	0	0	0	0	0	0	0	0	1
**109**	**Kerpelman 2000**	Control	NA	NA	NA	NA	NA	NA	NA	NA	NA	NA	NA	NA	NA	NA	NA	NA	NA	NA
**109**	**Kerpelman 2000**	Affordability	1	0	0	1	0	High	0	0	0	0	0	0	0	0	0	0	0	1
**110**	**Khan 2023**	Control	NA	NA	NA	NA	NA	NA	NA	NA	NA	NA	NA	NA	NA	NA	NA	NA	NA	NA
**110**	**Khan 2023**	Reminder	1	0	0	1	0	Medium	0	0	0	0	0	0	0	0	0	0	0	1
**110**	**Khan 2023**	Reminder	1	0	0	1	0	Medium	0	0	0	0	0	0	0	0	0	0	0	1
**111**	**Kim 2020**	Education	0	0	0	1	0	Low	0	0	0	0	0	0	1	0	0	0	0	0
**111**	**Kim 2020**	Education	0	0	0	0	1	Low	0	0	0	0	0	0	0	0	1	0	0	0
**112**	**Krieger 2000**	Control	NA	NA	NA	NA	NA	NA	NA	NA	NA	NA	NA	NA	NA	NA	NA	NA	NA	NA
**112**	**Krieger 2000**	Education and Reminder	1	1	1	0	1	Medium	0	0	0	0	0	0	0	0	1	0	0	1
**113**	**Kulle, 2024**	Control	NA	NA	NA	NA	NA	NA	NA	NA	NA	NA	NA	NA	NA	NA	NA	NA	NA	NA
**113**	**Kulle, 2024**	Access	1	0	0	1	0	Low	1	0	0	0	0	0	0	0	0	0	0	1
**114**	**Lau 2012**	Control	NA	NA	NA	NA	NA	NA	NA	NA	NA	NA	NA	NA	NA	NA	NA	NA	NA	NA
**114**	**Lau 2012**	Education and Reminder	1	1	1	1	0	Medium	0	0	1	0	0	0	1	0	0	0	0	1
**115**	**LeBaron 2004**	Control	NA	NA	NA	NA	NA	NA	NA	NA	NA	NA	NA	NA	NA	NA	NA	NA	NA	NA
**115**	**LeBaron 2004**	Reminder	1	1	1	1	0	Medium	0	0	0	0	0	0	0	0	0	0	0	1
**115**	**LeBaron 2004**	Reminder	1	0	0	1	0	High	0	0	0	0	0	0	0	0	0	0	0	1
**115**	**LeBaron 2004**	Reminder	1	1	1	1	0	High	0	0	0	0	0	0	0	0	0	0	0	1
**116**	**Lee 2020**	Control	NA	NA	NA	NA	NA	NA	NA	NA	NA	NA	NA	NA	NA	NA	NA	NA	NA	NA
**116**	**Lee 2020**	Affordability	0	0	0	1	0	Medium	0	0	0	0	1	0	0	0	0	0	0	1
**116**	**Lee 2020**	Reminder	1	0	0	1	0	Medium	0	0	0	0	0	0	0	0	0	0	0	1
**117**	**Lerner 2021**	Control	NA	NA	NA	NA	NA	NA	NA	NA	NA	NA	NA	NA	NA	NA	NA	NA	NA	NA
**117**	**Lerner 2021**	Education and Reminder	1	0	0	1	0	Medium	0	0	0	0	0	0	1	1	0	0	0	1
**117**	**Lerner 2021**	Control	NA	NA	NA	NA	NA	NA	NA	NA	NA	NA	NA	NA	NA	NA	NA	NA	NA	NA
**117**	**Lerner 2021**	Education	1	0	0	1	0	Low	0	0	0	0	0	0	0	0	0	0	0	0
**117**	**Lerner 2021**	Control	NA	NA	NA	NA	NA	NA	NA	NA	NA	NA	NA	NA	NA	NA	NA	NA	NA	NA

	Study	Arm	PD	In	Hum	HC	Com	Int	EO	ASH	ASO	AD	Inc	CC	VS	DR	SF	DA	MI	Act
**117**	**Lerner 2021**	Reminder	1	0	0	1	0	Low	0	0	0	0	0	0	0	0	0	0	0	1
**118**	**Leung 2017**	Control	NA	NA	NA	NA	NA	NA	NA	NA	NA	NA	NA	NA	NA	NA	NA	NA	NA	NA
**118**	**Leung 2017**	Education	1	1	1	1	0	Medium	1	0	0	0	0	0	1	1	0	0	0	0
**119**	**Liao 2020**	Control	NA	NA	NA	NA	NA	NA	NA	NA	NA	NA	NA	NA	NA	NA	NA	NA	NA	NA
**119**	**Liao 2020**	Education and Reminder	0	1	1	1	1	High	0	0	0	0	0	0	1	1	1	0	0	1
**120**	**Lieu 2022**	Control	NA	NA	NA	NA	NA	NA	NA	NA	NA	NA	NA	NA	NA	NA	NA	NA	NA	NA
**120**	**Lieu 2022**	Education and Reminder	1	0	0	1	0	Medium	0	0	0	0	0	0	1	0	1	0	0	1
**120**	**Lieu 2022**	Education and Reminder	1	0	0	1	0	Medium	0	0	0	0	0	0	1	0	0	0	0	1
**121**	**Lin 2020**	Control	NA	NA	NA	NA	NA	NA	NA	NA	NA	NA	NA	NA	NA	NA	NA	NA	NA	NA
**121**	**Lin 2020**	Education	1	1	1	1	0	Low	0	0	0	0	0	0	1	1	0	1	0	0
**122**	**Ma 2018**	Control	NA	NA	NA	NA	NA	NA	NA	NA	NA	NA	NA	NA	NA	NA	NA	NA	NA	NA
**122**	**Ma 2018**	Education	1	1	1	1	1	Low	1	1	0	0	0	0	0	1	0	0	0	0
**123**	**Ma 2021**	Control	NA	NA	NA	NA	NA	NA	NA	NA	NA	NA	NA	NA	NA	NA	NA	NA	NA	NA
**123**	**Ma 2021**	Education and Reminder	1	1	1	1	1	High	0	0	0	0	0	0	1	1	1	0	0	1
**124**	**Mantzari 2015**	Control	NA	NA	NA	NA	NA	NA	NA	NA	NA	NA	NA	NA	NA	NA	NA	NA	NA	NA
**124**	**Mantzari 2015**	Affordability	1	0	0	1	0	Medium	0	0	0	0	1	0	0	0	0	0	0	1
**125**	**Mason 2000**	Control	NA	NA	NA	NA	NA	NA	NA	NA	NA	NA	NA	NA	NA	NA	NA	NA	NA	NA
**125**	**Mason 2000**	Reminder	1	0	0	1	0	Medium	0	0	0	0	0	0	0	0	0	0	0	1
**126**	**Masson 2013**	Education	1	1	1	1	0	Medium	0	0	0	0	0	0	0	0	0	0	0	0
**126**	**Masson 2013**	Multicomponent	1	1	1	1	0	High	1	1	0	0	0	1	0	0	0	0	1	0
**127**	**McCaul 2002**	Control	NA	NA	NA	NA	NA	NA	NA	NA	NA	NA	NA	NA	NA	NA	NA	NA	NA	NA
**127**	**McCaul 2002**	Reminder	1	0	0	1	0	Low	0	0	0	0	0	1	1	1	1	0	0	1
**127**	**McCaul 2002**	Education and Reminder	1	0	0	1	0	Low	0	0	0	0	0	1	1	1	0	0	0	1
**128**	**Meharry 2014**	Control	NA	NA	NA	NA	NA	NA	NA	NA	NA	NA	NA	NA	NA	NA	NA	NA	NA	NA
**128**	**Meharry 2014**	Education	0	0	0	1	0	Low	0	0	0	0	0	0	1	1	1	0	0	0
**128**	**Meharry 2014**	Education	1	1	1	1	0	Low	0	0	0	0	0	0	1	1	1	0	0	0
**129**	**Mehta 2022**	Reminder	1	1	1	1	0	Medium	0	1	0	0	0	0	0	0	0	0	0	1

	Study	Arm	PD	In	Hum	HC	Com	Int	EO	ASH	ASO	AD	Inc	CC	VS	DR	SF	DA	MI	Act
**129**	**Mehta 2022**	Reminder	1	0	0	1	0	High	0	1	0	0	0	0	0	0	0	0	0	1
**130**	**Menzies 2020**	Control	NA	NA	NA	NA	NA	NA	NA	NA	NA	NA	NA	NA	NA	NA	NA	NA	NA	NA
**130**	**Menzies 2020**	Reminder	1	0	0	1	0	High	0	0	0	0	0	0	0	0	0	0	0	1
**130**	**Menzies 2020**	Reminder	1	0	0	1	0	Low	0	0	0	0	0	0	0	0	0	0	0	1
**130**	**Menzies 2020**	Reminder	1	0	0	1	0	High	0	0	0	0	0	0	0	0	0	0	0	1
**131**	**Moniz 2013**	Control	NA	NA	NA	NA	NA	NA	NA	NA	NA	NA	NA	NA	NA	NA	NA	NA	NA	NA
**131**	**Moniz 2013**	Education	1	0	0	1	0	High	0	0	0	0	0	0	1	0	0	0	0	0
**132**	**Munoz-Miralles 2022**	Control	NA	NA	NA	NA	NA	NA	NA	NA	NA	NA	NA	NA	NA	NA	NA	NA	NA	NA
**132**	**Munoz-Miralles 2022**	Education	1	1	1	1	0	Low	0	0	0	0	0	0	1	1	0	0	0	0
**133**	**NCT05012163 2024**	Control	NA	NA	NA	NA	NA	NA	NA	NA	NA	NA	NA	NA	NA	NA	NA	NA	NA	NA
**133**	**NCT05012163 2024**	Multicomponent	1	0	0	1	0	Medium	0	0	0	0	1	0	0	0	0	0	0	1
**133**	**NCT05012163 2024**	Affordability	1	0	0	1	0	Medium	0	0	0	0	1	0	0	0	0	0	0	1
**133**	**NCT05012163 2024**	Reminder	1	0	0	1	0	Medium	0	0	0	0	0	0	0	0	0	0	0	1
**134**	**NCT05536674 (Daly, 2023)**	Control	NA	NA	NA	NA	NA	NA	NA	NA	NA	NA	NA	NA	NA	NA	NA	NA	NA	NA
**134**	**NCT05536674 (Daly, 2023)**	Reminder	1	0	0	1	0	High	0	0	0	0	0	0	0	0	0	0	0	1
**134**	**NCT05536674 (Daly, 2023)**	Reminder	1	0	0	1	0	High	0	0	0	0	0	0	1	0	0	0	0	1
**135**	**NCT05248399, 2022**	Control	NA	NA	NA	NA	NA	NA	NA	NA	NA	NA	NA	NA	NA	NA	NA	NA	NA	NA
**135**	**NCT05248399, 2022**	Education	1	1	1	0	1	Low	0	1	0	0	0	0	0	0	1	0	0	0
**136**	**NCT05534061 2022**	Multicomponent	1	1	1	1	0	Low	1	0	0	0	1	0	0	0	0	0	1	0
**136**	**NCT05534061 2022**	Affordability	1	1	1	1	0	Low	1	0	0	0	1	0	0	0	0	0	0	0
**137**	**NCT05537441 2022**	Control	NA	NA	NA	NA	NA	NA	NA	NA	NA	NA	NA	NA	NA	NA	NA	NA	NA	NA
**137**	**NCT05537441 2022**	Reminder	1	0	0	1	0	Medium	0	0	0	0	0	1	0	0	0	0	0	1
**138**	**Nehme 2019**	Control	NA	NA	NA	NA	NA	NA	NA	NA	NA	NA	NA	NA	NA	NA	NA	NA	NA	NA
**138**	**Nehme 2019**	Multicomponent	1	0	0	1	0	Medium	0	0	0	0	1	0	0	0	0	0	0	1
**138**	**Nehme 2019**	Multicomponent	1	0	0	1	0	High	0	0	0	0	1	0	0	0	0	0	0	1
**139**	**Nowalk 2010**	Control	NA	NA	NA	NA	NA	NA	NA	NA	NA	NA	NA	NA	NA	NA	NA	NA	NA	NA
**139**	**Nowalk 2010**	Reminder	1	0	0	1	0	Medium	0	0	0	0	0	0	0	0	0	0	0	1
**139**	**Nowalk 2010**	Affordability	1	0	0	1	0	High	0	0	0	0	1	0	0	0	0	0	0	1
**140**	**Nyamathi 2009**	Multicomponent	1	1	1	1	0	High	0	0	0	0	1	0	1	1	0	0	0	0

	Study	Arm	PD	In	Hum	HC	Com	Int	EO	ASH	ASO	AD	Inc	CC	VS	DR	SF	DA	MI	Act
**140**	**Nyamathi 2009**	Multicomponent	1	1	1	1	0	Medium	0	0	0	0	1	0	1	1	0	0	0	0
**140**	**Nyamathi 2009**	Multicomponent	1	1	1	1	0	Medium	0	0	0	0	1	0	1	1	0	0	0	0
**141**	**Nyamathi 2010**	Education and Reminder	1	1	1	1	0	Medium	0	0	0	0	0	0	0	0	0	0	1	1
**141**	**Nyamathi 2010**	Education and Reminder	1	1	1	1	0	Medium	0	0	0	0	0	0	0	0	0	0	1	1
**141**	**Nyamathi 2010**	Education and Reminder	1	1	1	1	0	Medium	0	0	0	0	0	0	0	0	0	0	0	1
**142**	**O'Grady 2022**	Control	NA	NA	NA	NA	NA	NA	NA	NA	NA	NA	NA	NA	NA	NA	NA	NA	NA	NA
**142**	**O'Grady 2022**	Reminder	1	0	0	1	0	High	0	0	0	0	0	0	0	0	0	0	0	1
**142**	**O'Grady 2022**	Multicomponent	1	1	1	1	0	High	1	0	0	0	0	0	0	0	0	0	0	1
**143**	**O'Leary 2015**	Control	NA	NA	NA	NA	NA	NA	NA	NA	NA	NA	NA	NA	NA	NA	NA	NA	NA	NA
**143**	**O'Leary 2015**	Reminder	1	1	1	1	0	High	0	0	0	0	0	0	0	0	0	0	0	1
**144**	**O'Leary 2019**	Control	NA	NA	NA	NA	NA	NA	NA	NA	NA	NA	NA	NA	NA	NA	NA	NA	NA	NA
**144**	**O'Leary 2019**	Education	0	1	1	1	0	High	0	0	0	0	0	0	1	1	0	0	0	0
**144**	**O'Leary 2019**	Education	0	0	0	1	0	Low	0	0	0	0	0	0	1	1	0	0	0	0
**145**	**Omer 2022**	Control	NA	NA	NA	NA	NA	NA	NA	NA	NA	NA	NA	NA	NA	NA	NA	NA	NA	NA
**145**	**Omer 2022**	Education	0	0	0	1	0	High	0	0	0	0	0	0	1	1	0	0	0	0
**146**	**Osborne, 2023**	Control	NA	NA	NA	NA	NA	NA	NA	NA	NA	NA	NA	NA	NA	NA	NA	NA	NA	NA
**146**	**Osborne, 2023**	Education	0	0	0	1	0	High	0	0	0	0	0	0	0	0	0	0	0	0
**147**	**Otsuka 2013**	Control	NA	NA	NA	NA	NA	NA	NA	NA	NA	NA	NA	NA	NA	NA	NA	NA	NA	NA
**147**	**Otsuka 2013**	Education	0	0	0	1	0	Medium	0	0	0	0	0	0	0	0	0	0	0	0
**147**	**Otsuka 2013**	Control	NA	NA	NA	NA	NA	NA	NA	NA	NA	NA	NA	NA	NA	NA	NA	NA	NA	NA
**147**	**Otsuka 2013**	Education	1	0	0	1	0	Medium	0	0	0	0	0	0	0	0	0	0	0	0
**148**	**Otsuka-Ono 2019**	Control	NA	NA	NA	NA	NA	NA	NA	NA	NA	NA	NA	NA	NA	NA	NA	NA	NA	NA
**148**	**Otsuka-Ono 2019**	Education	1	1	1	1	0	Medium	0	0	0	0	0	0	1	0	1	0	0	0
**149**	**Patel 2014**	Control	NA	NA	NA	NA	NA	NA	NA	NA	NA	NA	NA	NA	NA	NA	NA	NA	NA	NA
**149**	**Patel 2014**	Reminder	1	0	0	1	0	Medium	0	0	0	0	0	0	0	0	0	0	0	1
**150**	**Patel 2022**	Control	NA	NA	NA	NA	NA	NA	NA	NA	NA	NA	NA	NA	NA	NA	NA	NA	NA	NA
**150**	**Patel 2022**	Reminder	1	0	0	1	0	Medium	0	0	0	0	0	0	0	0	0	0	0	1
**151**	**Porter-Jones 2009**	Control	NA	NA	NA	NA	NA	NA	NA	NA	NA	NA	NA	NA	NA	NA	NA	NA	NA	NA

	Study	Arm	PD	In	Hum	HC	Com	Int	EO	ASH	ASO	AD	Inc	CC	VS	DR	SF	DA	MI	Act
**151**	**Porter-Jones 2009**	Education	0	0	0	1	0	Low	0	0	0	0	0	0	1	1	0	0	0	0
**152**	**Pot 2017**	Control	NA	NA	NA	NA	NA	NA	NA	NA	NA	NA	NA	NA	NA	NA	NA	NA	NA	NA
**152**	**Pot 2017**	Education	0	1	0	1	0	Low	0	0	0	0	0	0	1	1	0	1	0	0
**153**	**Qin 2023**	Control	NA	NA	NA	NA	NA	NA	NA	NA	NA	NA	NA	NA	NA	NA	NA	NA	NA	NA
**153**	**Qin 2023**	Affordability	1	0	0	1	0	Low	0	0	0	0	0	1	0	0	0	0	0	0
**154**	**Quinlivan 2003**	Control	NA	NA	NA	NA	NA	NA	NA	NA	NA	NA	NA	NA	NA	NA	NA	NA	NA	NA
**154**	**Quinlivan 2003**	Education	1	1	1	1	0	Medium	0	1	0	0	0	0	0	0	0	0	0	0
**155**	**Rand 2015**	Control	NA	NA	NA	NA	NA	NA	NA	NA	NA	NA	NA	NA	NA	NA	NA	NA	NA	NA
**155**	**Rand 2015**	Reminder	1	0	0	1	0	Medium	0	0	0	0	0	0	0	0	0	0	0	1
**156**	**Rand 2017**	Control	NA	NA	NA	NA	NA	NA	NA	NA	NA	NA	NA	NA	NA	NA	NA	NA	NA	NA
**156**	**Rand 2017**	Reminder	1	0	0	1	0	Medium	0	0	0	0	0	0	0	0	0	0	0	1
**156**	**Rand 2017**	Reminder	1	0	0	1	0	Medium	0	0	0	0	0	0	0	0	0	0	0	1
**158**	**Reiter 2018**	Control	NA	NA	NA	NA	NA	NA	NA	NA	NA	NA	NA	NA	NA	NA	NA	NA	NA	NA
**158**	**Reiter 2018**	Education and Reminder	1	1	0	1	0	High	0	0	0	0	0	0	1	1	1	0	0	1
**157**	**Reiter 2023**	Control	NA	NA	NA	NA	NA	NA	NA	NA	NA	NA	NA	NA	NA	NA	NA	NA	NA	NA
**157**	**Reiter 2023**	Education and Reminder	1	1	0	1	0	High	0	0	0	0	0	0	1	1	0	0	0	1
**157**	**Reiter 2023**	Education and Reminder	1	0	0	1	0	High	0	0	0	0	0	0	1	1	0	0	0	1
**160**	**Richman 2014**	Control	NA	NA	NA	NA	NA	NA	NA	NA	NA	NA	NA	NA	NA	NA	NA	NA	NA	NA
**160**	**Richman 2014**	Reminder	1	0	0	1	0	High	0	0	0	0	0	0	0	0	0	0	0	1
**159**	**Richman 2016**	Control	NA	NA	NA	NA	NA	NA	NA	NA	NA	NA	NA	NA	NA	NA	NA	NA	NA	NA
**159**	**Richman 2016**	Reminder	1	0	0	1	0	High	0	0	0	0	0	0	0	0	0	0	0	1
**161**	**Roca 2012**	Control	NA	NA	NA	NA	NA	NA	NA	NA	NA	NA	NA	NA	NA	NA	NA	NA	NA	NA
**161**	**Roca 2012**	Education	1	0	0	1	0	Low	0	0	0	0	0	0	1	1	0	0	0	0
**162**	**Rodriguez 2022**	Control	NA	NA	NA	NA	NA	NA	NA	NA	NA	NA	NA	NA	NA	NA	NA	NA	NA	NA
**162**	**Rodriguez 2022**	Education	1	0	0	1	0	Low	0	0	0	0	0	0	0	0	1	0	0	0
**163**	**Rodriguez 2024**	Control	NA	NA	NA	NA	NA	NA	NA	NA	NA	NA	NA	NA	NA	NA	NA	NA	NA	NA
**163**	**Rodriguez 2024**	Education and Reminder	1	1	1	1	0	Medium	0	0	0	0	0	0	1	0	0	0	0	0
**163**	**Rodriguez 2024**	Reminder	1	1	1	1	0	Low	0	0	0	0	0	0	0	0	0	0	0	1

	Study	Arm	PD	In	Hum	HC	Com	Int	EO	ASH	ASO	AD	Inc	CC	VS	DR	SF	DA	MI	Act
**164**	**Ronzani 2022**	Education and Reminder	0	1	0	1	0	High	0	0	0	0	0	0	1	0	0	0	0	1
**164**	**Ronzani 2022**	Education and Reminder	0	1	0	1	0	High	0	0	0	0	0	0	1	0	1	0	0	1
**165**	**Saaksvuori 2022**	Control	NA	NA	NA	NA	NA	NA	NA	NA	NA	NA	NA	NA	NA	NA	NA	NA	NA	NA
**165**	**Saaksvuori 2022**	Multicomponent	1	0	0	1	0	Low	1	0	0	0	0	0	1	1	0	0	0	1
**165**	**Saaksvuori 2022**	Multicomponent	1	0	0	1	0	Low	1	0	0	0	0	0	1	1	1	0	0	1
**166**	**Saccardo 2024**	Control	NA	NA	NA	NA	NA	NA	NA	NA	NA	NA	NA	NA	NA	NA	NA	NA	NA	NA
**166**	**Saccardo 2024**	Reminder	1	0	0	1	0	Low	0	0	0	0	0	0	0	0	0	0	0	1
**167**	**Saitoh 2017**	Education	1	1	1	1	0	Medium	0	0	0	0	0	0	1	1	0	0	0	0
**167**	**Saitoh 2017**	Education	0	0	0	1	0	Low	0	0	0	0	0	0	1	1	0	0	0	0
**168**	**Santa Maria 2021**	Control	NA	NA	NA	NA	NA	NA	NA	NA	NA	NA	NA	NA	NA	NA	NA	NA	NA	NA
**168**	**Santa Maria 2021**	Education	1	1	1	1	0	High	1	1	0	0	0	0	0	0	0	0	0	1
**169**	**Scarinci 2020**	Control	NA	NA	NA	NA	NA	NA	NA	NA	NA	NA	NA	NA	NA	NA	NA	NA	NA	NA
**169**	**Scarinci 2020**	Education	1	1	1	0	1	High	0	0	0	0	0	0	1	1	1	0	0	0
**170**	**Scott, 2019**	Control	NA	NA	NA	NA	NA	NA	NA	NA	NA	NA	NA	NA	NA	NA	NA	NA	NA	NA
**170**	**Scott, 2019**	Education	0	0	0	1	0	Low	0	0	0	0	0	0	1	1	1	0	0	0
**170**	**Scott, 2019**	Education	0	0	0	1	0	Low	0	0	0	0	0	0	1	1	1	0	0	0
**171**	**Shegog 2022**	Control	NA	NA	NA	NA	NA	NA	NA	NA	NA	NA	NA	NA	NA	NA	NA	NA	NA	NA
**171**	**Shegog 2022**	Education and Reminder	0	0	0	1	0	Medium	0	0	1	0	0	0	1	1	1	0	0	1
**172**	**Shen, 2024**	Control	NA	NA	NA	NA	NA	NA	NA	NA	NA	NA	NA	NA	NA	NA	NA	NA	NA	NA
**172**	**Shen, 2024**	Affordability	1	0	0	1	0	Low	0	0	0	0	1	0	0	0	0	0	0	0
**173**	**Shourie 2013**	Control	NA	NA	NA	NA	NA	NA	NA	NA	NA	NA	NA	NA	NA	NA	NA	NA	NA	NA
**173**	**Shourie 2013**	Education	0	1	0	1	0	Low	0	0	0	0	0	0	1	1	0	1	0	0
**173**	**Shourie 2013**	Education	0	0	0	1	0	Low	0	0	0	0	0	0	1	1	0	0	0	0
**174**	**Si 2022**	Control	NA	NA	NA	NA	NA	NA	NA	NA	NA	NA	NA	NA	NA	NA	NA	NA	NA	NA
**174**	**Si 2022**	Education	0	0	0	1	0	High	0	0	0	0	0	0	0	1	1	0	0	0
**175**	**Sitler 2018**	Control	NA	NA	NA	NA	NA	NA	NA	NA	NA	NA	NA	NA	NA	NA	NA	NA	NA	NA
**175**	**Sitler 2018**	Access	1	1	1	1	0	Low	1	0	0	0	0	0	0	0	0	0	0	0
**177**	**Stockwell 2012**	Control	NA	NA	NA	NA	NA	NA	NA	NA	NA	NA	NA	NA	NA	NA	NA	NA	NA	NA

	Study	Arm	PD	In	Hum	HC	Com	Int	EO	ASH	ASO	AD	Inc	CC	VS	DR	SF	DA	MI	Act
**177**	**Stockwell 2012**	Reminder	1	0	0	1	0	High	0	0	0	0	0	0	0	0	0	0	0	1
**177**	**Stockwell 2012**	Reminder	1	0	0	1	0	Low	1	0	0	0	0	0	0	0	0	0	0	1
**177**	**Stockwell 2012**	Reminder	1	0	0	1	0	Medium	1	0	0	0	0	0	0	0	0	0	0	1
**178**	**Stockwell 2012**	Control	NA	NA	NA	NA	NA	NA	NA	NA	NA	NA	NA	NA	NA	NA	NA	NA	NA	NA
**178**	**Stockwell 2012**	Education and Reminder	1	0	0	1	0	High	0	0	0	0	0	0	1	1	0	0	0	1
**180**	**Stockwell 2014**	Control	NA	NA	NA	NA	NA	NA	NA	NA	NA	NA	NA	NA	NA	NA	NA	NA	NA	NA
**180**	**Stockwell 2014**	Education	1	1	0	1	0	High	0	0	0	0	0	0	1	1	0	0	0	1
**176**	**Stockwell 2015**	Control	NA	NA	NA	NA	NA	NA	NA	NA	NA	NA	NA	NA	NA	NA	NA	NA	NA	NA
**176**	**Stockwell 2015**	Reminder	1	1	0	1	0	High	0	0	0	0	0	0	1	0	0	0	0	1
**176**	**Stockwell 2015**	Reminder	1	0	0	1	0	High	0	0	0	0	0	0	0	0	0	0	0	1
**179**	**Stockwell 2022**	Control	NA	NA	NA	NA	NA	NA	NA	NA	NA	NA	NA	NA	NA	NA	NA	NA	NA	NA
**179**	**Stockwell 2022**	Education and Reminder	1	1	0	1	0	High	1	0	0	0	0	0	1	0	0	0	0	1
**181**	**Stolpe 2019**	Control	NA	NA	NA	NA	NA	NA	NA	NA	NA	NA	NA	NA	NA	NA	NA	NA	NA	NA
**181**	**Stolpe 2019**	Education	1	0	0	1	0	Medium	0	1	0	0	0	0	0	1	0	0	0	0
**182**	**Strathdee 2023**	Control	NA	NA	NA	NA	NA	NA	NA	NA	NA	NA	NA	NA	NA	NA	NA	NA	NA	NA
**182**	**Strathdee 2023**	Education	1	1	1	0	1	Low	0	0	0	0	0	0	1	1	0	0	1	0
**183**	**Stuck 2015**	Control	NA	NA	NA	NA	NA	NA	NA	NA	NA	NA	NA	NA	NA	NA	NA	NA	NA	NA
**183**	**Stuck 2015**	Education	1	1	1	1	0	High	0	0	0	0	0	0	0	0	0	0	0	0
**184**	**Suh 2012**	Control	NA	NA	NA	NA	NA	NA	NA	NA	NA	NA	NA	NA	NA	NA	NA	NA	NA	NA
**184**	**Suh 2012**	Education and Reminder	1	0	0	1	0	Medium	0	0	0	0	0	0	0	0	0	0	0	1
**185**	**Suzuki, 2022**	Control	NA	NA	NA	NA	NA	NA	NA	NA	NA	NA	NA	NA	NA	NA	NA	NA	NA	NA
**185**	**Suzuki, 2022**	Education	0	0	0	1	0	Low	0	0	0	0	0	0	1	1	1	0	0	0
**186**	**Sweeney 2014**	Control	NA	NA	NA	NA	NA	NA	NA	NA	NA	NA	NA	NA	NA	NA	NA	NA	NA	NA
**186**	**Sweeney 2014**	Education	1	1	1	1	0	Low	0	0	0	0	0	0	1	1	1	0	0	0
**193**	**Szilagyi 2006**	Control	NA	NA	NA	NA	NA	NA	NA	NA	NA	NA	NA	NA	NA	NA	NA	NA	NA	NA
**193**	**Szilagyi 2006**	Reminder	1	0	0	1	0	High	0	0	0	0	0	0	0	0	0	0	0	1
**192**	**Szilagyi 2011**	Control	NA	NA	NA	NA	NA	NA	NA	NA	NA	NA	NA	NA	NA	NA	NA	NA	NA	NA
**192**	**Szilagyi 2011**	Multicomponent	1	1	1	0	1	High	0	0	0	0	0	1	0	0	0	0	0	1

	Study	Arm	PD	In	Hum	HC	Com	Int	EO	ASH	ASO	AD	Inc	CC	VS	DR	SF	DA	MI	Act
**189**	**Szilagyi 2013**	Control	NA	NA	NA	NA	NA	NA	NA	NA	NA	NA	NA	NA	NA	NA	NA	NA	NA	NA
**189**	**Szilagyi 2013**	Reminder	1	0	0	1	0	High	0	0	0	0	0	0	0	0	0	0	0	1
**189**	**Szilagyi 2013**	Reminder	1	0	0	1	0	High	0	0	0	0	0	0	0	0	0	0	0	1
**194**	**Szilagyi 2018**	Control	NA	NA	NA	NA	NA	NA	NA	NA	NA	NA	NA	NA	NA	NA	NA	NA	NA	NA
**194**	**Szilagyi 2018**	Access	1	0	0	1	0	High	1	0	0	0	0	0	0	0	0	0	0	1
**195**	**Szilagyi 2019**	Control	NA	NA	NA	NA	NA	NA	NA	NA	NA	NA	NA	NA	NA	NA	NA	NA	NA	NA
**195**	**Szilagyi 2019**	Reminder	1	0	0	1	0	High	0	0	0	0	0	0	0	0	0	0	0	1
**188**	**Szilagyi 2020**	Control	NA	NA	NA	NA	NA	NA	NA	NA	NA	NA	NA	NA	NA	NA	NA	NA	NA	NA
**188**	**Szilagyi 2020**	Education	1	0	0	1	0	Low	0	0	0	0	0	0	1	1	1	0	0	0
**188**	**Szilagyi 2020**	Education	1	0	0	1	0	Medium	0	0	0	0	0	0	1	1	1	0	0	0
**188**	**Szilagyi 2020**	Education	1	0	0	1	0	Medium	0	0	0	0	0	0	1	1	1	0	0	0
**190**	**Szilagyi 2020**	Control	NA	NA	NA	NA	NA	NA	NA	NA	NA	NA	NA	NA	NA	NA	NA	NA	NA	NA
**190**	**Szilagyi 2020**	Reminder	1	0	0	1	0	Medium	0	0	0	0	0	0	1	0	0	0	0	1
**190**	**Szilagyi 2020**	Reminder	1	0	0	1	0	Medium	0	0	0	0	0	0	1	0	0	0	0	1
**190**	**Szilagyi 2020**	Reminder	1	0	0	1	0	Medium	0	0	0	0	0	0	1	0	0	0	0	1
**187**	**Szilagyi 2020**	Control	NA	NA	NA	NA	NA	NA	NA	NA	NA	NA	NA	NA	NA	NA	NA	NA	NA	NA
**187**	**Szilagyi 2020**	Education and Reminder	1	0	0	1	0	Medium	0	0	0	0	0	0	1	0	0	0	0	1
**187**	**Szilagyi 2020**	Education and Reminder	1	0	0	1	0	High	0	0	0	0	0	0	1	0	0	0	0	1
**187**	**Szilagyi 2020**	Education and Reminder	1	0	0	1	0	High	0	0	0	0	0	0	1	0	0	0	0	1
**191**	**Szilagyi 2024**	Control	NA	NA	NA	NA	NA	NA	NA	NA	NA	NA	NA	NA	NA	NA	NA	NA	NA	NA
**191**	**Szilagyi 2024**	Reminder	0	0	0	1	0	High	0	0	0	0	0	0	0	0	0	0	0	1
**191**	**Szilagyi 2024**	Reminder	1	0	0	1	0	High	0	0	0	0	0	0	0	0	0	0	0	1
**191**	**Szilagyi 2024**	Control	NA	NA	NA	NA	NA	NA	NA	NA	NA	NA	NA	NA	NA	NA	NA	NA	NA	NA
**191**	**Szilagyi 2024**	Reminder	0	0	0	1	0	High	0	0	0	0	0	0	0	0	0	0	0	1
**191**	**Szilagyi 2024**	Reminder	1	0	0	1	0	High	0	0	0	0	0	0	0	0	0	0	0	1
**191**	**Szilagyi 2024**	Control	NA	NA	NA	NA	NA	NA	NA	NA	NA	NA	NA	NA	NA	NA	NA	NA	NA	NA
**191**	**Szilagyi 2024**	Reminder	0	0	0	1	0	High	0	0	0	0	0	0	0	0	0	0	0	1
**191**	**Szilagyi 2024**	Reminder	1	0	0	1	0	High	0	0	0	0	0	0	0	0	0	0	0	1

	Study	Arm	PD	In	Hum	HC	Com	Int	EO	ASH	ASO	AD	Inc	CC	VS	DR	SF	DA	MI	Act
**196**	**Tentori 2022**	Reminder	1	0	0	1	0	Low	0	0	0	0	0	0	0	0	0	0	0	1
**196**	**Tentori 2022**	Multicomponent	1	0	0	1	0	Low	0	1	0	0	0	0	0	0	0	0	0	1
**197**	**Terrell-Perica 2001**	Control	NA	NA	NA	NA	NA	NA	NA	NA	NA	NA	NA	NA	NA	NA	NA	NA	NA	NA
**197**	**Terrell-Perica 2001**	Reminder	1	0	0	1	0	Low	0	0	0	0	0	0	0	0	0	0	0	1
**197**	**Terrell-Perica 2001**	Reminder	1	0	0	1	0	Low	0	0	0	0	0	0	0	0	0	0	0	1
**198**	**Thilly, 2024**	Control	NA	NA	NA	NA	NA	NA	NA	NA	NA	NA	NA	NA	NA	NA	NA	NA	NA	NA
**198**	**Thilly, 2024**	Multicomponent	1	1	1	0	1	Medium	1	0	0	0	0	0	0	0	0	0	0	0
**198**	**Thilly, 2024**	Education	1	1	1	0	1	Low	0	0	0	0	0	0	0	0	0	0	0	0
**199**	**Tiro 2015**	Education and Reminder	1	1	1	1	0	High	0	1	0	0	0	0	1	0	0	0	0	1
**199**	**Tiro 2015**	Reminder	1	1	1	1	0	Medium	0	0	0	0	0	0	0	0	0	0	0	1
**200**	**Topp 2013**	Control	NA	NA	NA	NA	NA	NA	NA	NA	NA	NA	NA	NA	NA	NA	NA	NA	NA	NA
**200**	**Topp 2013**	Affordability	1	0	0	1	0	Low	0	0	0	0	1	0	0	0	0	0	0	0
**201**	**Tubiana 2021**	Education and Reminder	1	1	1	1	0	High	0	0	0	0	0	0	1	1	0	0	0	1
**201**	**Tubiana 2021**	Education	1	1	1	1	0	Medium	0	0	0	0	0	0	1	1	0	0	0	0
**202**	**Tull 2019**	Control	NA	NA	NA	NA	NA	NA	NA	NA	NA	NA	NA	NA	NA	NA	NA	NA	NA	NA
**202**	**Tull 2019**	Education and Reminder	1	0	0	1	0	Low	0	0	0	0	0	0	0	1	0	0	0	1
**202**	**Tull 2019**	Reminder	1	0	0	1	0	Low	0	0	0	0	0	0	0	0	0	0	0	1
**203**	**Ueberroth 2022**	Control	NA	NA	NA	NA	NA	NA	NA	NA	NA	NA	NA	NA	NA	NA	NA	NA	NA	NA
**203**	**Ueberroth 2022**	Reminder	0	0	0	1	0	Medium	0	0	0	0	0	0	0	0	0	0	0	1
**203**	**Ueberroth 2022**	Reminder	0	0	0	1	0	Medium	0	0	1	0	0	0	0	0	0	0	0	1
**204**	**Usami 2009**	Control	NA	NA	NA	NA	NA	NA	NA	NA	NA	NA	NA	NA	NA	NA	NA	NA	NA	NA
**204**	**Usami 2009**	Education	1	1	1	1	0	Low	0	0	0	0	0	0	1	1	1	0	0	0
**205**	**Vanderpool 2013**	Control	NA	NA	NA	NA	NA	NA	NA	NA	NA	NA	NA	NA	NA	NA	NA	NA	NA	NA
**205**	**Vanderpool 2013**	Education and Reminder	1	0	0	1	0	Medium	0	0	0	0	0	0	1	1	1	0	0	1
**206**	**Viver 2000**	Control	NA	NA	NA	NA	NA	NA	NA	NA	NA	NA	NA	NA	NA	NA	NA	NA	NA	NA
**206**	**Viver 2000**	Reminder	1	1	1	1	0	Low	0	1	0	0	0	0	0	0	0	0	0	1
**206**	**Viver 2000**	Reminder	1	0	0	1	0	Low	0	0	0	0	0	0	0	0	0	0	0	1
**206**	**Viver 2000**	Reminder	1	1	1	1	0	Medium	0	1	0	0	0	0	0	0	0	0	0	1

	Study	Arm	PD	In	Hum	HC	Com	Int	EO	ASH	ASO	AD	Inc	CC	VS	DR	SF	DA	MI	Act
**207**	**Wang 2021**	Control	NA	NA	NA	NA	NA	NA	NA	NA	NA	NA	NA	NA	NA	NA	NA	NA	NA	NA
**207**	**Wang 2021**	Education	1	1	1	1	1	High	0	0	0	0	0	0	1	1	1	0	1	0
**207**	**Wang 2021**	Education	0	1	1	1	1	High	0	1	0	0	0	0	1	1	1	0	0	0
**208**	**Wang 2023**	Control	NA	NA	NA	NA	NA	NA	NA	NA	NA	NA	NA	NA	NA	NA	NA	NA	NA	NA
**208**	**Wang 2023**	Education	0	0	0	1	0	Medium	0	0	0	0	0	0	1	1	1	0	0	0
**209**	**Weaver 2014**	Control	NA	NA	NA	NA	NA	NA	NA	NA	NA	NA	NA	NA	NA	NA	NA	NA	NA	NA
**209**	**Weaver 2014**	Affordability	1	1	1	1	0	Medium	0	0	0	0	1	0	0	0	0	0	0	0
**209**	**Weaver 2014**	Affordability	1	1	1	1	0	Medium	0	0	0	0	1	0	0	0	0	0	0	0
**210**	**Wijesundara 2020**	Control	NA	NA	NA	NA	NA	NA	NA	NA	NA	NA	NA	NA	NA	NA	NA	NA	NA	NA
**210**	**Wijesundara 2020**	Education	0	0	0	1	0	Low	0	0	1	0	0	0	1	1	1	0	0	0
**210**	**Wijesundara 2020**	Control	NA	NA	NA	NA	NA	NA	NA	NA	NA	NA	NA	NA	NA	NA	NA	NA	NA	NA
**210**	**Wijesundara 2020**	Education	1	1	0	1	0	Low	1	0	0	0	0	0	1	1	1	0	0	0
**211**	**Winston 2007**	Control	NA	NA	NA	NA	NA	NA	NA	NA	NA	NA	NA	NA	NA	NA	NA	NA	NA	NA
**211**	**Winston 2007**	Reminder	1	1	1	1	0	Medium	0	1	0	0	0	0	0	0	0	0	0	1
**212**	**Wiseman 2016**	Control	NA	NA	NA	NA	NA	NA	NA	NA	NA	NA	NA	NA	NA	NA	NA	NA	NA	NA
**212**	**Wiseman 2016**	Education and Reminder	1	0	0	1	0	High	0	0	0	0	0	0	1	1	1	0	0	1
**213**	**Wong 2016**	Control	NA	NA	NA	NA	NA	NA	NA	NA	NA	NA	NA	NA	NA	NA	NA	NA	NA	NA
**213**	**Wong 2016**	Education	1	1	1	1	0	Low	0	0	0	0	0	0	1	1	0	0	0	0
**214**	**Wouters, 2007**	Control	NA	NA	NA	NA	NA	NA	NA	NA	NA	NA	NA	NA	NA	NA	NA	NA	NA	NA
**214**	**Wouters, 2007**	Access	1	0	0	1	0	Medium	0	0	0	1	0	0	0	0	0	0	0	0
**215**	**Wright 2012**	Control	NA	NA	NA	NA	NA	NA	NA	NA	NA	NA	NA	NA	NA	NA	NA	NA	NA	NA
**215**	**Wright 2012**	Reminder	0	0	0	1	0	Low	0	0	0	0	0	0	0	0	0	0	0	1
**216**	**Wynn 2021**	Reminder	1	0	0	1	0	High	0	0	0	0	0	0	0	0	0	0	0	1
**216**	**Wynn 2021**	Education and Reminder	1	1	0	1	0	High	0	0	0	0	0	0	1	0	0	0	0	1
**217**	**Xu 2022**	Control	NA	NA	NA	NA	NA	NA	NA	NA	NA	NA	NA	NA	NA	NA	NA	NA	NA	NA
**217**	**Xu 2022**	Education and Reminder	0	1	1	1	0	Low	0	0	1	0	0	0	1	0	0	0	0	1
**218**	**Yeung 2018**	Control	NA	NA	NA	NA	NA	NA	NA	NA	NA	NA	NA	NA	NA	NA	NA	NA	NA	NA
**218**	**Yeung 2018**	Multicomponent	1	1	1	1	0	High	0	0	0	0	0	1	1	1	0	0	0	1

	Study	Arm	PD	In	Hum	HC	Com	Int	EO	ASH	ASO	AD	Inc	CC	VS	DR	SF	DA	MI	Act
**219**	**Yokum 2018**	Control	NA	NA	NA	NA	NA	NA	NA	NA	NA	NA	NA	NA	NA	NA	NA	NA	NA	NA
**219**	**Yokum 2018**	Education and Reminder	1	0	0	1	0	Low	0	0	0	0	0	0	0	1	1	0	0	1
**219**	**Yokum 2018**	Education and Reminder	1	0	0	1	0	Low	0	0	0	0	0	0	0	1	1	0	0	1
**219**	**Yokum 2018**	Education and Reminder	1	0	0	1	0	Low	0	0	0	0	0	0	0	1	1	0	0	1
**219**	**Yokum 2018**	Education and Reminder	1	0	0	1	0	Low	0	0	0	0	0	0	0	1	1	0	0	1
**220**	**Yudin 2016**	Control	NA	NA	NA	NA	NA	NA	NA	NA	NA	NA	NA	NA	NA	NA	NA	NA	NA	NA
**220**	**Yudin 2016**	Education and Reminder	1	0	0	1	0	High	0	0	0	0	0	0	1	1	1	0	0	1
**221**	**Zhang 2018a**	Control	NA	NA	NA	NA	NA	NA	NA	NA	NA	NA	NA	NA	NA	NA	NA	NA	NA	NA
**221**	**Zhang 2018a**	Education	1	1	1	1	0	High	0	0	0	0	0	0	1	1	1	0	0	0
**221**	**Zhang 2018a**	Education	1	1	1	1	0	High	0	0	0	0	0	0	1	1	1	0	0	0
**222**	**Zhang 2018b**	Education	1	1	1	1	1	High	0	0	0	0	0	1	0	0	0	0	0	0
**222**	**Zhang 2018b**	Education	1	1	1	1	0	Low	0	0	0	0	0	1	0	0	0	0	0	0
**223**	**Zhang 2022**	Control	NA	NA	NA	NA	NA	NA	NA	NA	NA	NA	NA	NA	NA	NA	NA	NA	NA	NA
**223**	**Zhang 2022**	Education	0	0	0	1	0	High	0	0	0	0	0	0	1	1	1	0	0	0
**224**	**Zuniga de Nuncio 2003**	Control	NA	NA	NA	NA	NA	NA	NA	NA	NA	NA	NA	NA	NA	NA	NA	NA	NA	NA
**224**	**Zuniga de Nuncio 2003**	Education and Reminder	1	1	1	1	0	Medium	0	0	0	0	0	0	0	1	0	0	0	1

Footnotes

PD: Personal Delivery; **In:** Interaction; **Hum:** Human interaction; **HC:** Delivered in part by healthcare professionals; **Com:** delivered in part by community members; **Int:** Intensity; **EO:** Access-Extended Opportunities; **ASH:** Access-Appointment Scheduling Help; **ASO:** Access – Appointment Scheduling Online; **AD:** Access – Accelerated Dosing; **Inc:** Affordability – Incentives; **CC:** Affordability – Costs Covered; **VS:** Acceptance – Vaccine Safety/efficacy; **DR:** Acceptance – Disease Perceived Risk; **SF:** Acceptance – Social Factors; **DA:** Acceptance – Decision Aids; **MI:** Acceptance – Motivational Interviewing; **Act:** Activation.
'Arm' relates to the broad category of intervention assigned to the arm and used in our network meta-analysis of broad categories Davies S, Davies, AL., Higgins, JPT, Caldwell, DM., Thornton, ZA., Aiton, E, Ali, I, Dawson, S, McGrath, C, Parkhouse, T, Yardley, L, Yates, J, Letley, L, Ismail, S, French, CE. Recipient-focused interventions to increase vaccine uptake in high and upper-middle income countries: a systematic review and network meta-analysis *eClinicalMedicine* 2025; **90**.

H: Socio-economic data from studies

	Study	Religion	Education level	Socio economic data
1	Abroms 2023	Not reported	(N = 478): High school or less n = 104 (21.8%), Associate's degree/some college n = 234 (49.0%), and Bachelor's/graduate degree n = 140 (29.3%).	Income: Less than US$20,000: n = 82 (17.2%), US$20,000 to US$49,999: n = 193 (40.4%), More than US$50,000: n = 198 (41.4%) and Prefer not to answer: n = 5 (1.0%). Employment: Working: n = 341 (71.3%).
2	Alonge 2023	Not reported	Not reported	(N=4296): Has public health insurance n = 2502 (58.2%)
3	Anraad 2023	Affiliation with religion (1 = no affiliation – 7 = strong affiliation) - Mean (standard deviation): control = 2.13 (1.33) and intervention = 2.06 (1.29)	Highest education completed (N=1236): low (less than secondary or vocational education) n = 6 (0.5), intermediate (secondary and vocational education) n = 265 (21.4%), and high (higher or university education) n = 965 (78.1%)	Not reported
4	Arnold 2022	Not reported	Not reported	Not reported
5	Arthur 2002	Not reported	Not reported	Not reported
6	Bartos 2022	Not reported	Full sample used (N = 2,101): primary 4.6%, lower secondary 27.7%, upper secondary 36.3%, and university 31.5%	Household income: Up to 10,000 CZK= 1.4%, 10,001 - 15,000 CZK =6.5%, 15,001 - 20,000 CZK =9.5%, 20,001 - 25,000 CZK =7.5%, 25,001 - 30,000 CZK =10.8%, 30,001 - 35,000 CZK =12.3%, 35,001 - 40,000 CZK =10.9%, 40,001 - 50,000 CZK =12.2%, 50,001 - 60,000 CZK =9.0%, Over 60,000 CZK= 8.5%, and I don't know / Don't want to say 11.5%. Economic status: Employee 48.0%, Entrepreneur 4.6%, Student 3.5%, Parental leave 3.9%, Retired 34.8%, Unemployed 3.6%, and Other 1.6%.
7	Bartu 2006	Not reported	Education (N=152): High school not completed: 41% (63/152); High school completed: 15% (23/152); Technical college: 18% (27/152); University 2% (3/152); Other (trade, apprenticeship, professional registration): 21% (32/152)	Total income in previous year (n = 147): 70% (103/147) Less than $A20 000, 26% (38/147) $A20 000–40 000 , 4% (6/147) More than $A40 000; Employed (n = 147): 22% (32/147) Full time, 30% (45/147) part time/causal, and 48% (70/147) not employed
8	Baskin 2018	Not reported	All participants in this study (N = 30,748) were students, faculty and staff at a large university in the United States; 18% undergraduate students,	Not reported

	Study	Religion	Education level	Socio economic data
			23% were graduate and professional students, and rest is various employees from high school degree to being close to retirement.	
9	Bastani 2022	Not reported	Grade school n = 88 (37%), High school diploma n = 89 (37.4%), college n = 55 (23.1%), Post college n = 6 (2.5%)	Annual household income (N = 238): <$12,000: n = 64 (26.9%), $12,000–<$24,000: n = 123 (51.7%), $24,000–$36,000: n = 35 (14.7%), $36,000: n = 13 (5.5%), Other: n = 1 (0.4%), and Do not know/refuse to answer: n = 2 (0.8%). Insurance status: Medi-Cal or Healthy Families: n = 196 (82.3%), Other insurance: n = 14 (5.9%), and Uninsured: n =28 (11.8%).
10	Bennett 2015	Not reported	Student standing (N = 661): Undergraduate n = 445 (67.3%), Graduate n = 182 (27.5%), and Professional n = 34 (5.1%)	Employment: Full-time: n = 55 (8.3%), Part-time: n = 323 (48.9%), Unemployed: n = 280 (42.4%), and Missing: n = 3 (0.5%). Health insurance: UM SHIP or GradCare: n = 106 (16.0%), Parent's private: n = 438 (66.3%), Medicaid: n = 10(1.5), Other insurance: n = 41 (6.2%), None: n = 43 (6.5%), and Don't know/missing: n = 23 (3.5%).
12	Berg 2004	Not reported	Not reported	Not reported
11	Berg 2008	Not reported	Not reported	Not reported
13	Berkhout 2018	Not reported	Not reported	Not reported
14	Bernard-Genest 2021	Not reported	N = 127: High school diploma n = 30 (23.6%), college n = 39 (30.7%), Bachelor's degree n = 36 (28.3%), Master's degree n = 21 (16.5%), and PhD n = 1 (0.8%)	Not reported
15	Berset 2022	Not reported	Not reported	Insurance (N = 945): Public (i.e., Medicaid): n = 807 (85.4%): Private: n = 112 (11.9%), and self-pay: n = 26 (2.8%). The study population included predominantly non-Hispanic Black, low-income children (age, 6-17 years).
16	Berset 2023	Not reported	Not reported	Insurance (N = 1312): Public (i.e., Medicaid): n = 1140 (86.9%), Private: n = 50 (3.8%), Self-pay: n = 119 (9.1%), and Missing data: n = 3 (0.2%). The study was conducted in academic primary care practices serving low-income, predominantly Black patients
17	Bethke 2024	Not reported	**Students** (N = 6512): 1424 (21.9%) attended high schools, 1714 (26.3%)	Employment status of parents (N = 6310): Both unemployed: n = 451 (7.1%), One employed: n =

	Study	Religion	Education level	Socio economic data
			integrated secondary schools, and 3374 (51.8%) vocational schools. **Education mother (N = 4537)**: No education degree = 236 (5.2%), Elementary – Primary school degree= 342 (7.5%), Secondary school degree= 1558 (34.3%), High school degree= 1049 (23.1%), and University degree = 1352 (29.8%). **Education father (N = 4154)**: No education degree= 230 (5.5%), Elementary – Primary school degree = 341 (8.2%), Secondary school degree=1323 (31.8%), High school degree = 848 (20.4%), and University degree = 1412 (34%).	1775 (28.1%), and Both employed: n = 4084 (64.7%),
18	Bian 2023	Not reported	College students (N = 202): Junior college student/undergraduate student 177 (87.6%) and Graduate student 25 (12.4%). Grandparent (N = 375): Junior high school and below 320 (85.3%) and High school and above 55 (14.7%).	Not reported
19	Borg 2018	Not reported	Not reported	There are data for socio-economic index for areas (SEIFA) quintile for each groups, but overall percentage not clearly reported: around 30% of children in each group lived in the most disadvantaged socio-economic quintile area in Victoria.
20	Bourgeois 2008	Not reported	Not reported	All are employees.
21	Bowman, 2014	Not reported	Not reported	Not reported
22	Brigham 2012	Not reported	Not reported	Insurance type (N = 420): - Private: n = 220 (52.4%), Public: n = 190 (45.2%), None: n = 10 (2.4%)
23	Bronchetti 2015	Not reported	The study was conducted at small colleges (N = 9356)	Zip code median income (Means by treatment group): 9.015 (3.560) for control, 8.964 (3.621) for incentive, 9.836 (3.521) for peer, and 9.095 (3.627) for coughing. Note: Sample sizes for means for zip code median income are 2168, 2171, 2172, and 2154, respectively, because zip

	Study	Religion	Education level	Socio economic data
				codes are missing for international students.
24	Burkhardt 2023	Not reported	Not reported	Insurance (N = 1235): Public (i.e., Medicaid): n = 1090 (88.3%), Private: n =121 (9.8%), self-pay: n = 24(1.9%). The study took place at 3 academic paediatric primary care practices. These practices serve a predominantly non-Hispanic Black, low-income population.
25	Buttenheim 2022	Not reported	Not reported	Not reported
26	Campos-Mercade 2021	Not reported	Education (N = 8286): 2% Elementary School or Lower, 30% High-school,13% Professional Training, 7% In College, 46% College Degree, and 2% PhD	Average monthly income (SEK) = 24,847: 3% Income 0-5000kr,5% Income 5001-10000kr, 11% Income 10001-15000kr,11% Income 15001-20000kr, 22% Income 20001-25000kr, 20% Income 25001-30000kr, 13% Income 30001-35000kr, 7% Income 35001-40000kr,4% Income 35001-40000kr, Income 45001-50000kr 2%, 1% Income 50000kr-55000kr, and 1% Income more than 55000kr. Occupation: 81% Employed, 4% Unemployed, 11 % in college, 1% Retired, and 4% Other Professional situations
27	Cataldi 2024	Not reported	Not reported	Not reported
28	Centers for Disease 2012	Not reported	Not reported	Not reported
29	Chai 2013	Not reported	Total (N = 1992): Primary school or below: 42 (2.1%), Junior high: 288 (14.5%), Senior high or secondary school: 788 (39.6%), Junior or full college degree: 842 (42.3%), Graduate degree and above: 32 (1.6%)	Not reported
30	Chang 2023	Not reported	Not reported	Insurance (N = 57,893): Commercial (2.7%), medical (74.7%), Medicare (12.8%), other (5.7%), and Uninsured (4.1%). The group targeted by the study was a racially/ethnically diverse, primarily low-income adult population.
31	Chao 2015	Not reported	Not reported	Not reported
32	Chodick 2021	Not reported	Not reported	Socioeconomic level (SES): 23% (5013/21979) Lowest, 32% (6996/21979) Q2, 18% (4099/21979) Q3, and 27% (5871/21979) Highest; Median (IQR) socio-economic status [tool used to measure SES not stated]: 6 (4-11).

	Study	Religion	Education level	Socio economic data
33	Clayton 2021a	Not reported	Wave 1 (N = 2332): Less than high school diploma 1.7%, High school diploma 11.9%, Some college 13.8%, Associate's degree 7.4%, Bachelor's degree 26.4%, Some professional or graduate school but no degree 5.8%, and Professional or graduate degree 32.9%. Less than high school diploma: 7 (1%), High school diploma: 74 (10.9%), Some college: 105 (15.5%), Associate's degree: 55 (8.1%), Bachelor's degree: 181 (26.7%), Some professional or graduate school but no degree: 49 (7.2%), Professional or graduate degree: 207 (30.5%).	Medicaid: Yes 33.4%, No 66.6%. SCHP: Yes 38.9%, No 61.1%.
33	Clayton 2021b	See Clayton 2021a	Not reported	
34	Coley 2018	Not reported	Not reported	Not reported
35	Conner 2017	Not reported	Not reported	Sample mainly lived in areas of low deprivation (Townsend score M = -1.47, SD = 2.93).
36	Cox 2012	Not reported	High school diploma or more n = 889 (76.5%)	Employed = 624/1175 (53.1%). Income < $10K = 501/1175 (42.6%).
37	Cutrona 2018	Not reported	Not reported	Not reported
38	Dai 2021a	Not reported	Not reported	Not reported
38	Dai 2021b	See Dai 2021a	Not reported	
39	Dalby 2000	Not reported	Not reported	Not reported
41	Daley 2002	Not reported	Not reported	Not reported
40	Daley 2014	Not reported	All are primary school students (sample of kindergarten to eighth-grade schools).	Percent of student body eligible for free or reduced lunch in intervention schools (min-max): median = 67% (17, 91); in control schools median = 61% (5, 93).
42	Daniels 2007	Not specifically reported, however study was conducted in a community church setting.	Not reported	Income less or equal to $30,000: n = 92 (65%). Income more than or equal to $30,000: n = 50 (35%). Health Insurance Status: Insured: n = 127 (71%); Uninsured: n = 53 (29%).
43	Dapp 2011	Not reported	Not reported	Not reported
44	Davies 2017	Not reported	All are secondary school students.	Not reported
45	DeCamp 2020	Not reported	Maternal education (n = 157): Eighth grade or less= 64 (40.8%), Some high school = 41 (26.1%), and	Annual family income: <$20 000: n = 67 (42.7%), $20 000–$30 000: n = 38 (24.2%), >$30 000: n

	Study	Religion	Education level	Socio economic data
			High school or greater = 52 (33.1%).	= 12 (7.6%), and Did not report or unknown: n = 40 (25.5%)
46	Dempsey 2019	Not reported	Not reported	Not reported
47	DiClemente 2015	Not reported	Education: Less than 8th grade n = 13 (6.0%), 8th grade n = 25 (11.6%), 9th grade n = 44 (20.4), 10th grade n = 30 (13.9%), 11th grade n = 42 (19.4%), 12th grade n = 28 (13.0%), High school grad or GED n = 34 (15.7%)	Received Public Assistance: No n = 113 (52.3%), Welfare (TANF, SSI) n = 18 (8.3%), Food stamps n = 99 (45.8%), WIC N = 26 (12.0%), Section 8 housing n = 10 (4.6%); Currently Employed n = 34 (15.7%); Health Insurance: Private n = 19 (8.8%), Medical n = 97 (44.9%), GA CHIP n = 3 (1.4%), No insurance n = 38 (17.6%), and Don't know n = 59 (27.3%)
48	Dini 2000	Not reported	Not reported	Not reported
49	Dombkowski 2014	Not reported	Not reported	Not reported
50	Dombkowski 2017a	Not reported	Not reported	Medicaid enrolment status (N = 1497): Never enrolled n = 997(66.6%), Previously enrolled n = 121(8.1%), Currently enrolled n = 313(20.9%), and Unknown enrolment status n = 66(4.4%)
50	Dombkowski 2017b	See Dombkowski 2017a	See Dombkowski 2017a	See Dombkowski 2017a
51	Domek 2019	Not reported	Mother’s education (n = 720): No education 10 (1.4%), Completed or some primary education 184 (25.6%), Completed or some secondary education 173 (24.0%), Completed or some higher education 353(49.0%). Father’s education (n = 720): No education 9 (1.5%), Completed or some primary education 103 (17.1%), Completed or some secondary education 144 (24.0%), Completed or some higher education 345 (57.4%).	Family monthly income level: <=Q1000: n = 207 (28.8%), Q1001-2000: n =155 (21.5%), Q2001-3000: n = 166 (23.1%), Q3001-4000: n = 103 (14.3%), >=Q4001: n = 89 (12.4%).
52	Doyle 2015	Not reported	Low education = 75/205 (36.6%)	Employed = 78 (38%)
53	El-Mohandes, 2003	Not reported	Less than high school n = 128 (44.8%), High School n = 126 (44.1%), Above high school n = 13 (8.9%)	Below poverty level: n = 172 (60.1%), At least 1 household member receiving Medicaid: n = 227 (79.4%), At least 1 household member receiving: WIC n = 119 (41.6%), and Employment (working at time of pregnancy): n = 114 (39.9%). WIC indicates the Special Supplemental Nutrition Program for Women, Infants, and Children.

	Study	Religion	Education level	Socio economic data
54	Esposito 2018	Not reported	Not reported	Not reported
55	Fernandez 2022	Not reported	Less than high school n = 374 (24.2%), High school or GED n = 706 (45.6), Post high school n = 468 (30.2%).	Income: <$10,000 n = 717 (48.6%), $10,000-$20,000: n = 537 (36.4%), and >=$20,000: n = 220 (14.9%). Insurance: No insurance (or CHIP only): n = 850 (54.8%), Public and/or private insurance: n = 700 (45.2%)
56	Ferreira 2022	47.5% of the CG considered themselves Catholic and 43.2% of the IG reported being Evangelical. Christian: 104 (65.4%), Other: 54 (34.0%)	Not reported	most participants in both groups had an income of up to USD 363.63 (Control = 84.2%; Intervention = 68.6%).
57	Fiks 2013	Not reported	Not reported	Insurance status: Private n = 17903 (80%), Nonprivate n = 4583 (20%).
58	Fitzpatrick 2018	Not reported	High school or below: 128 (23.0%), Technical school: 181 (32.6%), College: 219 (39.4%), Advanced degree: 29 (5.0%).	Monthly income (USD). <220: n = 149 (26.8%), 220-439: n = 131 (23.6%), 440-732: n = 168 (30.2%), 733-1171: n = 78 (14.0%), and >1172: n = 30 (5.4%). Occupation: student: n = 180 (32.4%), Office worker/white collar: n = 123 (22.1%), service/retail: n = 106 (19.1%), and other: n = 147 (26.4%)
59	Frew 2016	Not reported	Total sample= 95; Less than high school n = 12 (13%), High school graduate or equivalent (GED) n = 45 (47%), Technical/vocational or associates n = 29 (31%), Bachelor degree n = 8 (8%), and Graduate degree n = 1 (1%)	Currently has health insurance yes n = 87/95 (92%)
60	Gerend 2021	Not reported	N = 147; Some high school/high school degree/GED= 42 (28.6%), Some college or trade school certificate= 56 (38.1%), College degree= 31(21.1%), and Some graduate school/ graduate degree= 18 (12.2%)	Annual family income: <$20,000: n = 25 (18.4%), $20,000–$39,999: n = 35 (25.7%), $40,000–$59,999: n = 24 (17.6%), $60,000–$79,999: n = 25 (18.4%), ≥$80,000: n = 27 (19.8%). Health Insurance: None: n = 21 (14.4%), parents' insurance: n = 62 (42.5%), personal insurance: n = 63 (43.1%)
61	Glanz 2017	Not reported	Some college or less: 186 (17.0%); College or higher 905 (82.8%).	Household income equal to $ 80,000 or less: n = 440 (40.3%): More than $80,000: n = 597 (54.6)
62	Glanz 2020	Not reported	Grade school= 6 (0.7%), High school= 19 (2.3%),	Household income:< $40 000: n = 59 (7.16%), $40 000–$80 000: n

	Study	Religion	Education level	Socio economic data
			Some college= 92 (11.2%), College= 323 (39.2%), Graduate school= 380 (46.1%), Nonresponse= 4 (0.5%)	= 222 (26.94%), $81 000–$120 000: n = 299 (36.29%), $121 000–$150 000: n = 83 (10.07%), $150,000: n =129 (15.7%), Nonresponse: n = 32 (3.9%). Employment: Employed full-time: n = 567 (68.81%), Employed part-time: n = 112 (13.59%), Unemployed: n = 13 (1.58%), Stay-at-home parent: n = 123 (14.93%), Student: n = 8 (0.97%), and Nonresponse: n = 1 (0.12%)
63	Goodman 2015	Not reported	Total Sample= 105--> 52 control and 53 intervention; Some high school 3.8% (~2/52) in control and 1.9% (~1/53) in the intervention= 3/105 (2.9%), High school 3.8% (~2/52) in control and 11.3% (~6/53) in the intervention= 8/105 (7.6%), some college 15.4% (~8/52) in the control and 25.4% (~13/53) in the intervention= 21/105 (20%), college 42.3 (~22/52) in the control and 37.7% (~20/53) in the intervention= 42/105 (40%), and post college 34.6% (~18/52) in the control and 22.6% (~12/53) in the intervention=30/105 (28.6%).	94% had private medical insurance (n = 105), > 86.5% in Control (~45/52) and 92.5% (~49/53) in the intervention.
64	Goodyear-Smith 2012	Not reported	Not reported	Not reported
65	Grandahl 2016	Not reported	Mother Education level: University 54.3% (325/598), Upper secondary school 39.5% (236/598), and elementary school 6.2% (37/598); Father Education level: University 38.1% (212/557), Upper secondary school 51.5% (287/557), and elementary school 10.4% (58//557)	Main occupation Mother's: Employed: 88.7% (654/737), Unemployed: 11.3% (83/737); Main occupation father: Employed: 94% (649/691), Unemployed: 6% (42/691). Employed includes studying and/or parental leave. Unemployed includes sick leave and similar.
67	Haff 2023	Not reported	Not reported	Not reported
68	Hambidge 2009	Not reported	Not reported	>99% with public insurance or uninsured). Self-pay at delivery 62/807 (7.7%).
69	Hanley 2023	Not reported	Not reported	Insurance (N = 7408): Medicaid: n = 2654 (35.8%), Private

	Study	Religion	Education level	Socio economic data
				(managed care): n = 4063 (54.8%), uninsured: n = 629 (8.5%), and other: n = 62 (0.1%).
70	Hannan 2013	Not reported	High school education (n = 96, 69.1%)	Sample size (N = 139): annual income of less than $20,000/year (n = 100, 71.9%). Most mothers were not employed (n = 76, 55.5%) and most were Medicaid recipients or awaiting coverage by Medicaid (n = 120, 76.3%).
71	Harari 2008	Not reported	Not reported	Not reported
72	Henrikson 2018	Not reported	Not reported	Not reported
73	Hess 2013	Not reported	Not reported.	Not reported
74	Higginbotham 2012	Not reported	Not reported.	Not reported
75	Ho 2019	Not reported.	Not reported.	Not reported
76	Hofstetter 2015	Not reported	Not reported	Insurance (N = 2054): Public: n = 1721 (83.8%), Private: n = 76 (3.7%), and uninsured: n = 257(12.5%)
77	Hofstetter 2015a	Not reported	Not reported	Insurance: public 88.5% (4833/5462), Private 5.9% (322/5462), uninsured 5.6% (307/5462). Studies subjects are among urban, low-income, minority children who remained unvaccinated in the late fall.
79	Howell-Jones 2023a	Religious denomination of the school (n (%)): Catholic 209 (6.98%), Church of England 895 (29.89%), Other Christian Faith 3 (0.10%), Sikh 3 (0.10%), Other 3 (0.10%), and No religion 1881 (62.83%).	Not reported	Deprivation (n (%)): High: n = 350 (11.69%), Low: n = 2644 (88.31%). % Eligible for free school meals in the school (mean (SD)) 12.04 (9.51%)
79	Howell-Jones 2023b	See Howell-Jones 2023a	Not reported	
81	Hu 2017	Not reported	Education level (N = 1252): Junior high school or less: 119 (9.5%), Senior high school or technical school: 378 (30.2%), College or above: 755 (60.3%)	Socioeconomic development levels. High: n = 411 (32.8%), Middle: n = 416 (33.2%), Low: n = 425 (33.9%). Occupation: n = No job 113 (9.0%), Farmer/worker/businessman: n = 827(66.0%), Civil servants: n = 239(31.3%), and Medical staff: n = 73 (5.8%)

	Study	Religion	Education level	Socio economic data
82	Hu 2018	Not reported	Maternal education level (n = 200): ≤ Primary school: 17 (8.5%), Middle school graduated: 65 (32.5%), Vocational or college graduated: 118 (59.0%).	Employed: n = 151 (75.5%), Unemployed: n = 49 (24.5%).
80	Hu 2021	Not reported	No formal education/primary 153 (47.8%), Secondary level 98 (30.6%), and Tertiary level 69 (21.6%)	Not reported
83	Huf 2024	Not reported	Not reported	Index of multiple deprivation (IMD) (mean, SD): for trial arm1= 4.3, 2.0; for trial arm 2 =4.3, 2.1; and for trial arm 3= 4.3, 2.0.
84	Hull 2002	Not reported	Not reported	Not reported
85	Humiston 2014a	Not reported	Not reported	Not reported
85	Humiston 2014b	See Humiston 2014a	See Humiston 2014a	See Humiston 2014a
86	Hurley 2018a	Not reported	Not reported	Insurance (N = 47268): Medicaid: n = 25424 (53.8%), Uninsured: n = 8705 (18.4%), Commercial: n = 3705 (7.8%), and Medicare: n = 8303(17.6%)
87	Hurley 2018b	See Hurley 2018a	Not reported	
88	Hurley 2019	Not reported	Not reported	Not reported
89	Hurtaud 2023	Not reported	Not reported	Not reported
90	Hwang 2010a	Not reported	< High school 70 (6%), High school 898 (71%), Some college 292 (23%)	Housing status: Permanent 52 (4%) and Temporary/streets 1208 (96%).
90	Hwang 2010b	See Hwang 2010a	See Hwang 2010a	See Hwang 2010a
91	Irigoyen 2006	Not reported	Not reported	Not reported
92	Isrctn, 2021	Not reported	Not reported	Not reported
93	Jackson 2011	Not reported	Left school at 16 years 34.5% (49/142), Left school at 18 years 14.1% (20/142), and Achieved Degree or higher 54.4% (73/142)	Mean Low Income Scheme Index score: 14.8% (21/142). Low Income Scheme Index score is based on the percentage of prescribed items exempt from a prescription charge due to low income of the patient
94	Janitz 2023	Not reported	Not reported	Not reported
95	Jiang 2022	Not reported	N = 350: Primary school or below 126 (36%), High school 164 (46.9%), and College or above 60 (17.1%)	Monthly income (Chinese Yuan): ≤1,000 =176 (50.3%), 1,000-4,000: n = 116 (33.1%), and ≥4,000: n = 58 (16.6%). Occupation: Retirement: n = 160 (45.7%), Full-time/part-time job: n = 33 (9.4%), and other: n = 157(44.9%)
96	Johansen 2023	Not reported	Not reported	Not reported
97	Johnson 2003	Not reported	Not reported	Not reported

	Study	Religion	Education level	Socio economic data
98	Jordan 2015	Not reported	Not reported	Poverty group: <=20% poverty 66.8% (4513/6758), > 20% poverty 33.2% (2245/6758). Poverty group (<20% poverty, >20% poverty) was assigned by matching participant's ZIP code with ZIP code–level poverty data available through the American Community Survey (2011).
99	Joseph 2016	Practice religion (N=152), Catholic 19% (36/193), Protestant 43% 84/193), N/A(not available) 21% (40/193), Other 17% (33/193)	Maternal highest level of education: Less than/some high school 21% (41/197), Completed high school 35% (67/193), College 41% (80/193), Master's and beyond 3% (5/193)	Average household income <$20 000: 45% (77/171); $200 000-35 000: 29% (50/171); $35 000-60 000: 19% (33/171); >$60 000: 6% (11/171)
100	Ju, 2024	Not reported	N = 1258: Junior high school and below n = 21 (1.7%), Senior high school (including technical secondary school) n = 123 (9.8%), University including junior college) n = 840 (66.8%), and Postgraduate and above n = 274 (21.8%)	Worker: 573 (45.5%).
101	Juon 2016	Not reported	<High school 31 (13.4%), High school Plus 85 (36.8%), College graduate 67 (29.0%), and Grad school 48 (20.8%)	Employed: n = 157 (67.9%), Not employed: n = 74 (32.1%). Having health insurance: yes: n = 212 (52.8%) and no: n = 108 (47.2%)
102	Juraskova 2011	N=159: Christian n = 104 (66%) and other n = 52(34%)	Father's education: <12 years 40(25%), TAFE/diploma 17(11%), and University or higher degree 102 (64%)	Not reported
103	Kasting 2019	Not reported	Not reported	Not reported
106	Kempe 2001	Not reported	Not reported	Not reported
105	Kempe 2005	Not reported	Parent/guardian education (N = 5193): High school graduate or less n = 950 (18.3%), Some college n = 1182 (22.8%), and College graduate or more n = 3060 (58.9%)	Annual household income (N = 5193): <$50 000: n = 1795 (34.6%), $50 000 to <$100 000: n = 1935 (37.3%), and >=$100 000: n = 1482 (28.5%). Insurance: Private 80.3% in the intervention group and 82.1% in the control group; public: 16.0% in the intervention group and 14.8% in the control group; uninsured: 3.4% in the intervention group and 3.0% in the control group.
104	Kempe 2012	Not reported	All students were sixth grade.	All schools serve predominately low-income, minority populations
107	Kempe 2016	Not reported	Not reported	The study population was insured and lived in census tracts

	Study	Religion	Education level	Socio economic data
				with a median family income of $74900 (SD $32420), as assessed by using geocoding to estimate area-based socioeconomic measures.
108	Kempe 2020	Not reported	Not reported	Not reported
109	Kerpelman 2000	Not reported	Not reported	The study was conducted among low-income preschool children by imposing a sanction on families who failed to provide proof of up-to-date immunization status.
110	Khan 2023	Not reported	Not reported	Not reported
111	Kim 2020	Religion (N = 103): Buddhist n = 4 (3.9%), Catholic n = 31 (30.1%), Protestant n = 47 (45.6%), and none n = 21 (20.4%)	All are College Students	Not reported
112	Krieger 2000	Not reported	N = 1246 (Intervention group = 622, and control = 624); less than high school 16.3% in the intervention and 17.1% in the control, high school grad 28.2% in the intervention and 30.0% in the control, some college 24.3% in the intervention and 22.0% in the control, college grad 31.1% in the intervention and 30.9% in the control.	Household income: ≤$10,000 are 15.1% in the intervention and 18.3% in the control.
113	Kulle, 2024	Not reported	Not reported	Not reported
114	Lau 2012	Not reported	University student n = 316 (80.2%).	Not reported
115	LeBaron 2004	Not reported	Not reported	Not reported
116	Lee 2020	Not reported	Not reported	There are income data by intervention and control group but difficult to extract the overall percentage: $10K-29K≃ 26% in the intervention and 24% in the control; $30K-49K≃ 21.5% in the intervention and 20.5% in the control; $50K-69K ≃23% in the intervention and 22% in the control, and $>70K ≃23.% in the intervention and 23% in the control.
117	Lerner 2021a	Not reported	Not reported	Insurance, n (%): Private 20208 (91.7%), Public 1517 (6.9%), and Other or unknown 321 (1.5%).
117	Lerner 2021b	See Lerner 2021a	See Lerner 2021a	See Lerner 2021a

	Study	Religion	Education level	Socio economic data
117	Lerner 2021c	See Lerner 2021a	See Lerner 2021a	See Lerner 2021a
118	Leung 2017	Not reported	Baseline--->Below primary/elementary (Grade 1-6) = 29.5% (156/529), Primary/elementary or above = 70.5% (373/529): NB in the regression table---> Tertiary education 8.6% (32/374), Secondary education (middle school/high school/Grades 7–12) 32.1% (120/374), Primary 31.0% (116/374), and below primary 28.3% (106/374)	Not reported
119	Liao 2020	Not reported	Secondary or below = 124/291 (42.6%) and Tertiary or above = 167/291 (57.4%)	Income US$: <20,000=30/291 (10.3%), 20,000-40,000=119/291 (40.9%), and ≥40,000=152/291 (52.2%)
120	Lieu 2022	Not reported	Not reported	Neighbourhood Deprivation Index, percentile (N = 8287): <25th (least deprived): n = 1396 (16.8%), 25th-74th: n = 3561 (43.0%), 75th-89th: n = 1566 (18.9%), ≥90th (most deprived): n = 1281 (15.5%), and Unknown: n = 483 (5.8%)
121	Lin 2020	Not reported	Education (N = 180): High school 37 (20.6%), Junior college 23 (12.8%), University 87 (48.3%), and Graduate school 33 (18.3%)	Occupation: Have work: n = 127 (70.6%), No work: n = 40 (22.2%), Stopped working: n = 10 (5.6%), and Other: n = 3 (1.7%)
122	Ma 2018	Not reported	Education (N = 1720): <High school n = 167 (9.7%), High school graduate n = 490(28.5%), and >Some college n =1063 (61.8%)	Annual household income (N = 1527): <$20,000: n = 471 (30.8%), $20,000-$40,000: n = 544 (35.6%), >$40,000: n = 512 (33.5%). Health insurance status (n = 1753): No = 830 (47.3%), Yes = 923 (52.7%). Employment (n = 1755): Employed = 1026 (58.5%), and Unemployed/retired/homemaker = 729 (41.5%)
123	Ma 2021	Not reported	Not reported	Not reported
124	Mantzari 2015	Not reported	Not reported	It has data on mean and (SD) social deprivation (IMD): First time invitees Intervention group = M = 46.3 (13.12), First time invitees control group = M = 45.3 (13.0), Previous nonattenders intervention group = M = 35.3 (21.9), and Previous nonattenders control group= M = 36.2 (22.2). **Note**:- Social deprivation. Area-level social deprivation was measured using participants'

	Study	Religion	Education level	Socio economic data
				postcodes to calculate English Index of Multiple Deprivation (IMD) scores, which range from 0.37 (least deprived) to 85.46 (most deprived) (Community & Neighbourhoods, 2007). The IMD is a measure of deprivation in England based on area of residence.
125	Mason 2000	Not reported	Not reported	Not reported
126	Masson 2013	Not reported	High school education or above 269/489 (55.0%).	Employed: n = 78 (15.9%). Yearly income < $10 000: n = 298 (60.9%)
127	McCaul 2002	Not reported	Not reported	Not reported
128	Meharry 2014	Not reported	N = 133: ≤HS/GED 55 (41.3%), Some college 32 (24.1%), College degree 25 (18.8%), and ≥Graduate degree 19 (14.3%)	Income: <$25,000 = 44 (33.1%), $25,001–$50,000 = 13 (9.8%), $50,001–$75,000 = 6 (4.5%), ≥$75,001 = 24 (18.0%), and Did not disclose = 46 (34.6%). Employment: Full-time = 40 (30.1%), Part-time = 28 (21.1%), Not working = 46 (34.6%), and "Stay-at-home-mom" = 16 (12.0%).
129	Mehta 2022	Not reported	Not reported	Household income: < $35,000 / Unknown: n = 4396 (27.4%), $35,000 – 59,999: n = 4050 (25.2%), $60,000 – 76,099: n = 3092 (19.3%), and ≥ $76,100: n = 4507 (28.1%); (median (IQR), $): 57 126 (34 579-76 103)----->American Community Survey (2015-2019) Median Household Income at zip code level in 2019 inflation-adjusted dollars. Data were missing for 25 participants.
130	Menzies 2020	Not reported	Not reported	Not reported
131	Moniz 2013	Not reported	N = 204: Less than high school 19 (9.3%), High school or high school equivalency certificate 145 (71.1%), 2-y to 4-y college 39 (19.1%), and Postgraduate degree 1 (0.5%)	Household income: Less than $10,000 = 124 (60.8%), $10,000–40,000 = 55 (27%), $40,000–100,000 = 11 (5.4%), Greater than $100,000 = 4 (2%), Do not know = 10(4.9%). Insurance: None = 31 (15.2%), Medicaid or Medicare = 149 (73%), Private = 22 (10.8%), and Do not know: 2 (1%)
132	Munoz-Miralles 2022	Not reported	Not reported	Not reported
133	NCT05012163 2024	Not reported	Not reported	Not reported
134	NCT05536674 (Daly, 2023)	Not reported	Not reported	Not reported
135	NCT05248399, 2022	Not reported	Not reported	Not reported
136	NCT05534061 2022	Not reported	Not reported	Not reported

	Study	Religion	Education level	Socio economic data
137	NCT05537441 2022	Not reported	Not reported	Not reported
138	Nehme 2019	Not reported	Not reported	Study subjects are members of an Affordable Care Act insurance plan.
139	Nowalk 2010	Not reported.	Not reported	All study subjects are employees of companies.
140	Nyamathi 2009	Not reported	N = 865: High school graduate 73.9%	Employed 9.4%
141	Nyamathi 2010	Not reported	N = 148: High School Grad 81.5%.	Employed 16.7%
142	O'Grady 2022	Not reported	N = 310: Mothers education status: Tertiary degree 43 (13.9%), Diploma/certificate/trade 124 (40%), High school 67 (21.6%), Did not finish high school 67 (21.6%), and Declined/missing/unknown 10 (3.2%).	Total annual household income: \$104,000+: n = 55 (17.7%), \$78,000–103,999: n = 52 (16.8%), \$52,000–77,999: n = 49 (15.8%), \$0–51,999: n = 81 (26.1%), and Declined/missing/unknown: n = 73 (23.5%)
143	O'Leary 2015	Not reported	Not reported	Not reported
144	O'Leary 2019	Not reported	High school or less (50.7%), Technical, vocational or 2-year degree (20.8%), 4-year college or more (28.5%)	Not reported
145	Omer 2022	Not reported	N = 2092: Doctoral or professional degree 140 (8.1%), Master's degree 326 (18.8%), Bachelor's degree 616 (35.5%), Associate's degree 166 (9.6%), Postsecondary non-degree award 68 (3.9%), Some college, no degree 219 (12.6%),High school diploma or equivalent 182 (10.5%), No formal education credential 12 (0.7%), Prefer not to answer 5 (0.3%), and Missing 358 (17.1%).	Insurance type: Private: n = 1,435 (70.8%), Medicaid/CHP+: n = 476 (23.5%), Medicare: n = 7 (0.4%), Uninsured: n = 36 (1.8%), Other: n = 41 (2.0%), Unknown: n = 33 (1.6%), and Missing: n = 64 (3.0%)
146	Osborne, 2023	Information on religion is not clearly reported – could not be extracted.	School year (n = 702), 1st = 224 (31.9%), 2nd = 142 (20.2) , 3rd=170 (24.2) , 4th = 120 (17.1%) , 5th+ = 33 (4.7%), missing = 13 (1.8%)	Not reported
147	Otsuka 2013a	Not reported	Not reported	Insurance (N = 674): Private: n = 317(47%), Medicare: n = 339 (50.3%), Medicaid: n = 10 (1.5%), and self-pay/other: n = 8 (1.2%).

	Study	Religion	Education level	Socio economic data
147	Otsuka 2013b	See Otsuka 2013a	See Otsuka 2013a	See Otsuka 2013a
148	Otsuka-Ono 2019	Not reported	Mother's highest education level completed (N = 171): Middle/high school 21 (12.3%), Vocational school 41 (24%), Junior college 40(23.4%), university 65 (38%), Graduate 4 (2.3%)	Annual income (thousand yen): < 2000 = 2 (1.2%), 2000–3999 = 19 (11.1%), 4000–5999 = 66 (38.6%), 6000–7999 = 41 (24%), 8000–9999 = 27 (15.8%), and ≥ 10,000 = 13 (7.6%). Mother job status: Unemployed: 77 (45%), Full-time job: 71 (41.5%), part-time job: 22 (12.9%). Father job status: Full-time job: 165 (96.5%), self-employed: 6 (3.5%).
149	Patel 2014	Not reported	N = 365: High school or less 185 (50.7%), Technical-vocational or 2-year degree 76 (20.8%), and 4-year college or more 104 (28.5%)	Health insurance status (N = 365): No health insurance: n = 207 (56.7%), Private insurance: n = 100 (27.4%), and Public insurance: n = 58 (15.9%)
150	Patel 2022	Not reported	Not reported	Not reported
151	Porter-Jones 2009	Not reported	Not reported	Not reported
152	Pot 2017	(N=8062): Protestant=1490 (18.48%), Not protestant =6559 (81.36%); N missing=13 (0.16%)	Low = 1128 (13.99%), Middle = 3471 (43.05), and High = 3446 (42.74%); Missing = 7 (0.09%). Educational level was classified into low (less than secondary or vocational education), intermediate (secondary through preuniversity education) or high (professional or university education)	Not reported
153	Qin 2023	Not reported	N = 100: Below college= 30 (30.0%) and College and above =70 (70.0%)	Annual income (US$) : 4539$: n = 20 (20.0%), 4539–12103$: n = 22 (22.0%), 12104–22693$: n = 32 (32.0%), 22694–45386$: n = 17 (17.0%), and 45386$: = 9 (9.0%).
154	Quinlivan 2003	Not reported	Not reported	Socioeconomic status score (% low or destitute score) = 117/136 (86.0%), Homeless (yes) = 20/136 (14.7%)
155	Rand 2015	Not reported	Not reported	Insurance (N = 3812): Medicaid: n = 2241 (58.8%), SCHIP n = 1571 (41.2%).
156	Rand 2017	Not reported.	Not reported	Insurance: Public: n = 602 (80.4%), Private: n = 103 (13.7%), and None: n = 44 (5.9%).
158	Reiter 2018	Not reported	Education level (N = 150): Some college or less 94 (62.7%) and College degree or more 56 (37.3%).	Household income: Less than $50,000: n = 114 (76%) and $50,000 or more: n = 36 (24%). Health insurance: None: n = 27 (18%), On parents' insurance: n =

	Study	Religion	Education level	Socio economic data
				67 (44.7%, Insures self: n = 56 (37.3%)
157	Reiter 2023	Not reported	(N = 1227): High school or less=380 (31%) and Some college or more= 847 (69%).	Employment status: Employed full time or part time: n = 349 (28%), and Other: n = 878 (72%). Health insurance: Private: n = 768 (63%), public: n = 206 (17%), and None/don't know: n = 253 (21%).
160	Richman 2019	Not reported	Not reported	Employed n = 139/250 (56%). Insured n = 176/252 (70%).
159	Richman 2016	Not reported	Class standing (N = 263): First year 75 (28.5%), Sophomore 53 (20.1%), Junior 44 (16.7%), Senior 58 (22.0%), and Graduate student 33 (12.5%)	Not reported
161	Roca 2012	Not reported	Not reported	Not reported
162	Rodriguez 2022	Not reported	Not reported	Health insurance provider: City-based health care service: n = 28 (5.6%), Health maintenance organization/Kaiser: n = 7 (1.4%), Medicaid: n = 173 (34.8%), Medicare: n = 97 (19.6%), None: n = 95 (19.2%), Affordable Care Act: n = 21 (4.2%), Private: n = 103 (20.8%), Other: n = 23 (4.6%), and Veterans Health Administration: n = 5 (1.0%). Housing status: Housed: n = 408 (82.3%), Marginal: n = 19 (3.8%), and Unhoused: n = 68 (13.7%).
163	Rodriguez 2024	Not reported	Not reported	Self-reported health insurance coverage (Participants could have more than one selection) n = 767: City-based health care service: n = 68 (8.9%), HMO/Kaiser: n = 3 (0.4%), Medicaid: n = 193 (25.2%), Medicare: n = 146 (19.0%), None: n = 126 (16.4%), Affordable Care Act: n = 7 (0.9%), Private: n = 289 (37.7%), Other: n = 50 (6.5%), and Veterans Health Administration: n = 16 (2.1%). Housing status: Housed: n = 695 (90.6%), Marginal: n = 22 (2.9%), Unhoused: n = 50 (6.5%).
164	Ronzani 2022	Not reported	Not reported	Not reported
165	Saaksvuori 2022	Not reported	Not reported	Not reported
166	Saccardo 2024	Not reported	Not reported	Not reported
167	Saitoh 2017	Not reported	N = 188: Middle/high school 47 (25%), Junior college 94 (50%) , and College/graduate school 47 (25%)	Household annual income (thousands of yen) (N = 150): 0–3000: n= 16 (10.7%), 3000–4999: n = 70 (46.7%), 5000–6999: n = 48 (32%), 7000–9999: n = 17

	Study	Religion	Education level	Socio economic data
				(11.3%), and >=10,000: n =8 (5.3%). Maternal employment status (n=188): Unemployed: n = 133 (70.7%) and Employed: n = 55 (29.3%).
168	Santa Maria 2021	Not reported	Parent: Did Not Finish High School 96 (18.71%), High School Graduate 121 (23.59%), Vocational/Technical 66 (12.87%), Some College 119 (23.20%), and College Graduate 111 (21.64%. Youth attended school yes 498 (99.40%) and their current grade are from 4th to 9th.	Parent Insurance Status: None: n = 81 (15.70%), Medicaid: n = 217 (42.05%), Private: n = 168 (32.56%), and Other: n = 50 (9.69%)
169	Scarinci 2020	Not reported	Mean Education (years) – Mother 8.7 (3.5) for the vaccination arm and 8.9 (2.5) for the control arm.	Mean Monthly Income ((USD): 1,775.34 (1068.4) for the intervention arm and 1,549.64 (553.2) for the control arm. Employment Status-Mother (n=278): Full-time: n = 51 (18.3%), part-time: n = 87 (31.3%), homemaker: n = 130(46.8%), and unemployed: n = 10 (3.6%).
170	Scott, 2019	Not reported	Parent education high school or less 202/400 (50.5%)	Child Public insurance = 386/400 (96.5%)
171	Shegog 2022	Not reported	Parent College degree or more (n = 373): No 143 (38.1%), and Yes 232 (61.9%)	Parent Employed (N = 2375): No: n = 119 (31.7%), Yes: n = 256 (68.3%). Parent Insurance: Private health insurance (individual or employer provided): n = 287 (76.5%), Medicaid/Medicare/CHIP/S-CHIP: n = 77 (20.5%), Military Health Care (Tricare/VA/CHAMP-VA): n = 2 (0.5%), No insurance: n = 7 (1.9%), and Other: n = 2 (0.5%)
172	Shen, 2024	Not reported	Education (middle school or higher): 193/720 (26.8%)	Income (>CNY 2000): 506/720 (70.3%)
173	Shourie 2013	Not reported	N = 220: Up to 18 years 85 (38.6%), Beyond 18 years 134 (60.9%), and missing 1 (0.5%)	Employment: Full time: n = 107 (48.6%), part-time: n = 75 (34.1%), other: n = 36 (16.4%), and missing: n = 2 (0.9%)
174	Si 2022	Not reported	Parental education (n = 3739): Junior high school or below 1682 (44.99%), Senior high school (including vocational high school) 1114 (29.79%), and College (including technical college) and above 943 (25.22%)	Not reported

	Study	Religion	Education level	Socio economic data
175	Sitler 2018	Not reported	Caregiver educations (n=129): HS or less= 71.7%, some college= 21.1%, and college degree 7.2%.	Study population: rural low-income population of children enrolled in a WIC (Women/Infant/ Children) program. Eligibility for enrolment into this program is based upon income with the maximum income allowance of 185% above poverty.
177	Stockwell 2012	Not reported	Not reported	Insurance (N = 9213): None: n = 622 (6.8%), Medicaid/SCHIP: n = 8112 (88.0%), and private: n = 479 (5.2%). Study subjects are low-income, urban parents
178	Stockwell 2012	Not reported	Not reported	Insurance status for Text4Health–Adolescents (n = 361): Uninsured 41 (11.4%), Medicaid/SCHIP 290 (80.3%), and private 30 (8.3%). The study populations are an urban, low-income population
178	Stockwell 2012	Not reported	Not reported	Insurance status for Text4Health–Peds (n = 174): Uninsured 29 (16.7%), Medicaid/SCHIP 132 (75.9%), private 13 (7.5%). The study populations are an urban, low-income population
180	Stockwell 2014	Not reported	Not reported	N = 1153: Insurance at start of pregnancy: Uninsured: n = 360 (31.2%), Medicaid/SCHIP: n = 781 (67.7%), and Private: n = 12 (1.1%). The study population are low-income obstetric population.
176	Stockwell 2015	Not reported	<High school 110 (16.7%), High school 230 (34.8%), and At least some college 320 (48.5%).	Insurance: Medicaid/State Children's Health Insurance Program: n = 638 (96.7%), Commercial: n = 14 (2.1%), and Uninsured: n = 8 (1.2%). The study populations are low-income, urban, minority population.
179	Stockwell 2022	Not reported	Caregiver education: High school or less 22.3% (320), Vocational school or some college 17.9% (257), Associates or bachelors 36.3% (522), and Masters or doctorate 23.5% (338)	Child insurance type: Commercial insurance 54.5% (n = 1137) and Public insurance or uninsured 45.5% (n = 948)
181	Stolpe 2019	Not reported	Education in ZIP code, % undergrad or higher (SD) (n=21971; 10962 control and 11009 intervention): 26.8% (12.5) for control and 26.6% (12.3) for the intervention	Median income in ZIP code, mean (SD), $: 67,079 (17,034) for the control and 67,019 (17,135) for the intervention
182	Strathdee 2023	Not reported	(n=135): Median # of years of education completed (IQR)=12 (10,13).	Monthly income <500 USD: n = 64 (47.4%). Income worse since Covid-19 pandemic began: n = 93 (68.9%). Homeless: n = 99

	Study	Religion	Education level	Socio economic data
				(73.3%). Lacks health insurance: n = 18 (13.3%).
183	Stuck 2015	N = 2284: Protestant 1196 (52.4%), Catholic 1115 (48.8%), No religious affiliations 48 (2.1%), and other/unknown 105 (4.6%)	N = 2284: Compulsory education or less (<=9 y) 994 (43.5%), Secondary-level education (10–12 y) 1042 (45.6%), Tertiary-level education (>12 y) 194 (8.5%), and unknown 54 (2.4%).	Socio-economic status- Swiss neighbourhood index: 61.2 ± 7.3 for the intervention group and 60.8 ± 7.4 for the control group. Higher scores denote higher levels of socio-economic status.
184	Suh 2012	Not reported	Not reported	Insurance status (n = 799): Private 89.6% (717), public 8.1% (65), and missing 2.3% (18)
185	Suzuki, 2022	Not reported	N = 2175: Less than high school graduate 21 (1.4%), High school graduate 355 (22.9%), and More than high school graduate 1174 (75.7%)	Household income (million JPY/year), Mean (SD)= 7.41 (4.68); JPY 110=US $1 USD
186	Sweeney 2014	Not reported	Participants were undergraduate students.	Not reported
187	Szilagyi 2006	Not Reported.	Not reported	Insurance (n = 3006): Medicaid, fee for service: n = 709 (26.3%), Medicaid, managed care (includes SCHIP): n = 289 (9.6%), Private, fee for service: n = 166 (5.5%), Private, managed care: n = 1302 (43.3%), Uninsured: n = 84 (2.8%), and other or unknown: n = 307 (10.2%).
188	Szilagyi 2011	Not reported.	Not reported	Insurance type (n = 7549): Medicaid managed care: n = 4935 (65.4%), Medicaid fee for service: n = 626 (8.3%), Uninsured: n = 445 (5.9%), SCHIP/State Children’s Health Insurance Program: n = 989 (13.1%), and Commercial: n = 550 (7.3%). Study area population: Almost 80% of adolescents in the city live below the poverty level.
189	Szilagyi 2013	Not reported	Not reported	Insurance (n = 4115): Medicaid managed care: n = 1916 (46.6%) and SCHIP: n = 2199 (53.4%). Study subjects are low-income adolescents.
190	Szilagyi 2018	Not reported	The study subjects are elementary school children	Not reported
191	Szilagyi 2019	Not reported	The study subjects are elementary school children	Study subject are among urban (mostly low-income income: ~90% free and reduced lunch) elementary school children.
192	Szilagyi 2020	Not reported	Not reported	Not reported

	Study	Religion	Education level	Socio economic data
193	Szilagyi 2020	Not reported	Not reported	Insurance (n = 164,205): Private 85.2%, Public 13.5%, and Other or unknown 1.3%.
194	Szilagyi 2020	Not reported	Not reported	Not reported
195	Szilagyi 2024a	Not reported	Not reported	Primary insurer: Private 218728 (83.4%), public 39008 (14.9%), other or unknown 4349 (1.7%).
195	Szilagyi, 2024b	See Szilagyi 2024a	See Szilagyi 2024a	See Szilagyi 2024a
195	Szilagyi, 2024c	See Szilagyi 2024a	See Szilagyi 2024a	See Szilagyi 2024a
196	Tentori 2022	Not reported	Not reported	Not reported
197	Terrell-Perica 2001	Not reported	Not reported	Not reported
198	Thilly, 2024	Not reported	Not reported	Not reported
199	Tiro 2015	Not reported	Not reported	Insurance: (n = 814): Public 603 (74.1%), private 16 (2.0%), and No insurance 195 (24.0%).
200	Topp 2013	Not reported	Completed four or more years of high school (n = 139); 60%	Government benefits main source of income = 86%, Unstable accommodation last six months = 37%
201	Tubiana 2021	Not reported	Not reported.	Not reported
202	Tull 2019	Not reported	All study subjects are year 7 students.	Not reported
203	Ueberroth 2022	Not reported	Not reported	Not reported
204	Usami 2009	Not reported	Not reported	Not reported
205	Vanderpool 2013	Not reported	N = 345: 48.0% reported some college as their highest level of education.	Only one-quarter (25.6%) were employed full-time
206	Viver 2000	Not reported	Pre-school children	Not reported
207	Wang 2021	Not reported	N = 624: Secondary or below 95 (15.2%) and University or above 529 (84.8%)	Current employment status: Full time: n = 492 (78.8%), Part-time/unemployed/retired/students: n = 132 (21.2%). Personal monthly income (HK $; US $): <HK $10,000 (US $1290): n = 101(16.2%), HK $10,000-$19,999 (US$1290-$2580): n = 207 (33.2%), HK $20,000-$39,999 (US$2580-$5161): n = 223 (35.7%), ≥HK $40,000 (US $5161): n =90 (14.4%), Refuse to disclose: n = 3 (0.5%).
208	Wang 2023	Not reported	(N = 396): ≤Primary 164 (41.4%), Secondary 189 (47.7%), and ≥Tertiary 43 (10.9%)	Monthly household income, HK$ (US $): <20 000 (2580) 294 (74.2%), ≥20 000 (2580) 52 (13.2%), and Refuse to disclose 50 (12.6%).
209	Weaver 2014	Not reported	Not reported	Employment (n=210): Unemployed: n = 183 (87%), Employed or student: n = 25 (12%), and other: n = 2 (1%). Accommodation: Owner occupied: n = 11 (5%), Rented

	Study	Religion	Education level	Socio economic data
				private: n = 47 (22%), Rented (LA, HA): n = 72 (34%), Living with relatives: n = 17 (8%), Bed and breakfast or hotel: n = 5 (2%), Hostel: n = 21 (10%), NFA: n = 26 (12%), and other: n = 11 (5%).
210	Wijesundara 2020a	Not reported	Not reported	Not reported
210	Wijesundara 2020b	See Wijesundara 2020a	See Wijesundara 2020a	See Wijesundara 2020a
211	Winston 2007	Not reported	There is data for educational level but does not have enough information to extract	Not reported
212	Wiseman 2016	Not reported	parent education (n=136): Elementary 9 (7%), High school 102 (75%), Some college 16 (12%), College/university 5 (3.7%).	Family Income: Less than $10,000: n = 63 (51%), $10,001-25,000: n = 43 (35%), $25,001-$40,000: n = 9 (7%), $40,000 and above: n = 8 (7%).
213	Wong 2016	Not reported	Maternal education (n = 321): Compulsory secondary or below 23 (7.2%), Upper secondary 53 (41.1%), Some post-secondary 30 (9.4%),and University degree or above 136 (42.4%).	Family income: Below median: n = 93 (29.0%) and Above median: N = 228 (71.0%) Median household income in HK in 2011 was $20,000 to $24,999 HKD per month (1USD=7.7HKD).
214	Wouters, 2007	Not reported	Not reported	All are commercial sex workers (CSW). Working sector - Street 21% (N = 127), Bar 18% (n = 112), Window 31% (n = 190), Private 26% (n = 157), Missing 5% (n =29).
215	Wright 2012	Not reported	Not reported	Insurance (n = 3,979): private: n = 3369 (84.7%), Medicare: n = 562 (14.15%), Medicaid/free care: n = 14 (0.4%), self-pay/none: n = 31 (0.8%), and unknown: n = 3(0.1%). Lived in higher income neighbourhoods (median: $54,617 versus $52,012) and the Mean median neighbourhood income (SD): was 55,385 (9,748) for the active control arm and 54,024 (10,406) for the intervention arm.
216	Wynn 2021	Not reported	Parental education (n = 956): <High school 219 (22.9%), Finished high school 354 (37.1%), and >High school 382 (40%).	Insurance (n = 956): Public: n = 903 (94.5%).
217	Xu 2022	Not reported	N = 196: Primary and below 27(13.8%), Middle or high school 104 (53.1%), college 41	Occupation: Teacher: n = 4 (2.0%), Service worker: n = 17(8.7%), Medical worker: n = 8 (4.1%), Worker or Farmer: n = 40

	Study	Religion	Education level	Socio economic data
			(20.9%), and Bachelor degree or above 24 (12.2%).	(20.4%), Government employee: n = 12 (6.1%), Housework and unemployment: n = 74 (37.8%), and other: n = 41 (20.9%).
218	Yeung 2018	Not reported	Maternal highest educational level (n = 833): Junior secondary or below 72 (8.6%), Senior secondary 308 (37%), Post-secondary or matriculation 188 (22.6%), University degree or above 265 (31.8%) Low: n = 1128 (13.99%); Middle: n = 3471 (43.05%); High: n = 3445 (42.74%).	Monthly household income in $ (n = 820): ≤20,000: n = 175 (21.3%), 20,000–29,999: n = 188 (22.9%), 30,000–39,999: n = 191 (23.3%), ≥40,000: n = 266 (32.4%)
219	Yokum 2018	Not reported	Not reported	Not reported
220	Yudin 2016	Not reported	Education (n = 277): post-secondary 87.7%.	Household income > $100,000.00 (N = 255): 112 (43.9%).
221	Zhang 2018a	Not reported	Not reported	60.53% spent at least a week in the prior month living on the street. In shelter ≥ 1 wk.: n = 13.53.
222	Zhang 2018b	Not reported	Highest education (n = 311): No school education 70 (22.5%), Primary school 123 (39.5%), Junior high school 73 (23.5%), Senior high school 29 (9.3%), college graduation 9 (2.9%), University graduation and above 5 (1.6%), and other 2 (0.6%).	Not reported
223	Zhang 2022	Not reported	Education of parents: Junior high school or below 345 (35.7%), Senior high school 285 (29.5%), and college and above 337 (34.9%).	Living expenses per month (CNY*): <1,000: n = 201 (20.8%), 1,000~2,000: n = 674 (69.75%), and >2,000: n = 92 (9.5%).
224	Zuniga de N uncio 2003	Not reported	Not reported	Employment (n = 348): Employed = 65 (18.7%), Not employed = 279 (80.2%), and unknown/missing = 4 (1.1%). Overall, prenatal care expenses were covered by Medical for 69 % of women, 28% paid cash, and only 3% had private insurance.

I: Risk of bias summary plots

Traffic light plots showing risk of bias assessments for each study. Figures produced using Robvis tool: McGuinness, LA, Higgins, JPT. Risk-of-bias Visualization (robvis): An R package and Shiny web app for visualizing risk-of-bias assessments. Res Syn Meth. 2020; 1- 7. https://doi.org/10.1002/jrsm.1411

Traffic-light plot showing risk of bias for individually randomised RCTs.

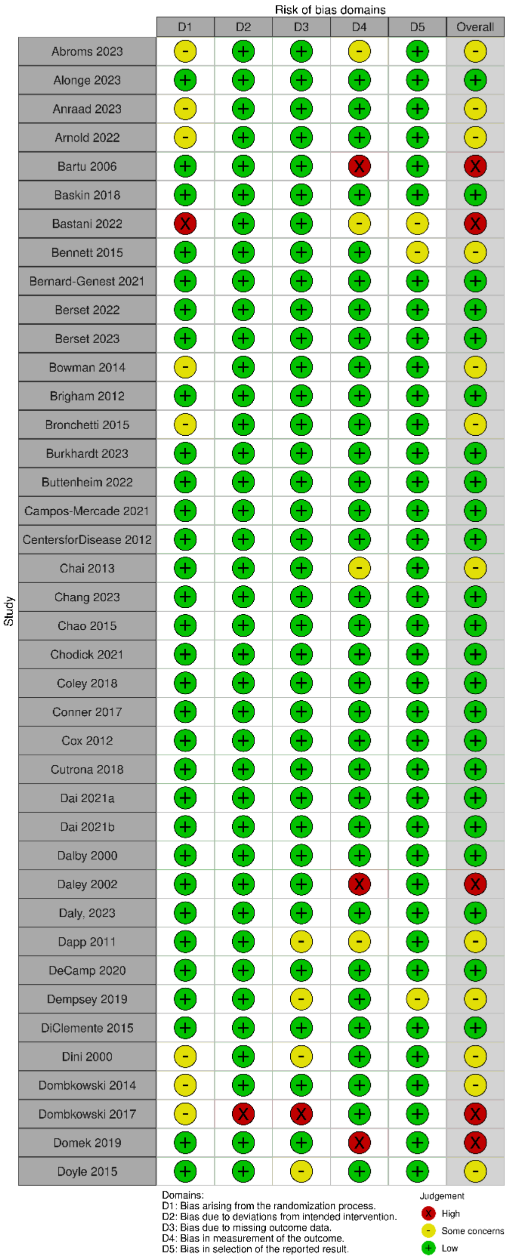

Risk of bias domains

Study	D1	D2	D3	D4	D5	Overall
El-Mohandes 2003	+	+	X	+	+	X
Fernandez 2022	+	+	-	X	+	X
Fiks 2013	+	+	+	+	+	+
Fitzpatrick 2018	+	+	-	+	+	-
Frew 2016	-	+	+	-	+	-
Gerend 2021	-	+	+	+	+	-
Glanz 2017	-	+	-	+	+	-
Glanz 2020	+	+	-	+	+	-
Goodman 2015	+	+	+	+	+	+
Gurfinkel 2021a	-	+	+	+	+	-
Gurfinkel 2021b	-	+	+	+	+	-
Haff 2023	-	+	+	+	+	-
Hambridge 2009	+	+	+	+	+	+
Hanley 2023	+	+	+	+	+	+
Hannan 2013	+	+	+	-	+	-
Henrikson 2018	-	+	+	+	+	-
Higginbotham 2012	X	+	+	+	+	X
Hofstetter 2015a	+	+	+	+	+	+
Hofstetter 2015b	+	+	+	+	+	+
Hopfer 2012	-	+	+	-	+	-
Hu 2017	-	+	-	+	+	-
Hu 2018	-	+	+	+	+	+
Hu 2021	+	+	+	+	+	+
Huf 2024	+	+	+	+	+	+
Hurley 2018a	+	+	+	+	+	+
Hurley 2018b	+	+	+	+	+	+
Hurley 2019	+	+	+	+	+	+
Hwang 2010a	X	+	-	+	+	X
Hwang 2010b	X	+	-	+	+	X
Irigoyen 2006	+	+	+	+	+	+
Janitz 2023	-	+	X	-	+	X
Jiang 2022	+	+	-	-	+	-
Johansen 2023	+	+	+	+	+	+
Johnson 2003	-	+	+	X	+	X
Jordan 2015	+	+	X	X	+	X
Joseph 2016	X	+	+	+	+	X
Ju 2024	X	+	-	+	+	X
Juon 2016	-	+	-	+	+	-
Juraskova 2011	-	+	-	-	+	-

Domains:
D1: Bias arising from the randomization process.
D2: Bias due to deviations from intended intervention.
D3: Bias due to missing outcome data.
D4: Bias in measurement of the outcome.
D5: Bias in selection of the reported result.

Judgement
X High
- Some concerns
+ Low

Risk of bias domains

Study	D1	D2	D3	D4	D5	Overall
Kasting 2019	+	+	+	+	+	+
Kempe 2001	+	+	X	X	+	X
Kempe 2005	+	+	+	+	+	+
Kempe 2012	-	+	+	+	+	-
Kempe 2020	+	+	+	+	+	+
Kerpelman 2000	-	+	-	+	+	-
Khan 2023	+	+	+	+	+	+
Kim 2020	+	+	-	-	+	-
Krieger 2000	-	+	-	-	+	X
Lau 2012	+	+	-	+	+	-
LeBaron 2004	+	+	+	-	+	-
Lee 2020	+	+	+	+	+	+
Lerner 2021a	-	+	+	+	+	-
Lerner 2021b	-	+	+	+	+	-
Lerner 2021c	-	+	+	+	+	-
Leung 2017	+	+	+	+	+	+
Liao 2020	+	+	-	-	+	-
Lieu 2022	+	+	+	+	+	+
Ma 2020	+	+	X	+	+	X
Ma 2022	-	+	+	-	+	-
Mantzari 2015	+	+	+	+	+	+
Mason 2000	-	+	+	+	+	+
Masson 2013	+	+	+	+	+	+
Meharry 2014	-	+	+	+	+	-
Mehta 2022	-	+	+	+	+	-
Menzies 2020	+	+	+	+	+	+
Moniz 2013	+	+	+	+	+	+
NCT05012163 2024	-	+	+	+	+	+
NCT05248399 2022	-	+	-	-	+	+
NCT05534061	+	+	+	+	+	+
NCT05537441	-	+	+	+	+	-
Nehme 2019	+	+	+	+	+	+
Nyamanthi 2010	-	+	+	+	+	-
O'Grady 2022	+	+	+	+	+	+
O'Leary 2015	+	+	+	+	+	+
O'leary 2019	-	+	+	+	+	-
Omer 2022	+	+	+	+	+	+

Domains:
D1: Bias arising from the randomization process.
D2: Bias due to deviations from intended intervention.
D3: Bias due to missing outcome data.
D4: Bias in measurement of the outcome.
D5: Bias in selection of the reported result.

Judgement
X High
- Some concerns
+ Low

Risk of bias domains

Study	D1	D2	D3	D4	D5	Overall
Kasting 2019	+	+	+	+	+	+
Kempe 2001	+	+	X	X	+	X
Kempe 2005	+	+	+	+	+	+
Kempe 2012	-	+	+	+	+	-
Kempe 2020	+	+	+	+	+	+
Kerpelman 2000	-	+	-	+	+	-
Khan 2023	+	+	+	+	+	+
Kim 2020	+	+	-	-	+	-
Krieger 2000	-	+	-	-	+	X
Lau 2012	+	+	-	+	+	-
LeBaron 2004	+	+	+	-	+	-
Lee 2020	+	+	+	+	+	+
Lerner 2021a	-	+	+	+	+	-
Lerner 2021b	-	+	+	+	+	-
Lerner 2021c	-	+	+	+	+	-
Leung 2017	+	+	+	+	+	+
Liao 2020	+	+	-	-	+	-
Lieu 2022	+	+	+	+	+	+
Ma 2020	+	+	X	+	+	X
Ma 2022	-	+	+	-	+	-
Mantzari 2015	+	+	+	+	+	+
Mason 2000	-	+	+	+	+	+
Masson 2013	+	+	+	+	+	+
Meharry 2014	-	+	+	+	+	-
Mehta 2022	-	+	+	+	+	-
Menzies 2020	+	+	+	+	+	+
Moniz 2013	+	+	+	+	+	+
NCT05012163 2024	-	+	+	+	+	+
NCT05248399 2022	-	+	-	-	+	+
NCT05534061	+	+	+	+	+	+
NCT05537441	-	+	+	+	+	-
Nehme 2019	+	+	+	+	+	+
Nyamanthi 2010	-	+	+	+	+	-
O'Grady 2022	+	+	+	+	+	+
O'Leary 2015	+	+	+	+	+	+
O'leary 2019	-	+	+	+	+	-
Omer 2022	+	+	+	+	+	+

Domains:
D1: Bias arising from the randomization process.
D2: Bias due to deviations from intended intervention.
D3: Bias due to missing outcome data.
D4: Bias in measurement of the outcome.
D5: Bias in selection of the reported result.

Judgement
X High
- Some concerns
+ Low

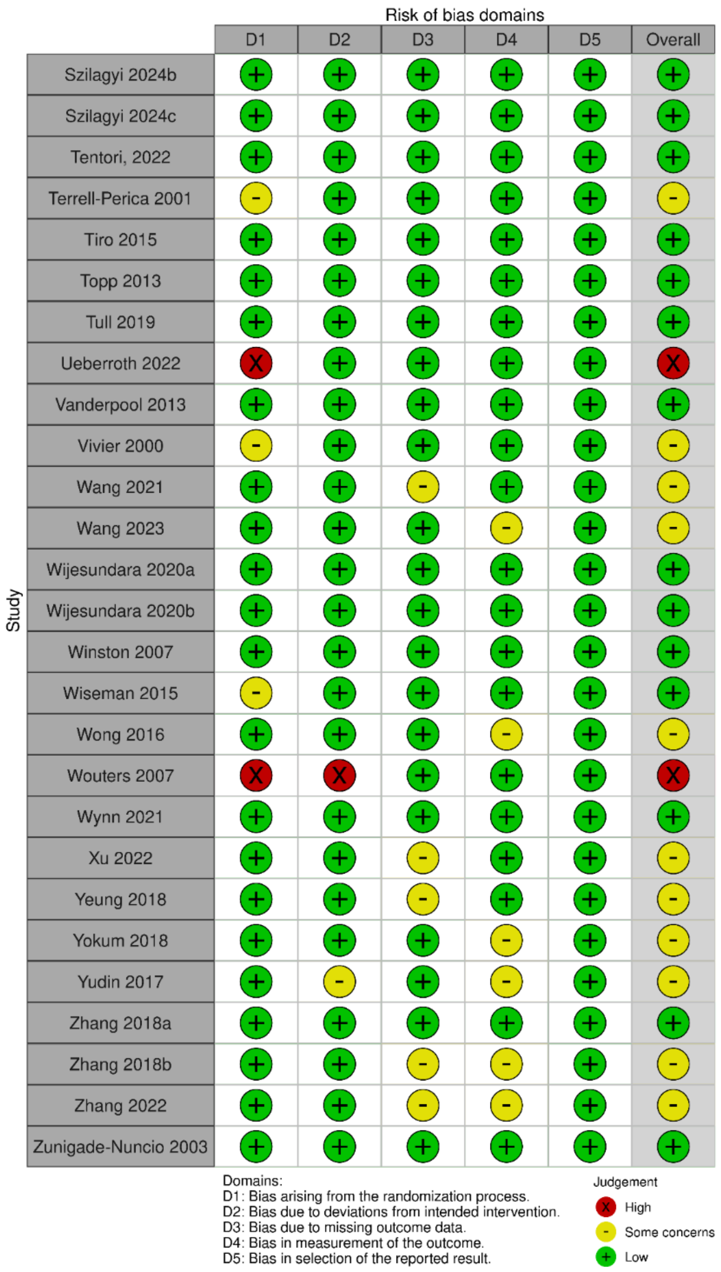
Risk of bias domains
D1 D2 D3 D4 D5 Overall
Study
Szilagyi 2024b + + + + + +
Szilagyi 2024c + + + + + +
Tentori, 2022 + + + + + +
Terrell-Perica 2001 - + + + + -
Tiro 2015 + + + + + +
Topp 2013 + + + + + +
Tull 2019 + + + + + +
Ueberroth 2022 X + + + + X
Vanderpool 2013 + + + + + +
Vivier 2000 - + + + + -
Wang 2021 + + - + + -
Wang 2023 + + + - + -
Wijesundara 2020a + + + + + +
Wijesundara 2020b + + + + + +
Winston 2007 + + + + + +
Wiseman 2015 - + + + + +
Wong 2016 + + + - + -
Wouters 2007 X X + + + X
Wynn 2021 + + + + + +
Xu 2022 + + - + + -
Yeung 2018 + + - + + -
Yokum 2018 + + + - + -
Yudin 2017 + - + - + -
Zhang 2018a + + + + + +
Zhang 2018b + + - - + -
Zhang 2022 + + - - + -
Zunigade-Nuncio 2003 + + + + + +
Domains:
D1: Bias arising from the randomization process.
D2: Bias due to deviations from intended intervention.
D3: Bias due to missing outcome data.
D4: Bias in measurement of the outcome.
D5: Bias in selection of the reported result.
Judgement
X High
- Some concerns
+ Low

Risk of bias domains

Study	D1	D2	D3	D4	D5	Overall
El-Mohandes 2003	+	+	X	+	+	X
Fernandez 2022	+	+	-	X	+	X
Fiks 2013	+	+	+	+	+	+
Fitzpatrick 2018	+	+	-	+	+	-
Frew 2016	-	+	+	-	+	-
Gerend 2021	-	+	+	+	+	-
Glanz 2017	-	+	-	+	+	-
Glanz 2020	+	+	-	+	+	-
Goodman 2015	+	+	+	+	+	+
Gurfinkel 2021a	-	+	+	+	+	-
Gurfinkel 2021b	-	+	+	+	+	-
Haff 2023	-	+	+	+	+	-
Hambridge 2009	+	+	+	+	+	+
Hanley 2023	+	+	+	+	+	+
Hannan 2013	+	+	+	-	+	-
Henrikson 2018	-	+	+	+	+	-
Higginbotham 2012	X	+	+	+	+	X
Hofstetter 2015a	+	+	+	+	+	+
Hofstetter 2015b	+	+	+	+	+	+
Hopfer 2012	-	+	+	-	+	-
Hu 2017	-	+	-	+	+	-
Hu 2018	-	+	+	+	+	+
Hu 2021	+	+	+	+	+	+
Huf 2024	+	+	+	+	+	+
Hurley 2018a	+	+	+	+	+	+
Hurley 2018b	+	+	+	+	+	+
Hurley 2019	+	+	+	+	+	+
Hwang 2010a	X	+	-	+	+	X
Hwang 2010b	X	+	-	+	+	X
Irigoyen 2006	+	+	+	+	+	+
Janitz 2023	-	+	X	-	+	X
Jiang 2022	+	+	-	-	+	-
Johansen 2023	+	+	+	+	+	+
Johnson 2003	-	+	+	X	+	X
Jordan 2015	+	+	X	X	+	X
Joseph 2016	X	+	+	+	+	X
Ju 2024	X	+	-	+	+	X
Juon 2016	-	+	-	+	+	-
Juraskova 2011	-	+	-	-	+	-

Domains:
D1: Bias arising from the randomization process.
D2: Bias due to deviations from intended intervention.
D3: Bias due to missing outcome data.
D4: Bias in measurement of the outcome.
D5: Bias in selection of the reported result.

Judgement
X High
- Some concerns
+ Low

Risk of bias domains

Study	D1	D2	D3	D4	D5	Overall
Kasting 2019	+	+	+	+	+	+
Kempe 2001	+	+	X	X	+	X
Kempe 2005	+	+	+	+	+	+
Kempe 2012	-	+	+	+	+	-
Kempe 2020	+	+	+	+	+	+
Kerpelman 2000	-	+	-	+	+	-
Khan 2023	+	+	+	+	+	+
Kim 2020	+	+	-	-	+	-
Krieger 2000	-	+	-	-	+	X
Lau 2012	+	+	-	+	+	-
LeBaron 2004	+	+	+	-	+	-
Lee 2020	+	+	+	+	+	+
Lerner 2021a	-	+	+	+	+	-
Lerner 2021b	-	+	+	+	+	-
Lerner 2021c	-	+	+	+	+	-
Leung 2017	+	+	+	+	+	+
Liao 2020	+	+	-	-	+	-
Lieu 2022	+	+	+	+	+	+
Ma 2020	+	+	X	+	+	X
Ma 2022	-	+	+	-	+	-
Mantzari 2015	+	+	+	+	+	+
Mason 2000	-	+	+	+	+	+
Masson 2013	+	+	+	+	+	+
Meharry 2014	-	+	+	+	+	-
Mehta 2022	-	+	+	+	+	-
Menzies 2020	+	+	+	+	+	+
Moniz 2013	+	+	+	+	+	+
NCT05012163 2024	-	+	+	+	+	+
NCT05248399 2022	-	+	-	-	+	+
NCT05534061	+	+	+	+	+	+
NCT05537441	-	+	+	+	+	-
Nehme 2019	+	+	+	+	+	+
Nyamanthi 2010	-	+	+	+	+	-
O'Grady 2022	+	+	+	+	+	+
O'Leary 2015	+	+	+	+	+	+
O'leary 2019	-	+	+	+	+	-
Omer 2022	+	+	+	+	+	+

Domains:
D1: Bias arising from the randomization process.
D2: Bias due to deviations from intended intervention.
D3: Bias due to missing outcome data.
D4: Bias in measurement of the outcome.
D5: Bias in selection of the reported result.

Judgement
X High
- Some concerns
+ Low

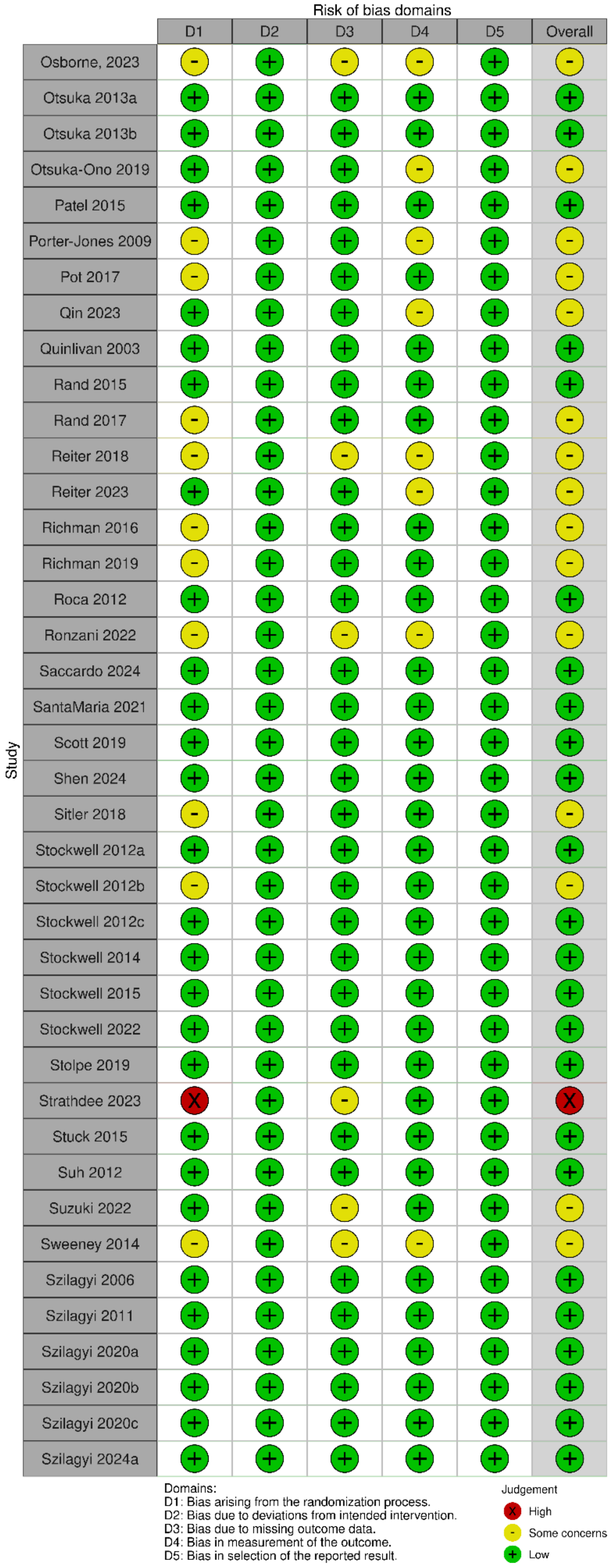
Risk of bias domains
Study
D1 D2 D3 D4 D5 Overall
Osborne, 2023: - + - - + -
Otsuka 2013a: + + + + + +
Otsuka 2013b: + + + + + +
Otsuka-Ono 2019: + + + - + -
Patel 2015: + + + + + +
Porter-Jones 2009: - + + - + -
Pot 2017: - + + + + -
Qin 2023: + + + - + -
Quinlivan 2003: + + + + + +
Rand 2015: + + + + + +
Rand 2017: - + + + + -
Reiter 2018: - + - - + -
Reiter 2023: + + + - + -
Richman 2016: - + + + + -
Richman 2019: - + + + + -
Roca 2012: + + + + + +
Ronzani 2022: - + - - + -
Saccardo 2024: + + + + + +
SantaMaria 2021: + + + + + +
Scott 2019: + + + + + +
Shen 2024: + + + + + +
Sitler 2018: - + + + + -
Stockwell 2012a: + + + + + +
Stockwell 2012b: - + + + + -
Stockwell 2012c: + + + + + +
Stockwell 2014: + + + + + +
Stockwell 2015: + + + + + +
Stockwell 2022: + + + + + +
Stolpe 2019: + + + + + +
Strathdee 2023: X + - + + X
Stuck 2015: + + + + + +
Suh 2012: + + + + + +
Suzuki 2022: + + - + + -
Sweeney 2014: - + - - + -
Szilagyi 2006: + + + + + +
Szilagyi 2011: + + + + + +
Szilagyi 2020a: + + + + + +
Szilagyi 2020b: + + + + + +
Szilagyi 2020c: + + + + + +
Szilagyi 2024a: + + + + + +
Domains:
D1: Bias arising from the randomization process.
D2: Bias due to deviations from intended intervention.
D3: Bias due to missing outcome data.
D4: Bias in measurement of the outcome.
D5: Bias in selection of the reported result.
Judgement
X High
- Some concerns
+ Low

Risk of bias domains

Study	D1	D2	D3	D4	D5	Overall
Szilagyi 2024b	+	+	+	+	+	+
Szilagyi 2024c	+	+	+	+	+	+
Tentori, 2022	+	+	+	+	+	+
Terrell-Perica 2001	-	+	+	+	+	-
Tiro 2015	+	+	+	+	+	+
Topp 2013	+	+	+	+	+	+
Tull 2019	+	+	+	+	+	+
Ueberroth 2022	X	+	+	+	+	X
Vanderpool 2013	+	+	+	+	+	+
Vivier 2000	-	+	+	+	+	-
Wang 2021	+	+	-	+	+	-
Wang 2023	+	+	+	-	+	-
Wijesundara 2020a	+	+	+	+	+	+
Wijesundara 2020b	+	+	+	+	+	+
Winston 2007	+	+	+	+	+	+
Wiseman 2015	-	+	+	+	+	+
Wong 2016	+	+	+	-	+	-
Wouters 2007	X	X	+	+	+	X
Wynn 2021	+	+	+	+	+	+
Xu 2022	+	+	-	+	+	-
Yeung 2018	+	+	-	+	+	-
Yokum 2018	+	+	+	-	+	-
Yudin 2017	+	-	+	-	+	-
Zhang 2018a	+	+	+	+	+	+
Zhang 2018b	+	+	-	-	+	-
Zhang 2022	+	+	-	-	+	-
Zunigade-Nuncio 2003	+	+	+	+	+	+

Domains:
D1: Bias arising from the randomization process.
D2: Bias due to deviations from intended intervention.
D3: Bias due to missing outcome data.
D4: Bias in measurement of the outcome.
D5: Bias in selection of the reported result.

Judgement
X High
- Some concerns
+ Low

Risk of bias summary plots for cluster RCTs.

Risk of bias domains

Study	D1	D1b	D2	D3	D4	D5	Overall
Arthur 2002	-	+	+	+	+	+	-
Berg 2004	+	+	+	+	X	+	X
Berg 2008	+	+	+	+	X	+	X
Berkhout 2018	-	+	+	+	X	+	X
Bethke 2024	+	+	+	+	+	+	+
Bian 2023	-	+	+	+	-	+	-
Borg 2018	+	+	+	+	+	+	+
Bourgeois 2008	+	-	+	-	+	+	-
Cataldi 2024	+	+	+	+	+	+	+
Clayton 2021a	-	+	+	-	+	+	-
Clayton 2021b	+	+	+	-	+	+	-
Daley 2014	X	+	+	-	+	X	X
Daniels 2007	-	X	+	+	-	+	X
Davies 2017	+	+	+	+	+	+	+
Dombkowski 2017	X	-	+	-	+	+	X
Esposito 2018	+	+	+	+	+	+	+
Ferreira 2022	-	-	+	+	+	+	-
Goodyear-Smith 2012	+	+	+	+	+	+	+
Grandahl 2016	+	X	+	+	+	-	X
Harari 2008	+	+	+	-	+	+	-
Hess 2013	-	+	+	+	+	+	-
Ho 2019	+	+	+	+	+	+	+
Howell-Jones 2023a	+	+	+	-	+	+	-
Howell-Jones 2023b	+	+	X	+	+	+	X
Hull 2002	+	+	+	-	+	+	-
Humiston 2014a	+	+	+	+	+	+	+
Humiston 2014b	+	+	+	+	+	+	+
Hurtaud 2023	-	-	+	+	+	+	-
Isrctn 2021	+	+	+	+	+	+	+
Jackson 2011	+	+	+	-	-	+	-
Kempe 2016	+	-	+	+	+	+	-
Kulle 2024	-	+	+	+	-	+	-
Ma 2018	-	-	+	-	X	+	X
McCaul 2002	-	+	+	+	-	-	-
Munoz-Miralles 2022	-	+	+	+	+	+	-
Nowalk 2018	-	+	+	+	+	+	-
Nyamathi 2009	-	-	+	+	+	+	-
Patel 2014	-	-	+	+	+	+	-
Rodriguez 2022	X	+	+	+	-	+	X
Rodriguez 2024	X	-	+	+	+	+	X
Saaksvouri 2022	+	+	+	+	+	+	+
Saitoh 2017	+	+	+	+	-	+	-
Scarinci 2020	-	+	+	+	+	+	-
Shegog 2022	+	+	+	-	-	+	-
Shourie 2013	+	+	+	+	+	+	+
Si 2022	-	+	+	-	-	+	-
Szilagyi 2013	+	+	+	+	+	+	+
Szilagyi 2018	+	+	+	+	+	+	+
Szilagyi 2019	-	+	+	+	+	+	-
Thilly 2024	+	+	+	+	+	+	+
Tubiana 2021	-	+	+	-	-	+	-
Usami 2009	-	+	+	+	-	+	-
Weaver 2014	+	+	+	+	+	+	+
Wright 2012	-	-	+	-	+	+	-

Domains:
D1 : Bias arising from the randomization process.
D1b: Bias arising from the timing of identification and recruitment of Individual participants in relation to timing of randomization.
D2 : Bias due to deviations from intended intervention.
D3 : Bias due to missing outcome data.
D4 : Bias in measurement of the outcome.
D5 : Bias in selection of the reported result.

Judgement
X High
- Some concerns
+ Low

J: Subgroup analyses

Age group: Adults

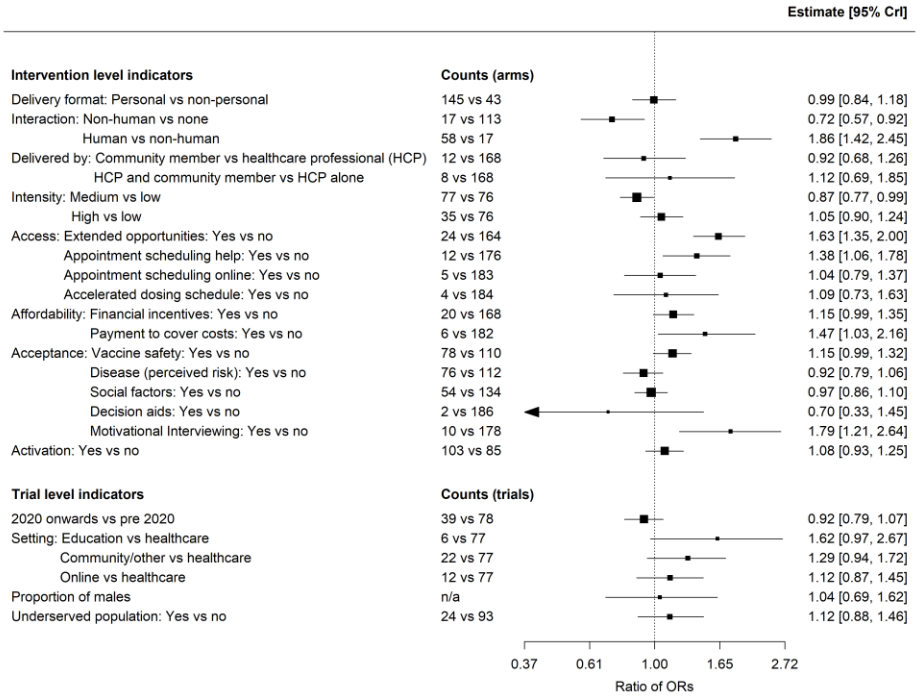

Age group: Adolescents/young adults

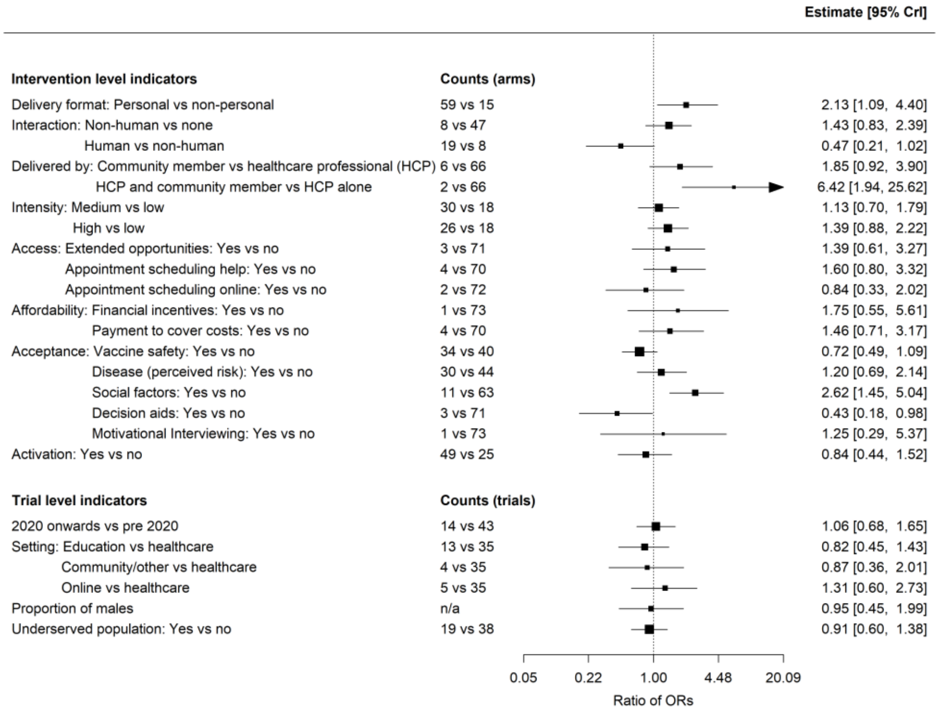

Age group: Children

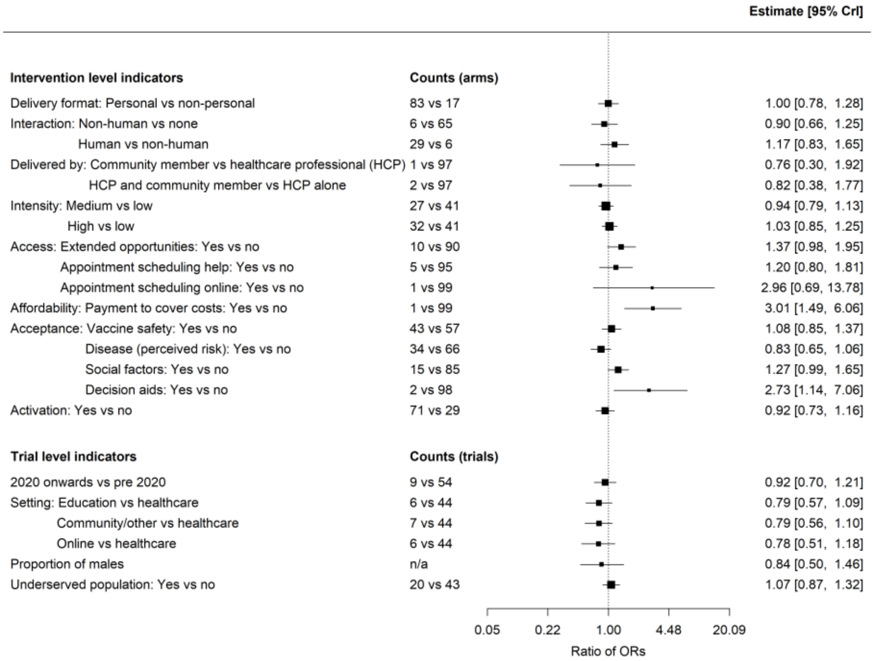

Underserved populations

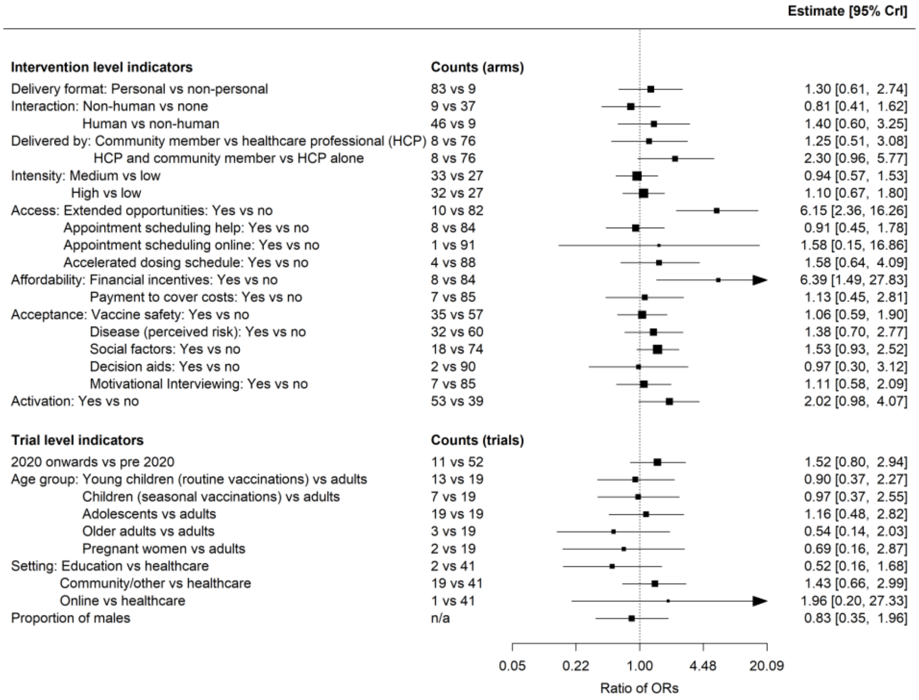

Time period: Pre-2020

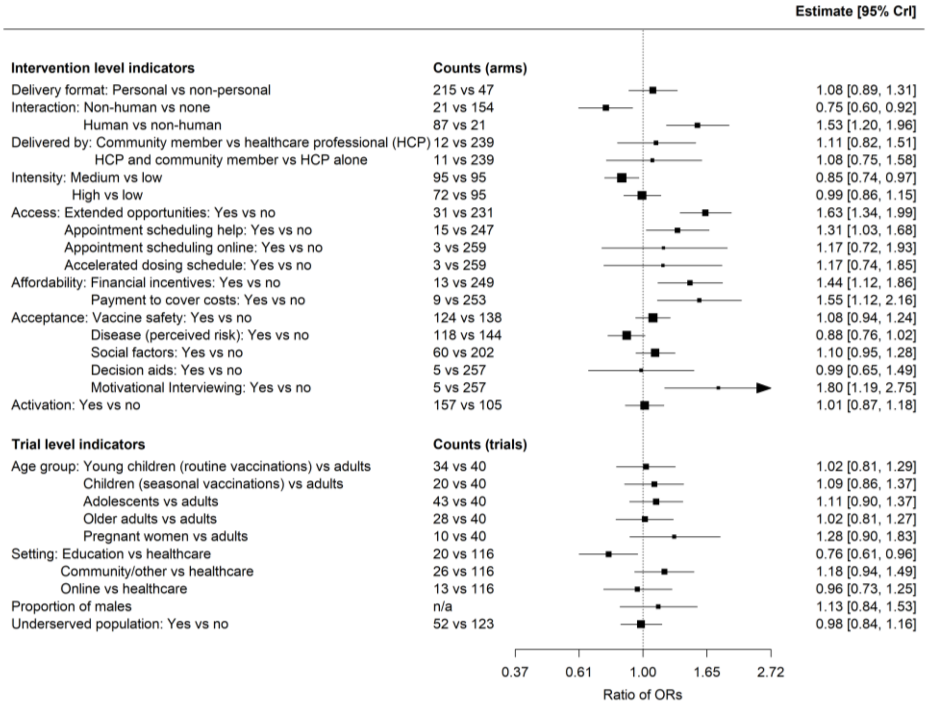

Time period: 2020 onwards

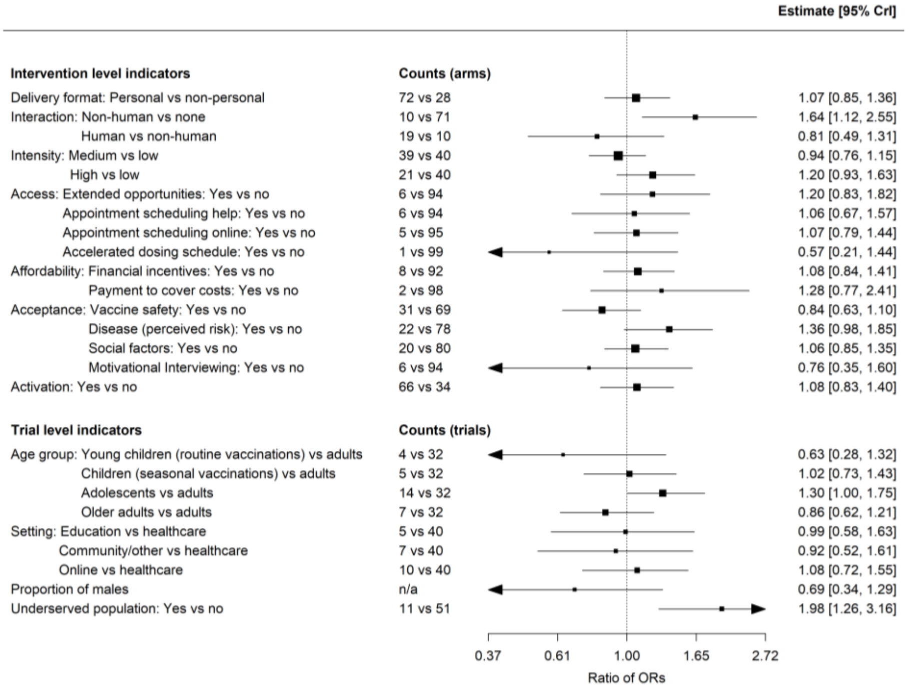

Footnote: We applied the regression model to the subgroups of trials conducted pre-2020 and 2020 onwards using all covariates. However, in the 2020 onwards subgroup, the model failed to converge for the "delivered by" variables (both healthcare professional and community member) due to high correlation between them. Similarly, the model did not converge for decision aids and Pregnant individuals because both variables had only one observation. We therefore excluded these variables from the model for the 2020 onwards subgroup.

K: Sensitivity analyses

Sensitivity analysis: Fixed effects

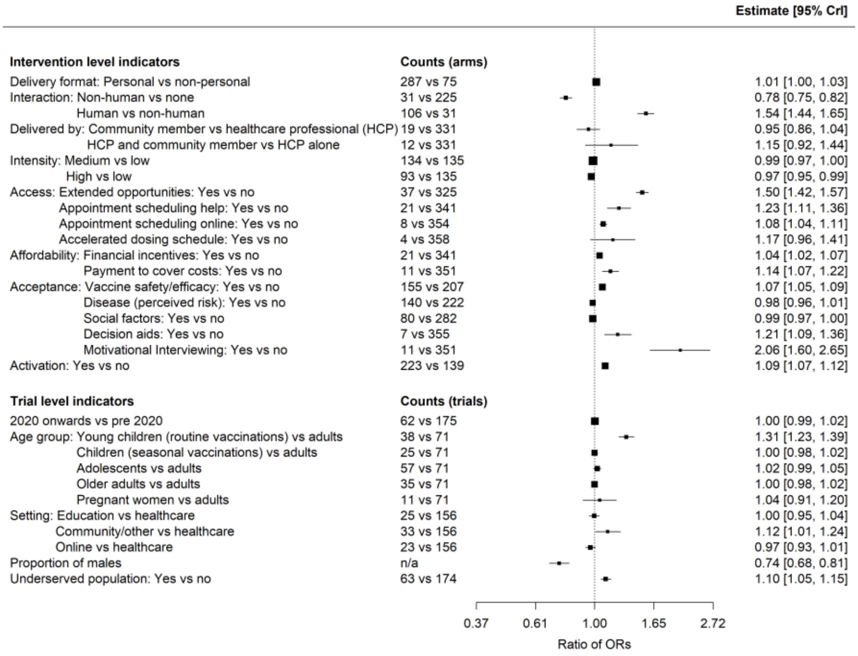

Sensitivity analysis: Removal of studies at high risk of bias

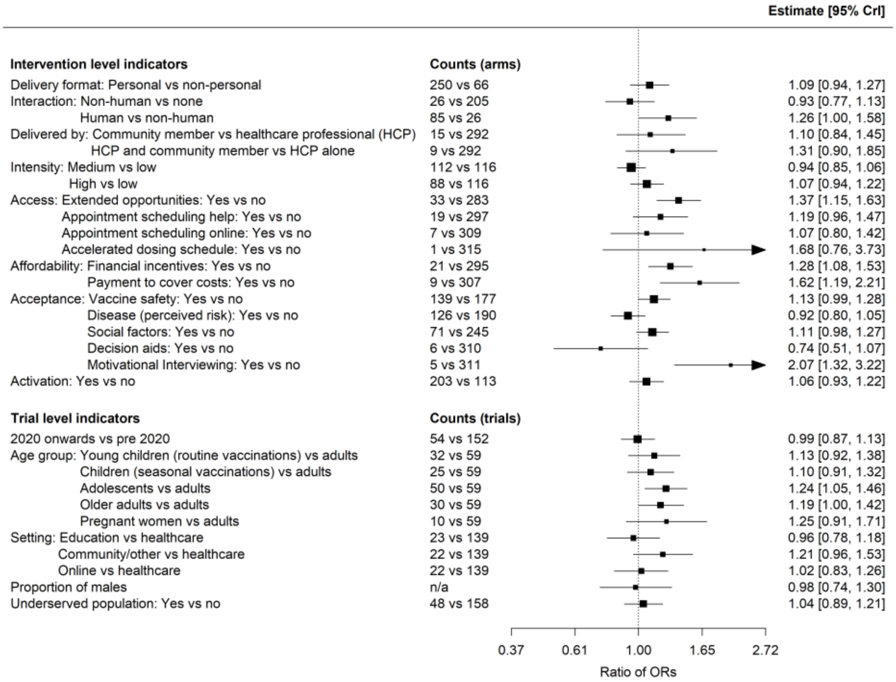

Sensitivity analysis: Removal of outliers

We classified strongly outlying results as those with deviances greater than 2.7. Assuming deviances follow a chi-squared distribution, the probability of a data point having a deviance >2.7 is approximately 10%. Excluding these outliers resulted in the removal of 13 studies (Berset 2022, Dempsey 2019, Ferreira 2022, Goodyear-Smith 2012, Hu 2018, Ma 2018, Ma 2022, Masson 2013, Qin 2023, Scarinci 2020, Shourie 2013, Vivier 2000, Wiseman 2016).

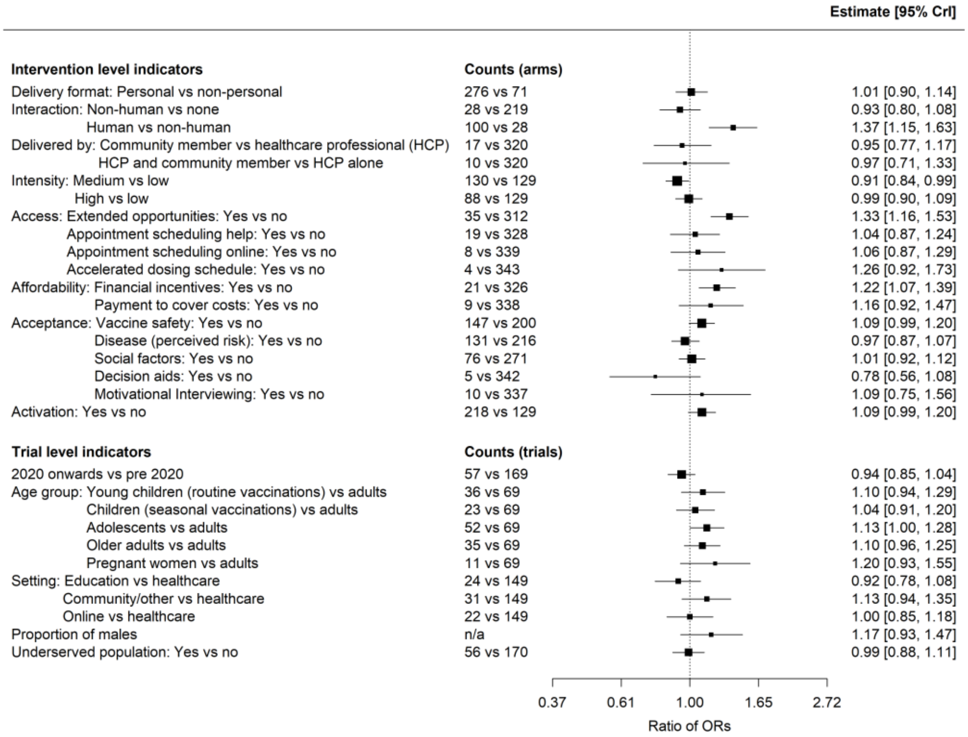

Sensitivity analysis: Cluster adjustments

No cluster adjustments (ICC=0)

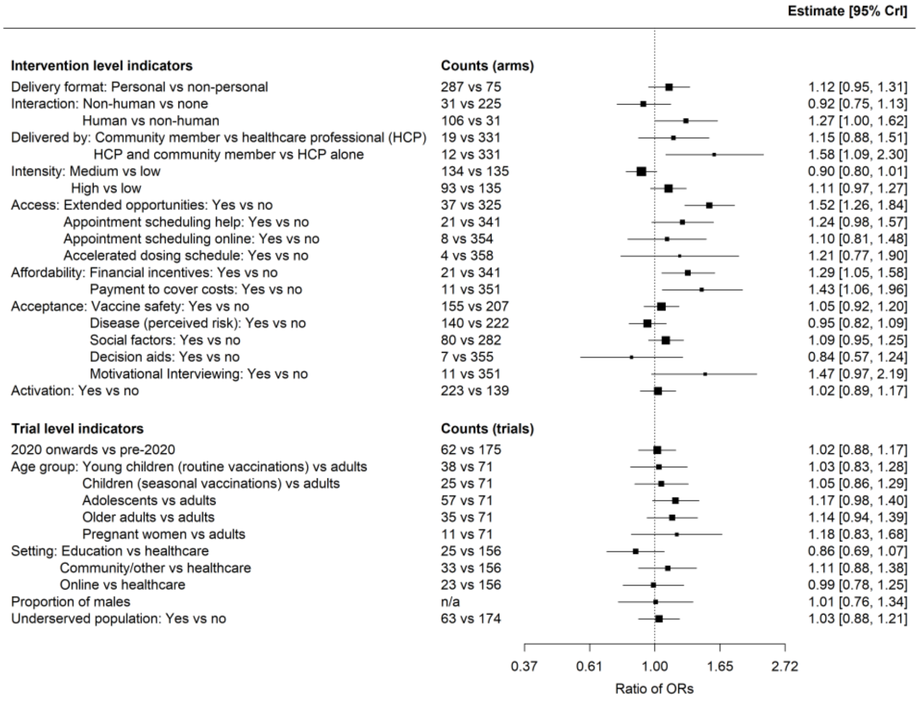

Conservative cluster adjustments (ICC=1 for households and ICC=0.3 otherwise)

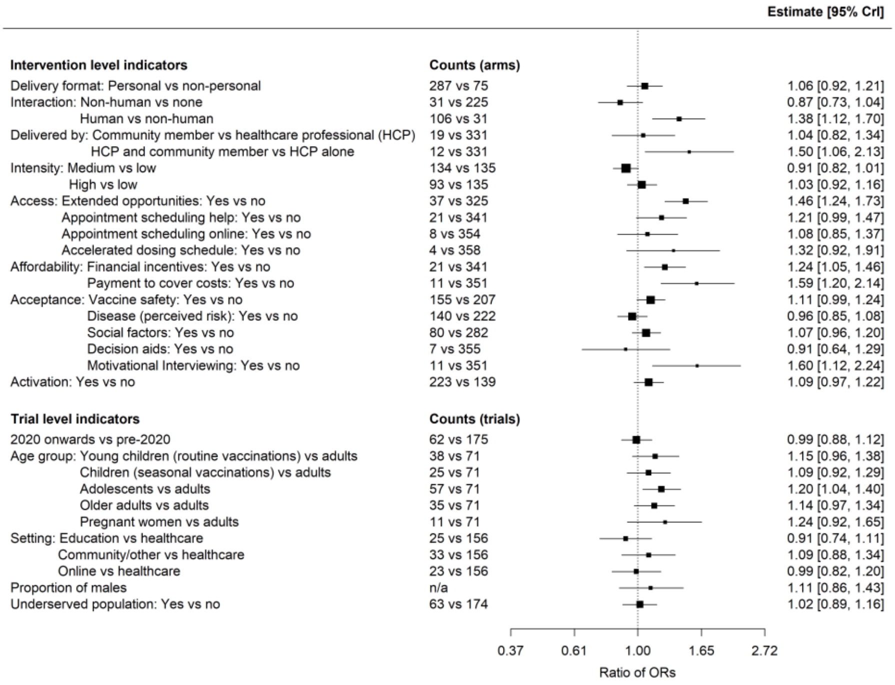

Post-hoc sensitivity analysis: Removal of studies conducted in the United States

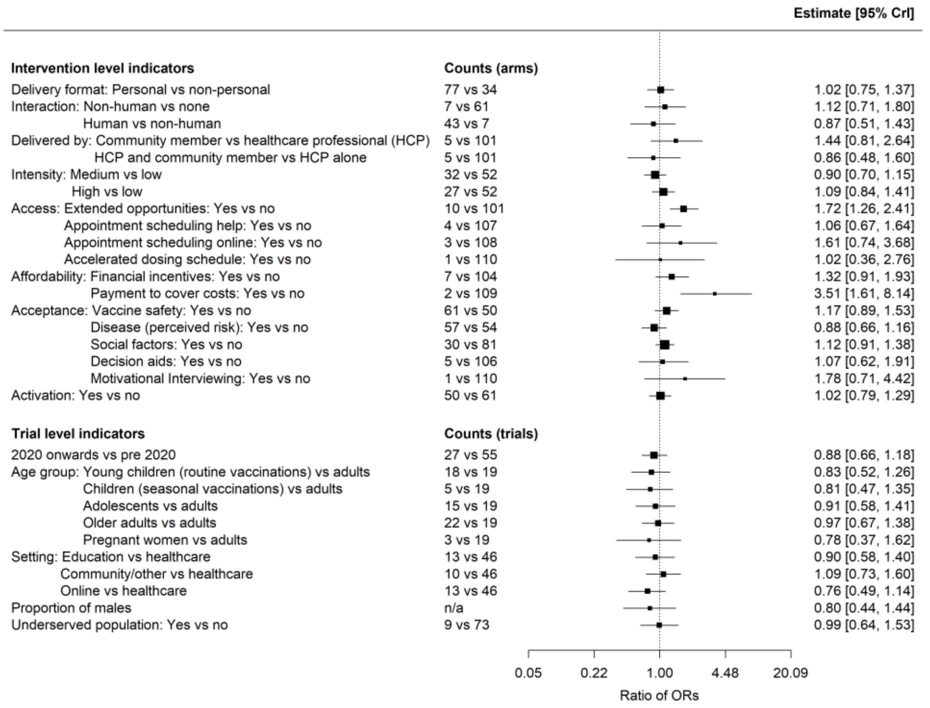

L: Deviations from analysis plan

In our protocol we planned to explore variation in intervention effects by including pairwise interaction terms between indicators. However, our stakeholders were primarily interested in knowing the effect of intervention components within specific populations. We decided this question was best answered by subgroup analyses rather than interaction effects which can be difficult to interpret, especially in multi-level models.

M: References to included studies

1. Abroms LC, Koban D, Krishnan N, et al. Empathic Engagement With the Covid-19 Vaccine Hesitant in Private Facebook Groups: A Randomized Trial. *Health Educ Behav* 2023: 10901981231188313.
2. Alonge OD, Hanson KE, Eggebrecht M, et al. Covid-19 Booster Dose Reminder/Recall for Adolescents: Findings From a Health-Care System in Wisconsin. *J Adolesc Health* 2023; **73**(5): 953-6.
3. Anraad C, van Empelen P, Ruiter RAC, van Keulen H. Effects of an online tailored decision aid to promote informed decision making about maternal pertussis vaccination in the Netherlands: A randomized controlled trial. *Vaccine* 2023; **41**(49): 7348-58.
4. Arnold JN, Gundlach N, Böckelmann I, Sammito S. Randomised Controlled Study on Measures to Increase Vaccination Rates among German Armed Forces Soldiers. *International journal of environmental research and public health* 2022; **19**(14).
5. Arthur AJ, Matthews RJ, Jagger C, Clarke M, Hipkin A, Bennison DP. Improving uptake of influenza vaccination among older people: a randomised controlled trial. *Br J Gen Pract* 2002; **52**(482): 717-8, 20.
6. Bartos V, Bauer M, Cahlikova J, Chytilova J. C ommunicating doctors' consensus persistently increases Covid-19 vaccinations. *Nature* 2022; **606**(7914): 542-9.
7. Bartu A, Sharp J, Ludlow J, Doherty DA. Postnatal home visiting for illicit drug-using mothers and their infants: a randomised controlled trial. *Aust N Z J Obstet Gynaecol* 2006; **46**(5): 419-26.
8. Baskin E. Increasing influenza vaccination rates via low cost messaging interventions. *PLoS One* 2018; **13**(2): 9.

9. Bastani R, Glenn BA, Singhal R, et al. Increasing HPV Vaccination among Low-Income, Ethnic Minority Adolescents: effects of a Multicomponent System Intervention through a County Health Department Hotline. *Cancer epidemiology, biomarkers & prevention* 2022; **31**(1): 175-82.
10. Bennett AT, Patel DA, Carlos RC, et al. Human Papillomavirus Vaccine Uptake After a Tailored, Online Educational Intervention for Female University Students: A Randomized Controlled Trial. *J Womens Health (Larchmt)* 2015; **24**(11): 950-7.
11. Berg GD, Silverstein S, Thomas E, Korn AM. Cost and utilization avoidance with mail prompts: a randomized controlled trial. *Am J Manag Care* 2008; **14**(11): 748-54.
12. Berg GD, Thomas E, Silverstein S, Neel CL, Mireles M. Reducing medical service utilization by encouraging vaccines: randomized controlled trial. *Am J Prev Med* 2004; **27**(4): 284-8.
13. Berkhout C, Willefert-Bouche A, Chazard E, et al. Randomized controlled trial on promoting influenza vaccination in general practice waiting rooms. *PLoS ONE* 2018; **13**(2): e0192155.
14. Bernard-Genest MP, Ruel-Laliberte J, Lapointe-Milot K. Effect of educative reminder telephone calls on human papillomavirus immunization rate: A randomized controlled trial. *Womens Health (Lond Engl)* 2021; **17**: 17455065211003821.
15. Berset AE, Burkhardt MC, Xu Y, Mescher A, Brinkman WB. Effect of Electronic Outreach Using Patient Portal Messages on Well Child Care Visit Completion: A Randomized Clinical Trial. *JAMA Network Open* 2022; **5**(11): e2242853-e.
16. Berset AE, Burkhardt MC, Xu Y, Mescher A, Brinkman WB. Effect of Automated and Personalized Outreach Messages on Well-Child Visit Catch Up: A Randomized Clinical Trial. *Acad Pediatr* 2023; **23**: 23.
17. Bethke N, O'Sullivan JL, Keller J, von Bernuth H, Gellert P, Seybold J. Increasing vaccinations through an on-site school-based education and vaccination program: A city-wide cluster randomized controlled trial. *Appl Psychol Health Well Being* 2024; **01**: 01.
18. Bian J, Guo Z, Zhang W, et al. College students' influence on Covid-19 vaccination uptake among seniors in China: a protocol of combined cross-sectional and experimental study. *BMC Public Health* 2023; **23**(1): 1322.
19. Borg K, Sutton K, Beasley M, et al. Communication-based interventions for increasing influenza vaccination rates among Aboriginal children: a randomised controlled trial. *Vaccine* 2018; **36**(45): 6790-5.
20. Bourgeois FT, Simons WW, Olson K, Brownstein JS, Mandl KD. Evaluation of influenza prevention in the workplace using a personally controlled health record: randomized controlled trial. *J Med Internet Res* 2008; **10**(1): e5.
21. Bowman SE. Hepatitis B vaccination at syringe exchange programs in three US cities: Vaccine efficacy for the standard versus accelerated dosing schedules and determinants of completing the vaccination series among active injection drug users. United States -- Connecticut: Yale University; 2010.
22. Brigham KS, Woods ER, Steltz SK, Sandora TJ, Blood EA. Randomized controlled trial of an immunization recall intervention for adolescents. *Pediatrics* 2012; **130(3)**: 507-14.
23. Bronchetti ET, Huffman DB, Magenheim E. Attention, intentions, and follow-through in preventive health behavior: Field experimental evidence on flu vaccination. *Journal of Economic Behavior & Organization* 2015; **116**: 270-91.
24. Burkhardt MC, Berset AE, Xu Y, Mescher A, Brinkman WB. Effect of Outreach Messages on Adolescent Well-Child Visits and Coronavirus Disease 2019 Vaccine Rates: A Randomized, Controlled Trial. *J Pediatr* 2023; **253**: 158-64.e1.
25. Buttenheim A, Milkman KL, Duckworth AL, Gromet DM, Patel M, Chapman G. Effects of Ownership Text Message Wording and Reminders on Receipt of an Influenza Vaccination: A Randomized Clinical Trial. *JAMA netw* 2022; **5**(2): e2143388.
26. Campos-Mercade P, Meier AN, Schneider FH, Meier S, Pope D, Wengstrom E. Monetary incentives increase Covid-19 vaccinations. *Science* 2021; **374**(6569): 879-+.
27. Cataldi JR, Suresh K, Brewer SE, et al. Boot Camp Translation using Community-Engaged messaging for adolescent Vaccination: a Cluster-Randomized trial. *Vaccine* 2024; **42**(5): 1078-86.
28. Centers for Disease Control and Prevention. Evaluation of vaccination recall letter system for Medicaid-enrolled children aged 19-23 months--Montana, 2011. *MMWR Morb Mortal Wkly Rep* 2012; **61**(40): 811-5.
29. Chai SJ, Tan F, Ji Y, Wei X, Li R, Frost M. Community-level text messaging for 2009 H1N1 prevention in China. *American journal of preventive medicine* 2013; **45**(2): 190-6.
30. Chang TY, Jacobson M, Shah M, Kopetsky M, Pramanik R, Shah SB. Reminders, but not monetary incentives, increase Covid-19 booster uptake. *Proc Natl Acad Sci U S A* 2023; **120**(31): e2302725120.
31. Chao C, Preciado M, Slezak J, Xu LF. A Randomized Intervention of Reminder Letter for Human Papillomavirus Vaccine Series Completion. *J Adolesc Health* 2015; **56**(1): 85-90.
32. Chodick G, Teper GR, Levi S, et al. The impact of a Facebook campaign among mothers on HPV vaccine uptake among their daughters: A randomized field study. *Gynecol Oncol* 2021; **160**(1): 106-11.

33. Clayton K, Finley C, Flynn DJ, Graves M, Nyhan B. Evaluating the effects of vaccine messaging on immunization intentions and behavior: Evidence from two randomized controlled trials in Vermont. *Vaccine* 2021; **39**(40): 5909-17.
34. Coley S, Hoefer D, Rausch-Phung E. A population-based reminder intervention to improve human papillomavirus vaccination rates among adolescents at routine vaccination age. *Vaccine* 2018; **36**(32): 4904-9.
35. Conner M, Sandberg T, Nekitsing C, et al. Varying cognitive targets and response rates to enhance the question-behaviour effect: An 8-arm Randomized Controlled Trial on influenza vaccination uptake. *Social Science & Medicine* 2017; **180**: 135-42.
36. Cox AD, Cox D, Cyrier R, Graham-Dotson Y, Zimet GD. Can Self-Prediction Overcome Barriers to Hepatitis B Vaccination? A Randomized Controlled Trial. *Health Psychol* 2012; **31**(1): 97-105.
37. Cutrona SL, Golden JG, Goff SL, et al. Improving Rates of Outpatient Influenza Vaccination Through EHR Portal Messages and Interactive Automated Calls: A Randomized Controlled Trial. *J Gen Intern Med* 2018; **33**(5): 659-67.
38. Dai H, Saccardo S, Han MA, et al. Behavioural nudges increase Covid-19 vaccinations. *Nature* 2021; **597**(7876): 404-9.
39. Dalby DM, Sellors JW, Fraser FD, Fraser C, van Ineveld C, Howard M. Effect of preventive home visits by a nurse on the outcomes of frail elderly people in the community: a randomized controlled trial. *Cmaj* 2000; **162**(4): 497-500.
40. Daley MF, Kempe A, Pyrzanowski J, et al. School-located vaccination of adolescents with insurance billing: cost, reimbursement, and vaccination outcomes. *J Adolesc Health* 2014; **54**(3): 282-8.
41. Daley MF, Steiner JF, Brayden RM, Xu S, Morrison S, Kempe A. Immunization registry-based recall for a new vaccine. *Ambulatory pediatrics* 2002; **2**(6): 438-43.
42. Daniels NA, Juarbe T, Moreno-John G, Perez-Stable EJ. Effectiveness of adult vaccination programs in faith-based organizations. *Ethn Dis* 2007; **17**(1 Suppl 1): S15-22.
43. Dapp U, Anders JA, von Renteln-Kruse W, et al. A randomized trial of effects of health risk appraisal combined with group sessions or home visits on preventive behaviors in older adults. *J Gerontol A Biol Sci Med Sci* 2011; **66**(5): 591-8.
44. Davies C, Skinner SR, Stoney T, et al. 'Is it like one of those infectious kind of things?' the importance of educating young people about HPV and HPV vaccination at school. *Sex Education* 2017; **17**(3): 256-75.
45. DeCamp LR, Godage SK, Valenzuela Araujo D, et al. A Texting Intervention in Latino Families to Reduce ED Use: A Randomized Trial. *Pediatrics* 2020; **145**(1): 01.
46. Dempsey AF, Maertens J, Sevick C, Jimenez-Zambrano A, Juarez-Colunga E. A randomized, controlled, pragmatic trial of an iPad-based, tailored messaging intervention to increase human papillomavirus vaccination among Latinos. *Human Vaccines Immunother* 2019; **15**(7-8): 1577-84.
47. DiClemente RJ, Murray CC, Graham T, Still J. Overcoming barriers to HPV vaccination: A randomized clinical trial of a culturally-tailored, media intervention among African American girls. *Hum Vaccin Immunother* 2015; **11**(12): 2883-94.
48. Dini EF, Linkins RW, Sigafoos J. The impact of computer-generated messages on childhood immunization coverage. *Am J Prev Med* 2000; **18**(2): 132-9.
49. Dombkowski KJ, Costello LE, Harrington LB, Dong S, Kolasa M, Clark SJ. Age-specific strategies for immunization reminders and recalls: A registry-based randomized trial. *American Journal of Preventive Medicine* 2014; **47(1)**: 1-8.
50. Dombkowski KJ, Cowan AE, Reeves SL, Foley MR, Dempsey AF. The impacts of email reminder/recall on adolescent influenza vaccination. *Vaccine* 2017; **35**(23): 3089-95.
51. Domek GJ, Contreras-Roldan IL, Bull S, et al. Text message reminders to improve infant immunization in Guatemala: A randomized clinical trial. *Vaccine* 2019; **37**(42): 6192-200.
52. Doyle O, Fitzpatrick N, Lovett J, Rawdon C. Early intervention and child physical health: Evidence from a Dublin-based randomized controlled trial. *Econ Hum Biol* 2015; **19**: 224-45.
53. El-Mohandes AA, Katz KS, El-Khorazaty MN, et al. The effect of a parenting education program on the use of preventive pediatric health care services among low-income, minority mothers: a randomized, controlled study. *Pediatrics* 2003; **111**(6 Pt 1): 1324-32.
54. Esposito S, Bianchini S, Tagliabue C, et al. Impact of a website based educational program for increasing vaccination coverage among adolescents. *Hum Vaccin Immunother* 2018; **14**(4): 961-8.
55. Fernandez ME, Savas LS, Atkinson JS, et al. Evaluation of a 2-1-1 Telephone Navigation Program to Increase Cancer Control Behaviors: Results From a Randomized Controlled Trial. *Am J Health Promot* 2022; **36**(7): 1083-93.
56. Ferreira H, Siqueira CM, Sousa LB, et al. Effect of educational intervention for compliance of school adolescents with the human papillomavirus vaccine. *Revista da Escola de Enfermagem da USP* 2022; **56**: e20220082.

57. Fiks AG, Grundmeier RW, Mayne S, et al. Effectiveness of decision support for families, clinicians, or both on HPV vaccine receipt. *Pediatrics* 2013; **131(6)**: 1114-24.
58. Fitzpatrick T, Zhou KL, Cheng Y, et al. A crowdsourced intervention to promote hepatitis B and C testing among men who have sex with men in China: study protocol for a nationwide online randomized controlled trial. *BMC Infect Dis* 2018; **18**: 9.
59. Frew PM, Kriss JL, Chamberlain AT, et al. A randomized trial of maternal influenza immunization decision-making: A test of persuasive messaging models. *Human Vaccines and Immunotherapeutics* 2016; **12(8)**: 1989-96.
60. Gerend MA, Madkins K, Crosby S, et al. Evaluation of a Text Messaging-Based Human Papillomavirus Vaccination Intervention for Young Sexual Minority Men: Results from a Pilot Randomized Controlled Trial. *Ann Behav Med* 2021; **55**(4): 321-32.
61. Glanz JM, Wagner NM, Narwaney KJ, et al. Web-based Social Media Intervention to Increase Vaccine Acceptance: A Randomized Controlled Trial. *Pediatrics* 2017; **140**(6): 1-9.
62. Glanz JM, Wagner NM, Narwaney KJ, et al. Web-Based Tailored Messaging to Increase Vaccination: A Randomized Clinical Trial. *Pediatrics* 2020; **146**(5): 1-10.
63. Goodman K, Mossad SB, Taksler GB, Emery J, Schramm S, Rothberg MB. Impact of Video Education on Influenza Vaccination in Pregnancy. *J Reprod Med* 2015; **60**(11-12): 471-9.
64. Goodyear-Smith F, Grant C, Poole T, et al. Early connections: effectiveness of a pre-call intervention to improve immunisation coverage and timeliness. *J Prim Health Care* 2012; **4**(3): 189-98.
65. Grandahl M, Rosenblad A, Stenhammar C, et al. School-based intervention for the prevention of HPV among adolescents: a cluster randomised controlled study. *BMJ Open* 2016; **6**(1): e009875.
66. Gurfinkel D, Kempe A, Albertin C, et al. Centralized reminder/recall for human papillomavirus vaccination: Findings from two states-A randomized clinical trial. *Journal of Adolescent Health* 2021; **69**(4): 579-87.
67. Haff N, Choudhry NK, Bhatkhande G, et al. "How" Versus "Why" Messaging to Increase Uptake of Booster Vaccination Against Covid-19: Results of a Pragmatic Randomized Trial. *J Gen Intern Med* 2023; **06**: 06.
68. Hambidge SJ, Phibbs SL, Chandramouli V, Fairclough D, Steiner JF. A stepped intervention increases well-child care and immunization rates in a disadvantaged population. *Pediatrics* 2009; **124**(2): 455-64.
69. Hanley K, Chung TH, Nguyen LK, et al. Using Electronic Reminders to Improve Human Papillomavirus (HPV) Vaccinations among Primary Care Patients. *Vaccines* 2023; **11(4) (no pagination)**.
70. Hannan J. APN telephone follow up to low-income first time mothers. *J Clin Nurs* 2013; **22**(1-2): 262-70.
71. Harari D, Iliffe S, Kharicha K, et al. Promotion of health in older people: a randomised controlled trial of health risk appraisal in British general practice. *Age Ageing* 2008; **37**(5): 565-71.
72. Henrikson NB, Zhu W, Baba L, et al. Outreach and Reminders to Improve Human Papillomavirus Vaccination in an Integrated Primary Care System. *Clin Pediatr (Phila)* 2018; **57**(13): 1523-31.
73. Hess R. Impact of automated telephone messaging on zoster vaccination rates in community pharmacies. *J Am Pharm Assoc (2003)* 2013; **53**(2): 182-7.
74. Higginbotham S, Stewart A, Pfalzgraf A. Impact of a pharmacist immunizer on adult immunization rates. *J Am Pharm Assoc (2003)* 2012; **52**(3): 367-71.
75. Ho HJ, Tan YR, Cook AR, et al. Increasing Influenza and Pneumococcal Vaccination Uptake in Seniors Using Point-of-Care Informational Interventions in Primary Care in Singapore: A Pragmatic, Cluster-Randomized Crossover Trial. *Am J Public Health* 2019; **109**(12): 1776-83.
76. Hofstetter AM, DuRivage N, Vargas CY, et al. Text message reminders for timely routine MMR vaccination: A randomized controlled trial. *Vaccine* 2015; **33**(43): 5741-6.
77. Hofstetter AM, Vargas CY, Camargo S, et al. Impacting delayed pediatric influenza vaccination: a randomized controlled trial of text message reminders. *American journal of preventive medicine* 2015; **48**(4): 392-401.
78. Hopfer S. Effects of a narrative HPV vaccination intervention aimed at reaching college women: a randomized controlled trial. *Prev Sci* 2012; **13**(2): 173-82.
79. Howell-Jones R, Gold N, Bowen S, et al. Can uptake of childhood influenza immunisation through schools and GP practices be increased through behaviourally-informed invitation letters and reminders: two pragmatic randomized controlled trials. *BMC Public Health* 2023; **23**(1): 143.
80. Hu PL, Koh EYL, Tay JSH, Chan VX, Goh SSM, Wang SZ. Assessing the impact of educational methods on influenza vaccine uptake and patient knowledge and attitudes: a randomised controlled trial. *Singapore Med J* 2021; **15**: 15.
81. Hu Y, Chen Y, Wang Y, Song Q, Li Q. Prenatal vaccination education intervention improves both the mothers' knowledge and children's vaccination coverage: Evidence from randomized controlled trial from eastern China. *Hum Vaccin Immunother* 2017; **13**(6): 1-8.

82. Hu Y, Li Q, Chen Y. Evaluation of two health education interventions to improve the varicella vaccination: a randomized controlled trial from a province in the east China. *BMC Public Health* 2018; **18**(1): 144.
83. Huf SW, Grailey K, Crespo RF, et al. Testing the impact of differing behavioural science informed text message content in Covid-19 vaccination invitations on vaccine uptake: A randomised clinical trial. *Vaccine* 2024; **28**: 28.
84. Hull S, Hagdrup N, Hart B, Griffiths C, Hennessy E. Boosting uptake of influenza immunisation: a randomised controlled trial of telephone appointing in general practice. *Br J Gen Pract* 2002; **52**(482): 712-6.
85. Humiston SG, Schaffer SJ, Szilagyi PG, et al. Seasonal influenza vaccination at school: a randomized controlled trial. *Am J Prev Med* 2014; **46**(1): 1-9.
86. Hurley LP, Beaty B, Gurfinkel D, Lockhart S, Miriam Dickinson L, Kempe A. Randomized controlled trial of centralized vac-cine reminder/recall to improve adult vaccination rates in an accountable care organization setting. *Journal of General Internal Medicine* 2018; **33(2 Supplement 1)**: 324.
87. Hurley LP, Beaty B, Lockhart S, et al. RCT of Centralized Vaccine Reminder/Recall for Adults. *Am J Prev Med* 2018; **55**(2): 231-9.
88. Hurley LP, Beaty B, Lockhart S, et al. Randomized controlled trial of centralized vaccine reminder/recall to improve adult vaccination rates in an accountable care organization setting. *Preventive medicine reports* 2019; **15**.
89. Hurtaud A, Coomans C, Vuillemin B, et al. Impact of a dTcaP booster vaccine awareness campaign initiated by the French national health insurance for adults aged 25 years in 2021. *BMC Health Serv Res* 2023; **23**(1): 903.
90. Hwang LY, Grimes CZ, Tran TQ, et al. Accelerated hepatitis B vaccination schedule among drug users: a randomized controlled trial. *J Infect Dis* 2010; **202**(10): 1500-9.
91. Irigoyen MM, Findley S, Wang D, et al. Challenges and Successes of Immunization Registry Reminders at Inner-City Practices. *Ambulatory Pediatrics* 2006; **6(2)**: 100-4.
92. Isrctn. The effects of monetary incentives on Covid-19 vaccination uptake. *https://trialsearchwhoint/Trial2aspx?TrialID=ISRCTN59503725* 2021.
93. Jackson C, Cheater FM, Harrison W, et al. Randomised cluster trial to support informed parental decision-making for the MMR vaccine. *BMC Public Health* 2011; **11**: 11.
94. Janitz AE, Neil JM, Bray LA, et al. CATCH-UP vaccines: protocol for a randomized controlled trial using the multiphase optimization strategy (MOST) framework to evaluate education interventions to increase Covid-19 vaccine uptake in Oklahoma. *BMC Public Health* 2023; **23**(1): 1146.
95. Jiang M, Yao X, Li P, et al. Impact of video-led educational intervention on uptake of influenza vaccine among the elderly in western China: a community-based randomized controlled trial. *BMC Public Health* 2022; **22**(1): 1128.
96. Johansen ND, Vaduganathan M, Bhatt AS, et al. Electronic nudges to increase influenza vaccination uptake among patients with heart failure: a prespecified analysis of the NUDGE-FLU trial. *European journal of heart failure* 2023.
97. Johnson EA, Harwell TS, Donahue PM, et al. Promoting pneumococcal immunizations among rural Medicare beneficiaries using multiple strategies. *J Rural Health* 2003; **19**(4): 506-10.
98. Jordan ET, Bushar JA, Kendrick JS, Johnson P, Wang J. Encouraging Influenza Vaccination Among Text4baby Pregnant individuals and Mothers. *Am J Prev Med* 2015; **49**(4): 563-72.
99. Joseph NP, Bernstein J, Pelton S, et al. Brief Client-Centered Motivational and Behavioral Intervention to Promote HPV Vaccination in a Hard-to-Reach Population. *Clinical Pediatrics* 2016; **55**(9): 851-9.
100. Ju Q, Xiao H, Peng H, Gan Y. How to Improve People's Intentions Regarding Covid-19 Vaccination in China: A Randomized Controlled Trial. *Int J Behav Med* 2024; **20**: 20.
101. Juon HS, Strong C, Kim F, Park E, Lee S. Lay Health Worker Intervention Improved Compliance with Hepatitis B Vaccination in Asian Americans: Randomized Controlled Trial. *PLoS ONE* 2016; **11**(9): e0162683.
102. Juraskova I, Bari RA, O'Brien MT, McCaffery KJ. HPV Vaccine Promotion: Does Referring to Both Cervical Cancer and Genital Warts Affect Intended and Actual Vaccination Behavior? *Womens Health Iss* 2011; **21**(1): 71-9.
103. Kasting ML, Head KJ, Cox D, Cox AD, Zimet GD. The effects of message framing and healthcare provider recommendation on adult hepatitis B vaccination: A randomized controlled trial. *Preventive Medicine* 2019; **127 (no pagination)**(105798).
104. Kempe A, Barrow J, Stokley S, et al. Effectiveness and cost of immunization recall at school-based health centers. *Pediatrics* 2012; **129**(6): e1446-52.
105. Kempe A, Daley MF, Barrow J, et al. Implementation of universal influenza immunization recommendations for healthy young children: results of a randomized, controlled trial with registry-based recall. *Pediatrics* 2005; **115**(1): 146-54.

106. Kempe A, Lowery NE, Pearson KA, et al. Immunization recall: effectiveness and barriers to success in an urban teaching clinic. *J Pediatr* 2001; **139**(5): 630-5.
107. Kempe A, O'Leary ST, Shoup JA, et al. Parental Choice of Recall Method for HPV Vaccination: A Pragmatic Trial. *Pediatrics* 2016; **137**(3): 26-.
108. Kempe A, Saville AW, Albertin C, et al. Centralized Reminder/Recall to Increase Influenza Vaccination Rates: A Two-State Pragmatic Randomized Trial. *Academic Pediatrics* 2020; **20**(3): 374-83.
109. Kerpelman LC, Connell DB, Gunn WJ. Effect of a monetary sanction on immunization rates of recipients of aid to families with dependent children. *JAMA* 2000; **284**(1): 53-9.
110. Khan AA, Tran HN, Lai JA, et al. A Learning Health System Approach to Increasing Human Papillomavirus Immunizations Among Young Adults. *Perm* 2023; **27**(2): 31-6.
111. Kim M. "I want to know more about the HPV vaccine": Stories by Korean American college women. *Dissertation Abstracts International: Section B: The Sciences and Engineering* 2018; **79**(4-B(E)): No-Specified.
112. Krieger JW, Castorina JS, Walls ML, Weaver MR, Ciske S. Increasing influenza and pneumococcal immunization rates: a randomized controlled study of a senior center-based intervention. *Am J Prev Med* 2000; **18**(2): 123-31.
113. Kulle A-C, Schumacher S, Bieberstein Fv. Mobile vaccination units substantially increase Covid-19 vaccinations: evidence from a randomized controlled trial. *Journal of Public Health* 2024; **46**(1): 151-7.
114. Lau AY, Sintchenko V, Crimmins J, Magrabi F, Gallego B, Coiera E. Impact of a web-based personally controlled health management system on influenza vaccination and health services utilization rates: a randomized controlled trial. *J Am Med Inform Assoc* 2012; **19**(5): 719-27.
115. LeBaron CW, Starnes DM, Rask KJ. The impact of reminder-recall interventions on low vaccination coverage in an inner-city population. *Arch Pediatr Adolesc Med* 2004; **158**(3): 255-61.
116. Lee WN, Stuck D, Konty K, et al. Large-scale influenza vaccination promotion on a mobile app platform: A randomized controlled trial. *Vaccine* 2020; **38**(18): 3508-14.
117. Lerner C, Albertin C, Casillas A, et al. Patient portal reminders for pediatric influenza vaccinations: A randomized clinical trial. *Pediatrics* 2021; **148(2) (no pagination)**.
118. Leung KC, Mui C, Chiu WY, et al. Impact of patient education on influenza vaccine uptake among community-dwelling elderly: a randomized controlled trial. *Health Educ Res* 2017; **32**(5): 455-64.
119. Liao Q, Fielding R, Cheung YTD, Lian J, Yuan J, Lam WWT. Effectiveness and Parental Acceptability of Social Networking Interventions for Promoting Seasonal Influenza Vaccination Among Young Children: randomized Controlled Trial. *Journal of medical Internet research* 2020; **22**(2): e16427.
120. Lieu TA, Elkin EP, Escobar PR, et al. Effect of Electronic and Mail Outreach From Primary Care Physicians for Covid-19 Vaccination of Black and Latino Older Adults: A Randomized Clinical Trial. *JAMA netw* 2022; **5**(6): e2217004.
121. Lin SC, Tam KW, Yen JYC, et al. The impact of shared decision making with patient decision aids on the rotavirus vaccination rate in children: A randomized controlled trial. *Preventive Medicine* 2020; **141 (no pagination)**(106244).
122. Ma GX, Lee MM, Tan Y, et al. Efficacy of a community-based participatory and multilevel intervention to enhance hepatitis B virus screening and vaccination in underserved Korean Americans. *Cancer* 2018; **124**(5): 973-82.
123. Ma GX, Zhu L, Tan Y, et al. A Multilevel Intervention to Increase HPV Vaccination among Asian American Adolescents. *J Community Health* 2022; **47**(1): 9-16.
124. Mantzari E, Vogt F, Marteau TM. Financial incentives for increasing uptake of HPV vaccinations: A randomized controlled trial. *Health Psychology* 2015; **34(2)**: 160-71.
125. Mason BW, Donnelly PD. Targeted mailing of information to improve uptake of measles, mumps, and rubella vaccine: a randomised controlled trial. *Commun Dis Public Health* 2000; **3**(1): 67-8.
126. Masson CL, Delucchi KL, McKnight C, et al. A randomized trial of a hepatitis care coordination model in methadone maintenance treatment. *Am J Public Health* 2013; **103**(10): e81-8.
127. McCaul KD, Johnson RJ, Rothman AJ. The effects of framing and action instructions on whether older adults obtain flu shots. *Health Psychol* 2002; **21**(6): 624-8.
128. Meharry PM. Maternal influenza vaccination strategies to improve vaccine uptake in pregnancy. *Dissertation Abstracts International: Section B: The Sciences and Engineering* 2014; **74**(9-B(E)): No-Specified.
129. Mehta SJ, Mallozzi C, Shaw PA, et al. Effect of Text Messaging and Behavioral Interventions on Covid-19 Vaccination Uptake: A Randomized Clinical Trial. *JAMA netw* 2022; **5**(6): e2216649.
130. Menzies R, Heron L, Lampard J, et al. A randomised controlled trial of SMS messaging and calendar reminders to improve vaccination timeliness in infants. *Vaccine* 2020; **38**(15): 3137-42.
131. Moniz MH, Hasley S, Meyn LA, Beigi RH. Improving influenza vaccination rates in pregnancy through text messaging: a randomized controlled trial. *Obstet Gynecol* 2013; **121**(4): 734-40.
132. Munoz-Miralles R, Nadeu SB, Masoliver CS, et al. Original Effectiveness of a brief intervention for acceptance of influenza vaccine in reluctant primary care patients. *Gac Sanit* 2022; **36**(5): 446-51.

133. Nct. Lottery Incentive Nudges to Increase Influenza Vaccinations. *https://clinicaltrialsgov/show/NCT05012163* 2021.
134. Nct. SMS Reminders to Strengthen Demand for HPV Vaccination in Georgia. *https://clinicaltrialsgov/show/NCT05536674* 2022.
135. Nct. Vale+ Tu Salud: corner-Based Randomized Trial to Test a Latino Day Laborer Program Adapted to Prevent COVID 19. *https://clinicaltrialsgov/show/NCT05248399* 2022.
136. Nct. Motivation, Syringe Exchange, and Covid-19. *https://clinicaltrialsgov/ct2/show/NCT05534061* 2022.
137. Nct. Precision Vaccine Promotion in Underserved Populations. *https://clinicaltrialsgov/ct2/show/NCT05537441* 2022.
138. Nehme EK, Delphia M, Cha EM, Thomas M, Lakey D. Promoting Influenza Vaccination Among an ACA Health Plan Subscriber Population: A Randomized Trial. *Am J Health Promot* 2019; **33**(6): 916-20.
139. Nowalk MP, Lin CJ, Toback SL, et al. Improving influenza vaccination rates in the workplace: a randomized trial. *Am J Prev Med* 2010; **38**(3): 237-46.
140. Nyamathi A, Liu Y, Marfisee M, et al. Effects of a nurse-managed program on hepatitis A and B vaccine completion among homeless adults. *Nurs Res* 2009; **58**(1): 13-22.
141. Nyamathi A, Sinha K, Greengold B, Cohen A, Marfisee M. Predictors of HAV/HBV vaccination completion among methadone maintenance clients. *Res Nurs Health* 2010; **33**(2): 120-32.
142. O'Grady KF, Kaus M, Jones L, et al. SMS reminders to improve the uptake and timeliness of the primary immunisation series in infants: a multi-centre randomised controlled trial. *Commun Dis Intell (2018)* 2022; **46**: 19.
143. O'Leary ST, Lee M, Lockhart S, et al. Effectiveness and Cost of Bidirectional Text Messaging for Adolescent Vaccines and Well Care. *Pediatrics* 2015; **136**(5): e1220-7.
144. O'Leary ST, Narwaney KJ, Wagner NM, Kraus CR, Omer SB, Glanz JM. Efficacy of a Web-Based Intervention to Increase Uptake of Maternal Vaccines: An RCT. *Am J Prev Med* 2019; **57**(4): e125-e33.
145. Omer SB, O'Leary ST, Bednarczyk RA, et al. Multi-tiered intervention to increase maternal immunization coverage: A randomized, controlled trial. *Vaccine* 2022; **40**(34): 4955-63.
146. Osborne MT, Kenah E, Lancaster K, Tien J. Catch the tweet to fight the flu: Using Twitter to promote flu shots on a college campus. *J Am Coll Health* 2023; **71**(8): 2470-84.
147. Otsuka SH, Tayal NH, Porter K, Embi PJ, Beatty SJ. Improving herpes zoster vaccination rates through use of a clinical pharmacist and a personal health record. *Am J Med* 2013; **126**(9): 832.e1-6.
148. Otsuka-Ono H, Hori N, Ohta H, Uemura Y, Kamibeppu K. A childhood immunization education program for parents delivered during late pregnancy and one-month postpartum: a randomized controlled trial. *BMC Health Serv Res* 2019; **19**(1): 798.
149. Patel A, Stern L, Unger Z, et al. Staying on track: a cluster randomized controlled trial of automated reminders aimed at increasing human papillomavirus vaccine completion. *Vaccine* 2014; **32**(21): 2428-33.
150. Patel MS, Milkman KL, Gandhi L, et al. A Randomized Trial of Behavioral Nudges Delivered Through Text Messages to Increase Influenza Vaccination Among Patients With an Upcoming Primary Care Visit. *Am J Health Promot* 2023; **37**(3): 324-32.
151. Porter-Jones G, Williams S, Powell C, Pusey L, Roberts RJ. Impact of a novel way to communicate information about MMR on uptake of MMR vaccine: A randomized controlled trial. *Public Health* 2009; **123**(1): 78-80.
152. Pot M, Paulussen T, Ruiter RAC, et al. Effectiveness of a Web-Based Tailored Intervention With Virtual Assistants Promoting the Acceptability of HPV Vaccination Among Mothers of Invited Girls: Randomized Controlled Trial. *J Med Internet Res* 2017; **19**(9): 18.
153. Qin C, Li Y, Qiu S, et al. Pay-it-forward to increase uptake among 15-18-year-old adolescent girls compared with user-paid vaccination: The pilot results of a two-arm randomized controlled trial in China. *Res Sq* 2023; **25**: 25.
154. Quinlivan JA, Box H, Evans SF. Postnatal home visits in teenage mothers: a randomised controlled trial. *Lancet* 2003; **361**(9361): 893-900.
155. Rand CM, Brill H, Albertin C, et al. Effectiveness of centralized text message reminders on human papillomavirus immunization coverage for publicly insured adolescents. *Journal of Adolescent Health* 2015; **56**(5, Suppl): S17-S20.
156. Rand CM, Vincelli P, Goldstein NPN, Blumkin A, Szilagyi PG. Effects of phone and text message reminders on completion of the human papillomavirus vaccine series. *Journal of Adolescent Health* 2017; **60**(1): 113-9.
157. Reiter PL, Gower AL, Kiss DE, et al. Efficacy of the Outsmart HPV Intervention: A Randomized Controlled Trial to Increase HPV Vaccination among Young Gay, Bisexual, and Other Men Who Have Sex with Men. *Cancer Epidemiol Biomarkers Prev* 2023; **32**(6): 760-7.

158. Reiter PL, Katz ML, Bauermeister JA, Shoben AB, Paskett ED, McRee A-L. Increasing Human Papillomavirus Vaccination Among Young Gay and Bisexual Men: A Randomized Pilot Trial of the Outsmart HPV Intervention. *LGBT Health* 2018; **5**(5): 325-9.
159. Richman AR, Maddy L, Torres E, Goldberg EJ. A randomized intervention study to evaluate whether electronic messaging can increase human papillomavirus vaccine completion and knowledge among college students. *J Am Coll Health* 2016; **64**(4): 269-78.
160. Richman AR, Torres E, Wu Q, et al. Text and Email Messaging for Increasing Human Papillomavirus Vaccine Completion among Uninsured or Medicaid-insured Adolescents in Rural Eastern North Carolina. *J Health Care Poor Underserved* 2019; **30**(4): 1499-517.
161. Roca B, Herrero E, Resino E, Torres V, Penades M, Andreu C. Impact of education program on influenza vaccination rates in Spain. *Am J Manag Care* 2012; **18**(12): e446-52.
162. Rodriguez R, Nichol G, Eucker S, et al. 4 Covid-19 Vaccine Messaging Platforms Increase Vaccine Acceptance and Uptake in Unvaccinated Emergency Department Patients: A Cluster Randomized Controlled Trial. *Annals of Emergency Medicine* 2022; **80(4 Supplement)**: S2-S3.
163. Rodriguez RM, Eucker SA, Rafique Z, et al. Promotion of Influenza Vaccination in the Emergency Department. *NEJM Evidence* 2024; **3**(4): 1-9.
164. Ronzani P, Panizza F, Martini C, Savadori L, Motterlini M. Countering vaccine hesitancy through medical expert endorsement. *Vaccine* 2022; **40(32)**: 4635-43.
165. Saaksvuori L, Betsch C, Nohynek H, Salo H, Sivela J, Bohm R. Information nudges for influenza vaccination: Evidence from a large-scale cluster-randomized controlled trial in Finland. *PLoS medicine* 2022; **19(2)**: e1003919.
166. Saccardo S, Dai H, Han MA, Vangala S, Hoo J, Fujimoto J. Field testing the transferability of behavioural science knowledge on promoting vaccinations. *Nat* 2024; **14**: 14.
167. Saitoh A, Sato I, Shinozaki T, Kamiya H, Nagata S. Effect of stepwise perinatal immunization education: A cluster-randomized controlled trial. *Vaccine* 2017; **35**(12): 1645-51.
168. Santa Maria D, Markham C, Misra SM, et al. Effects of a randomized controlled trial of a brief, student-nurse led, parent-based sexual health intervention on parental protective factors and HPV vaccination uptake. *BMC Public Health* 2021; **21**(1): 585.
169. Scarinci IC, Hansen B, Kim YI. HPV vaccine uptake among daughters of Latinx immigrant mothers: Findings from a cluster randomized controlled trial of a community-based, culturally relevant intervention. *Vaccine* 2020; **38**(25): 4125-34.
170. Scott VP, Opel DJ, Reifler J, et al. Office-Based Educational Handout for Influenza Vaccination: A Randomized Controlled Trial. *Pediatrics* 2019; **144**(2): 08.
171. Shegog R, Savas LS, Healy CM, et al. AVPCancerFree: impact of a digital behavior change intervention on parental HPV vaccine –related perceptions and behaviors. *Human vaccines and immunotherapeutics* 2022.
172. Shen Y, Wang J, Nicholas S, et al. Effectiveness of financial incentives on influenza vaccination among older adults in China: a randomized clinical trial. *Clin Microbiol Infect* 2024; **08**: 08.
173. Shourie S, Jackson C, Cheater FM, et al. A cluster randomised controlled trial of a web based decision aid to support parents' decisions about their child's Measles Mumps and Rubella (MMR) vaccination. *Vaccine* 2013; **31**(50): 6003-10.
174. Si M, Su X, Jiang Y, et al. Effect of an IMB Model-Based Education on the Acceptability of HPV Vaccination Among College Girls in Mainland China: A Cluster RCT. *Cancer Control* 2022; **29**: 10732748211070719.
175. Sitler LL. The effectiveness of combined appointments and influenza immunization rates in a rural WIC population. *Dissertation Abstracts International: Section B: The Sciences and Engineering* 2018; **78**(11-B(E)): No-Specified.
176. Stockwell MS, Hofstetter AM, DuRivage N, et al. Text message reminders for second dose of influenza vaccine: a randomized controlled trial. *Pediatrics* 2015; **135**(1): e83-91.
177. Stockwell MS, Kharbanda EO, Martinez RA, et al. Text4Health: Impact of Text Message Reminder-Recalls for Pediatric and Adolescent Immunizations. *Am J Public Health* 2012; **102**(2): E15-E21.
178. Stockwell MS, Kharbanda EO, Martinez RA, Vargas CY, Vawdrey DK, Camargo S. Effect of a text messaging intervention on influenza vaccination in an urban, low-income pediatric and adolescent population: a randomized controlled trial. *JAMA* 2012; **307**(16): 1702-8.
179. Stockwell MS, Shone LP, Nekrasova E, et al. Text Message Reminders for the Second Dose of Influenza Vaccine for Children: an RCT. *Pediatrics* 2022.
180. Stockwell MS, Westhoff C, Kharbanda EO, et al. Influenza vaccine text message reminders for urban, low-income Pregnant individuals: a randomized controlled trial. *Am J Public Health* 2014; **104 Suppl 1**: e7-12.

181. Stolpe S, Choudhry NK. Effect of Automated Immunization Registry-Based Telephonic Interventions on Adult Vaccination Rates in Community Pharmacies: A Randomized Controlled Trial. *J Manag Care Spec Pharm* 2019; **25**(9): 989-94.
182. Strathdee SA, Abramovitz D, Harvey-Vera AY, et al. A Brief Peer-Led Intervention to Increase Covid-19 Vaccine Uptake Among People Who Inject Drugs in San Diego County: Results From a Pilot Randomized Controlled Trial. *Open forum infect* 2023; **10**(8): ofad392.
183. Stuck AE, Moser A, Morf U, et al. Effect of health risk assessment and counselling on health behaviour and survival in older people: a pragmatic randomised trial. *PLoS Med* 2015; **12**(10): e1001889.
184. Suh CA, Saville A, Daley MF, et al. Effectiveness and net cost of reminder/recall for adolescent immunizations. *Pediatrics* 2012; **129**(6): e1437-45.
185. Suzuki Y, Sukegawa A, Ueda Y, et al. The Effect of a Web-Based Cervical Cancer Survivor's Story on Parents' Behavior and Willingness to Consider Human Papillomavirus Vaccination for Daughters: Randomized Controlled Trial. *JMIR Public Health Surveill* 2022; **8**(5): 15.
186. Sweeney JB. An evaluation of an intervention for hpv risk reduction among college-aged women. *Dissertation Abstracts International: Section B: The Sciences and Engineering* 2014; **75**(1-B(E)): No-Specified.
187. Szilagyi P, Albertin C, Gurfinkel D, et al. Effect of State Immunization Information System Centralized Reminder and Recall on HPV Vaccination Rates. *Pediatrics* 2020; **145**(5): 05.
188. Szilagyi PG, Albertin C, Casillas A, et al. Effect of Patient Portal Reminders Sent by a Health Care System on Influenza Vaccination Rates: A Randomized Clinical Trial. *JAMA Intern Med* 2020; **180**(7): 962-70.
189. Szilagyi PG, Albertin C, Humiston SG, et al. A randomized trial of the effect of centralized reminder/recall on immunizations and preventive care visits for adolescents. *Acad Pediatr* 2013; **13**(3): 204-13.
190. Szilagyi PG, Albertin CS, Saville AW, et al. Effect of State Immunization Information System Based Reminder/Recall for Influenza Vaccinations: a Randomized Trial of Autodialer, Text, and Mailed Messages. *Journal of pediatrics* 2020; **221**: 123-31.e4.
191. Szilagyi PG, Duru OK, Casillas A, et al. Text vs Patient Portal Messaging to Improve Influenza Vaccination Coverage: A Health System-Wide Randomized Clinical Trial. *JAMA Intern Med* 2024; **18**: 18.
192. Szilagyi PG, Humiston SG, Gallivan S, Albertin C, Sandler M, Blumkin A. Effectiveness of a citywide patient immunization navigator program on improving adolescent immunizations and preventive care visit rates. *Arch Pediatr Adolesc Med* 2011; **165**(6): 547-53.
193. Szilagyi PG, Schaffer S, Barth R, et al. Effect of telephone reminder/recall on adolescent immunization and preventive visits: results from a randomized clinical trial. *Arch Pediatr Adolesc Med* 2006; **160**(2): 157-63.
194. Szilagyi PG, Schaffer S, Rand CM, et al. Impact of elementary school-located influenza vaccinations: A stepped wedge trial across a community. *Vaccine* 2018; **36**(20): 2861-9.
195. Szilagyi PG, Schaffer S, Rand CM, et al. Text Message Reminders for Child Influenza Vaccination in the Setting of School-Located Influenza Vaccination: A Randomized Clinical Trial. *Clin Pediatr (Phila)* 2019; **58**(4): 428-36.
196. Tentori K, Pighin S, Giovanazzi G, Grignolio A, Timberlake B, Ferro A. Nudging Covid-19 Vaccine Uptake by Changing the Default: A Randomized Controlled Trial. *Medical Decision Making* 2022; **42**(6): 837-41.
197. Terrell-Perica SM, Effler PV, Houck PM, Lee L, Crosthwaite GH. The effect of a combined influenza/pneumococcal immunization reminder letter. *Am J Prev Med* 2001; **21**(4): 256-60.
198. Thilly N, Michel M, Simon M, et al. Effectiveness of a School- and Primary Care-Based HPV Vaccination Intervention: The PrevHPV Cluster Randomized Trial. *JAMA Netw Open* 2024; **7**(5): e2411938.
199. Tiro JA, Sanders JM, Pruitt SL, et al. Promoting HPV Vaccination in Safety-Net Clinics: A Randomized Trial. *Pediatrics* 2015; **136**(5): 850-9.
200. Topp L, Day CA, Wand H, et al. A randomised controlled trial of financial incentives to increase hepatitis B vaccination completion among people who inject drugs in Australia. *Prev Med* 2013; **57**(4): 297-303.
201. Tubiana S, Labarere J, Levraut J, et al. Effectiveness of a multifaceted informational-based and text message reminders on pneumococcal and influenza vaccinations in hospital emergency departments: A cluster-randomized controlled trial. *Vaccines* 2021; **9(9) (no pagination)**(962).
202. Tull F, Borg K, Knott C, et al. Short Message Service Reminders to Parents for Increasing Adolescent Human Papillomavirus Vaccination Rates in a Secondary School Vaccine Program: A Randomized Control Trial. *J Adolesc Health* 2019; **65**(1): 116-23.
203. Ueberroth BE, Labonte HR, Wallace MR. Impact of Patient Portal Messaging Reminders with Self-Scheduling Option on Influenza Vaccination Rates: a Prospective, Randomized Trial. *Journal of general internal medicine* 2022; **37**(6): 1394-9.
204. Usami T, Hashiguchi M, Kouhara T, Ishii A, Nagata T, Mochizuki M. Impact of community pharmacists advocating immunization on influenza vaccination rates among the elderly. *Yakugaku Zasshi* 2009; **129**(9): 1063-8.

205. Vanderpool RC, Cohen E, Crosby RA, et al. "1-2-3 Pap" Intervention Improves HPV Vaccine Series Completion among Appalachian Women. *J Commun* 2013; **63**(1): 95-115.
206. Vivier PM, Alario AJ, O'Haire C, Dansereau LM, Jakum EB, Peter G. The impact of outreach efforts in reaching underimmunized children in a Medicaid managed care practice. *Arch Pediatr Adolesc Med* 2000; **154**(12): 1243-7.
207. Wang ZX, Lau JTF, Ip TKM, et al. Two Web-Based and Theory-Based Interventions With and Without Brief Motivational Interviewing in the Promotion of Human Papillomavirus Vaccination Among Chinese Men Who Have Sex With Men: Randomized Controlled Trial. *J Med Internet Res* 2021; **23**(2): 17.
208. Wang ZX, Zhang QP, Wong MCS, Yu FY, Ye DH, Mo KH. A trans-theoretical model-based intervention delivered by fully automated chatbot was effective in increasing seasonal influenza vaccination uptake among community-living older adults in china - findings of a randomized controlled trial. *Int J Behav Med* 2023; **30**: S36-S7.
209. Weaver T, Metrebian N, Hellier J, et al. Use of contingency management incentives to improve completion of hepatitis B vaccination in people undergoing treatment for heroin dependence: a cluster randomised trial. *Lancet* 2014; **384**(9938): 153-63.
210. Wijesundara JG, Ito Fukunaga M, Ogarek J, et al. Electronic Health Record Portal Messages and Interactive Voice Response Calls to Improve Rates of Early Season Influenza Vaccination: Randomized Controlled Trial. *Journal of medical Internet research* 2020; **22(9)**: e16373.
211. Winston CA, Mims AD, Leatherwood KA. Increasing pneumococcal vaccination in managed care through telephone outreach. *Am J Manag Care* 2007; **13**(10): 581-8.
212. Wiseman P. A Study to Determine the Preliminary Effects of a Theory-Based Intervention (SayNo2Flu) Combined with the Use of Mobile Technology on Parents' Influenza Prevention Beliefs and Behaviors in a Primary Care Setting. *Dissertation Abstracts International: Section B: The Sciences and Engineering* 2016; **76**(9-B(E)): No-Specified.
213. Wong VWY, Fong DYT, Lok KYW, et al. Brief education to promote maternal influenza vaccine uptake: A randomized controlled trial. *Vaccine* 2016; **34**(44): 5243-50.
214. Wouters K, Leuridan E, Van Herck K, et al. Compliance and immunogenicity of two hepatitis B vaccination schedules in sex workers in Belgium. *Vaccine* 2007; **25**(10): 1893-900.
215. Wright A, Poon EG, Wald J, et al. Randomized controlled trial of health maintenance reminders provided directly to patients through an electronic PHR. *Journal of General Internal Medicine* 2012; **27**(1): 85-92.
216. Wynn CS, Catallozzi M, Kolff CA, et al. Personalized Reminders for Immunization Using Short Messaging Systems to Improve Human Papillomavirus Vaccination Series Completion: Parallel-Group Randomized Trial. *JMIR Mhealth Uhealth* 2021; **9**(12): e26356.
217. Xu J, Tang W, Qiu W, et al. Effects of mobile APP for immunization on vaccination compliance of migrant children in southwest China: A community trial study. *Hum Vaccin Immunother* 2022; **18**(7): 2135853.
218. Yeung KHT, Tarrant M, Chan KCC, Tam WH, Nelson EAS. Increasing influenza vaccine uptake in children: a randomised controlled trial. *Vaccine* 2018; **36**(37): 5524-35.
219. Yokum D, Lauffenburger JC, Ghazinouri R, Choudhry NK. Letters designed with behavioural science increase influenza vaccination in Medicare beneficiaries. *Nat* 2018; **2**(10): 743-9.
220. Yudin MH, Mistry N, De Souza LR, et al. Text messages for influenza vaccination among Pregnant individuals: A randomized controlled trial. *Vaccine* 2017; **35**(5): 842-8.
221. Zhang CQ, Zhang R, Chung PK, et al. Promoting influenza prevention for elderly people in Hong Kong using health action process approach: study protocol. *BMC Public Health* 2018; **18**: 9.
222. Zhang SX, Shoptaw S, Reback CJ, Yadav K, Nyamathi AM. Cost-effective way to reduce stimulant-abuse among gay/bisexual men and transgender women: a randomized clinical trial with a cost comparison. *Public Health* 2018; **154**: 151-60.
223. Zhang X, Chen H, Zhou J, Huang Q, Feng XY, Li J. Impact of web-based health education on HPV vaccination uptake among college girl students in Western and Northern China: a follow-up study. *BMC Womens Health* 2022; **22**(1): 46.
224. Zuniga de Nuncio ML, Nader PR, Sawyer MH, De Guire M, Prislin R, Elder JP. A prenatal intervention study to improve timeliness of immunization initiation in Latino infants. *J Community Health* 2003; **28**(2): 151-65.
